# N-Substituted-2-(9H-Xanthen-9-yl)acetamide Derivatives Induce In Vitro Colon Cancer Cell Death via TASK-1 Inhibition: Lead Compounds for Further Optimization as TASK-1-Targeted Therapeutics in Colorectal Cancer

**DOI:** 10.3390/ijms27094069

**Published:** 2026-05-01

**Authors:** Abdulaziz H. Al Khzem, S. M. El Rayes, Ibrahim A. I. Ali, Walid Fathalla, Mansour S. Alturki, Nada Tawfeeq, Saeed M. Tayeb, Abdulelah A. Alfattani, Saad M. Wali, Firdos A. Khan, Abdulmalik M. Alqarni, Faheem H. Pottoo, Dania Hussein, Mohamed S. Gomaa

**Affiliations:** 1 Department of Pharmaceutical Chemistry, College of Pharmacy, Imam Abdulrahman Bin Faisal University, P.O. Box 1982, Dammam 31441, Saudi Arabianztawfeeq@iau.edu.sa (N.T.); msmmansour@iau.edu.sa (M.S.G.); 2 Department of Chemistry, Faculty of Science, Suez Canal University, Ismailia 41522, Egypt; samir_elrayes@yahoo.com (S.M.E.R.);; 3 Department of Physical Sciences, Faculty of Engineering, Suez Canal University, Ismailia 41522, Egypt; 4 Pharmaceutical Sciences Department, College of Pharmacy, Umm Al-Qura University, Makkah 24381, Saudi Arabia; 5 Pharmacology and Toxicology Department, College of Pharmacy, Umm Al-Qura University, Makkah 24342, Saudi Arabia; 6 Department of Stem Cell Biology, Institute for Research & Medical Consultations (IRMC), Imam Abdulrahman Bin Faisal University, Dammam 31441, Saudi Arabia; 7 Department of Pharmacology, College of Pharmacy, Imam Abdulrahman Bin Faisal University, P.O. Box 1982, Dammam 31441, Saudi Arabia; dahussein@iau.edu.sa

**Keywords:** HCT-116 cells, K2P3.1 (TASK-1), potassium channels, apoptosis, molecular docking, KCNK3, cancer therapeutics

## Abstract

Colorectal cancer (CRC) is the third most prevalent cancer globally. TASK-1, encoded by the KCNK3 gene, is emerging as a putative target in cancer; it regulates resting membrane potential, cell proliferation, and apoptosis. A series of 27 novel xanthene derivatives, modified at position 9, were synthesized via azide coupling of 2-(9H-xanthen-9-yl)acetohydrazide with selected amines and amino acids, followed by hydrazine-mediated conversion to the corresponding hydrazides. The cytotoxic activity of selected compounds (**5a**–**5g**, **6a**–**6h**, **7b**, **7f**–**7h**) was evaluated against the HCT-116 cell line in vitro. In addition, molecular docking and molecular dynamics simulations were performed to investigate binding interactions and assess the stability of the protein–ligand complexes. Several compounds (**5f**, **5g**, **6c**, **6d**, **6f**, **6g**, **7b**, **7f**, and **7h**) exhibited moderate cytotoxic activity against HCT-116 cells (IC_50_: 66.97–99.62 µM), compared to cisplatin (IC_50_: 18.25 µM). Compound **7h** demonstrated pronounced antiproliferative effects, evidenced by DAPI staining showing chromatin condensation and apoptotic body formation, along with a marked reduction in cell count and coverage. Molecular docking indicated favorable binding within the TASK-1 potassium channel, and molecular dynamics simulations confirmed the stability of the protein–ligand complex, with consistent interactions, including a key hydrogen bond with Asn240. These findings support **7h** as a promising lead candidate. These findings identify xanthene-based derivatives as promising lead compounds for further optimization as TASK-1-targeted therapeutic candidates in colorectal cancer.

## 1. Introduction

Colorectal cancer (CRC) remains one of the most common malignancies worldwide and a leading cause of cancer-related mortality. According to the latest GLOBOCAN-based estimates, CRC ranks among the top three cancers in terms of incidence, with close to 2 million new cases annually, and remains the second leading cause of cancer deaths globally. Updated analyses of the Global Burden of Disease (GBD) 2019 and subsequent projections up to 2025 highlight a continuing increase in absolute case numbers, driven largely by population growth and aging, together with worrisome rises in early-onset CRC in many high- and middle-income countries. In the United States alone, approximately 153,000 new CRC cases and more than 52,000 deaths were estimated for 2023. These trends underscore the urgent need for new preventive and therapeutic strategies, including targeted agents that can modulate key signaling pathways in colorectal tumor cells [1,2].

Potassium channels (K^+^ channels) have emerged as promising therapeutic targets in cancer treatment due to their critical roles in various cellular processes crucial for tumorigenesis, including proliferation, apoptosis, and migration [3,4]. These channels constitute families like Kv (voltage-dependent channels), Kir (inward rectifier channels), and K2P (two-pore channel) and overstimulate in many cancer types to support cell proliferation and metastasis [5]. With respect to apoptosis, various K^+^ channel subunits have been implicated in its regulatory mechanisms, including the Kv1.1, Kv1.3, Kv1.5, Kv2.1, and Kv11.1 (hERG) subtypes. Additionally, other K^+^ channels, such as the two-pore TWIK-related acid-sensitive channels TASK-1 and TASK-3 and Kir channels, had also been associated with apoptotic processes [5].

Literature indicates that suppression of these channels can retard cancer cell division, stimulate cancer cell death, and improve the efficacy of chemotherapy [6]. Potassium channel subfamily k member 3 (KCNK3), also known as TASK-1, is a member of the two-pore domain potassium (K2P) channel family [7]. Several studies have found that KCNK3 expression is altered in various cancer types. In breast cancer, a single-nucleotide polymorphism (SNP) in KCNK3 has been linked to the development of secondary lymphedema following breast cancer surgery [8]. However, the exact mechanisms by which KCNK3 may contribute to breast cancer progression or lymphedema development are not yet fully understood. More recent studies have further implicated TASK-1 and other K2P channels in cancer biology and therapy resistance, including their roles in controlling membrane potential, migration, and adaptation to the tumor microenvironment. Such observations strengthen the rationale for exploring small-molecule TASK-1 modulators as potential adjuvant anticancer agents [3]. Despite these emerging observations, mechanistic and translational exploration of TASK-1 as a cancer target remains constrained by the limited number of diverse, well-characterized small-molecule chemotypes suitable for systematic optimization and cancer-oriented profiling. A key scientific need is therefore the development of new, synthetically tractable TASK-1-directed scaffolds that can be advanced through functional validation, selectivity testing across K2P channels, and structure-guided potency optimization.

**Figure 1:**
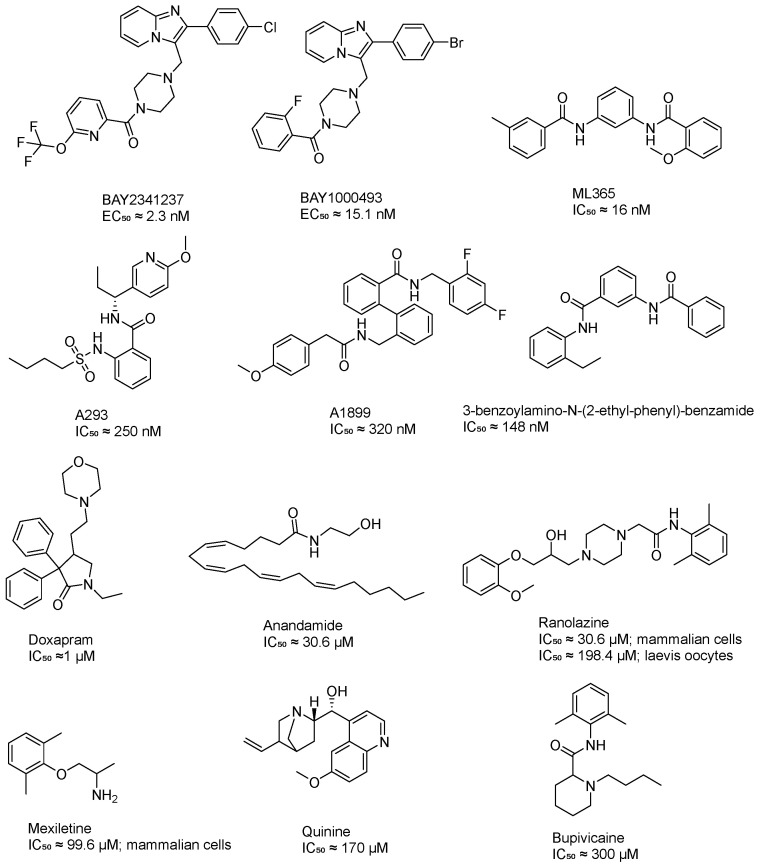
Chemical structure and potency of reported TASK-1 inhibitors.

Various inhibitors can modulate the activity of KCNK3 channels, for example, A293, anandamide, and Y-27632. These compounds have been utilized to investigate the functional roles of KCNK3 in various cellular processes and pathological conditions, particularly in the context of pulmonary arterial hypertension [9]. Moreover, some benzoxazine derivatives showed potassium channel modulation [10,11], e.g., 2,2-dimethyl-3,4-dihydro-2H-1,4-benzoxazines have been identified as potent openers of the pancreatic SUR1-type KATP channels [11]. Moreover, 2-(6-bromo-7-chloro-2, 2-dimethyl-2H-1, 3-benzoxazin-4-yl)pyridine 1-oxide, designed as K^+^ channel openers, exhibited more potent vasorelaxant activity and longer-lasting hypotensive effects than cromakalim [12]. From a pharmacological perspective, TASK-1 is unusually tractable among K2P channels: structural studies show that high-affinity inhibitors can bind within the intramembrane vestibule below the selectivity filter and exhibit very slow washout, consistent with inhibitor trapping by a lower gate (“X-gate”) in TASK channels. Reported TASK-1 inhibitors span subnanomolar to micromolar functional potency depending on scaffold and assay platform. Representative examples include the high-affinity inhibitors BAY1000493 and BAY2341237 [13], the bisamide tool compounds A1899 and ML365 [13,14], the experimental antiarrhythmic tool compound A293 [15], 3-benzoylamino-N-(2-ethyl-phenyl)-benzamide (IC50 of 148 nM) [16], and the clinically used respiratory stimulant doxapram [17]. In contrast, several clinically used or well-studied compounds exhibit comparatively lower potency towards TASK-1. For instance, ranolazine inhibits TASK-1 with an IC_50_ of 30.6 ± 3.7 µM in mammalian cells and 198.4 ± 1.1 µM in Xenopus laevis oocytes [18]; Anandamide, a known TASK-1 inhibitor, shows an IC_50_ of 99.6 µM in oocyte-based assays [19]; while quinine exhibits an IC_50_ of approximately 170 µM in Xenopus oocytes [19]. Similarly, mexiletine, a Class IB antiarrhythmic drug, inhibits human TASK-1 channels with an IC_50_ of 99.6 µM in mammalian cells [20], and bupivacaine demonstrates an IC_50_ of approximately 300 µM [19], These examples are summarized in Figure 1.

In this paper, we describe the synthesis, biological activity, and molecular modeling of a novel series of xanthene derivatives as potassium channel modulators and potential anticancer agents. In the field of medicinal chemistry, xanthenes and their derivatives are significant. Their pharmacological actions include antiviral, antibacterial, and anti-inflammatory and anticancer activities [21]. The literature survey continues to highlight the anticancer potential of xanthene-based scaffolds. Ebadi et al. reported novel xanthene-1,8-dione derivatives bearing benzylic ether side chains with potent cytotoxic activity against A549 lung carcinoma cells and supportive in silico DNA-binding studies. In a complementary review, the anticancer activity of xanthone derivatives was comprehensively summarized, emphasizing that subtle modifications in substitution patterns can markedly influence potency, selectivity, and mechanism of action. These findings support further exploration of xanthene-type pharmacophores as tunable anticancer agents [22].

**Figure 2:**
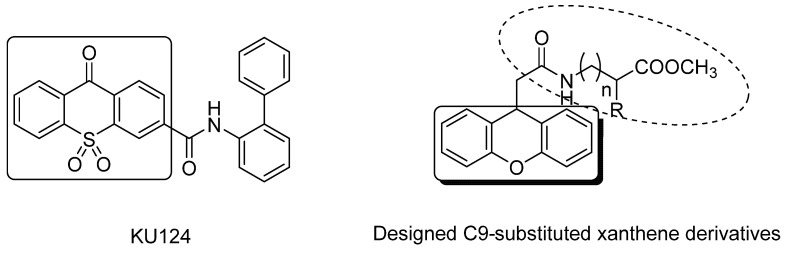
Design of the C-9-substituted xanthene derivatives.

Reported TASK-channel blockers frequently share physicochemical characteristics that are compatible with binding to largely hydrophobic inner-pore/vestibule environments, including prominent aromatic/hydrophobic groups combined with hydrogen-bond acceptors and other polar handles that can help position the ligand within the pore region. The xanthene scaffold provides a rigid, lipophilic, tricyclic aromatic framework that can serve as a compact hydrophobic “core,” while C-9 functionalization enables systematic tuning of size, polarity, hydrogen-bonding capacity, and conformational flexibility through appended amide/hydrazide side chains. Notably, a thioxanthene-based compound (KU124) has been reported as a TASK-1 inhibitor, supporting the broader compatibility of xanthene/thioxanthene tricyclic chemotypes with TASK-1 blockade. Based on these precedents and the availability of structural information for high-affinity TASK-1 inhibitors bound in the vestibule, we designed C-9-substituted xanthene acetamide/hydrazide derivatives as a modular scaffold to explore whether a tricyclic aromatic core with tunable polar side chains can furnish cancer-oriented starting points for TASK-1-directed optimization (Figure 2).

## 2. Results

### 2.1. Chemical Synthesis

Recent SAR analyses of xanthene and xanthone derivatives have emphasized that the pharmacological profile of these scaffolds is strongly governed by the nature and topology of substituents at the benzylic C-9 position and on the aromatic rings, which modulate both binding to diverse protein targets and physicochemical properties such as lipophilicity and ionization. In particular, functionalization at C-9 through amide or amino-alkyl linkers has been associated with enhanced anticancer potency and the possibility to tune target selectivity. In this context, introducing a 2-(9H-xanthen-9-yl)acetamide linker that can be decorated with aliphatic amines, amino esters, and hydrazides provides a modular and, to the best of our knowledge, previously unexplored way to engineer both target engagement and ADME-relevant properties around the xanthene pharmacophore [21,23].

**Scheme 1:**
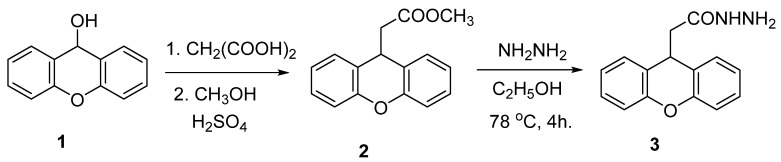
Preparation of 2-(9*H*-xanthen-9-yl)acetohydrazide (**3**).

For the change at the xanthene system, 2-(9H-xanthen-9-yl)acetohydrazide (**3**) turned out to be an intriguing structure. Using a series of sequential steps, 2-(9H-xanthen-9-yl)acetohydrazide (3) was synthesized by first reacting 9-hydroxyxanthene 1 with malonic acid. 9H-xanthen-9-ol 1 is highly reactive towards nucleophiles; it reacted with malonic acid in dry benzene at 90 °C for 4 h in the presence of a pyridine via the in situ generated malonic acid derivative, and the subsequent heating (decarboxylation) yields 2-(9H-xanthen-9-yl) acetic acid, which was then esterified to produce methyl 2-(9H-xanthen-9-yl)acetate (2), finally, hydrazinolyzing 2 in ethanol with hydrazine hydrate under reflux conditions to produce hydrazide **3**, Scheme 1.

2-(9*H*-xanthen-9-yl)acetohydrazide (**3**) is an excellent precursor of the structure modification of the xanthene ring system via azide coupling method with amines and amino acids. This method gives us a great advantage of conjunction of amines and amino acids with a wide diversity of lipophilic and hydrophilic behavior added to xanthene system for biological evaluation. The azide coupling method is a well-recognized method in organic synthesis and amino acid coupling.

**Scheme 2:**
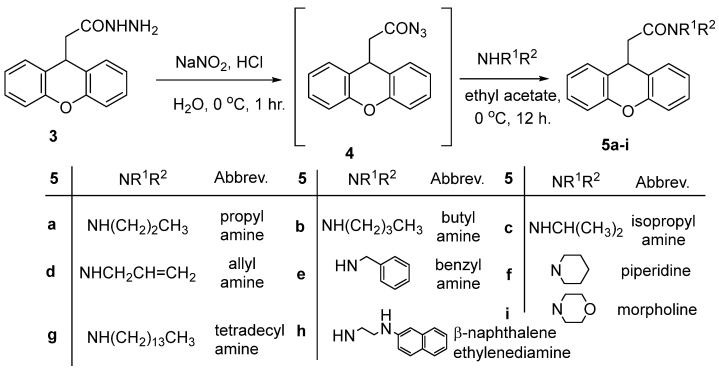
Preparation of *N*-alkyl-2-(9*H*-xanthen-9-yl) acetamides **5a**–**i**.

2-(9*H*-xanthen-9-yl)acetohydrazide (**3**) is an excellent starting point for the azide coupling approach, which involves changing the structure of the xanthene ring system using amines or amino acids. The conjugation of amines and amino acids with a wide range of lipophilic and hydrophilic behavior to the xanthene system for biological evaluation would be extremely beneficial. 2-(9H-xanthen-9-yl)acetohydrazide (**3**) is an excellent starting point for the azide coupling approach, which involves transient generation of the corresponding acyl azide 4 followed by in situ reaction with amines or amino acid esters. Compared with classical carbodiimide-based amidation of xanthene carboxylic acids, the acyl-azide route offers several advantages that are particularly relevant for our system: (i) the coupling is performed under very mild, low-temperature conditions (−5 °C) that are compatible with sensitive xanthene cores and unprotected amino acid side chains; (ii) the method proceeds without added coupling reagents or excess dehydrating agents, minimizing side reactions such as O-acylation, over-activation, or urea by-products; (iii) the only stoichiometric by-product is nitrogen gas, which simplifies work-up and facilitates scale-up; and (iv) high levels of chiral integrity and minimal epimerization at the α-stereocenters of amino acids have been documented for acyl-azide-based peptide couplings. Furthermore, despite the extensive use of the azide method in peptide chemistry, its application to the late-stage diversification of xanthene scaffolds by direct coupling of amino acid esters and diverse amines at C-9 appears to be unprecedented, adding a measure of synthetic originality to the present study [24,25]. Although a route based on direct amidation of 2-(9H-xanthen-9-yl)acetic acid with the corresponding amines or amino acids is conceptually shorter in terms of formal steps, our preliminary attempts under standard carbodiimide-mediated conditions gave lower conversions, inseparable side-products, and partial epimerization for amino acid substrates, whereas the present acyl-azide protocol furnished the desired libraries **5a–i** and **6a–h** in consistently higher yields and with operational simplicity. Thus, at −5 °C, the 2-(9*H*-xanthen-9-yl)acetohydrazide (**3**) reacted with sodium nitrite in the presence of hydrochloric acid for 1 h. This produced 2-(9*H*-xanthen-9-yl)acetyl azide (**4**). Azide **4** was used in situ without separation or purification and extracted with ethyl acetate in a one-pot technique. The ethyl acetate solution of **4** was added to ethyl acetate solution of amines: propyl amine, butyl amine, isopropyl amine, allyl amine, benzyl amine, piperidine, tetradecyl amine, β-naphthalene ethylene diamine, and was kept at −5 °C for 24 h to afford *N*-alkyl-2-(9H-xanthen-9-yl)acetamides **5a**–**i** in 59–88% yields, Scheme 2.

**Scheme 3:**
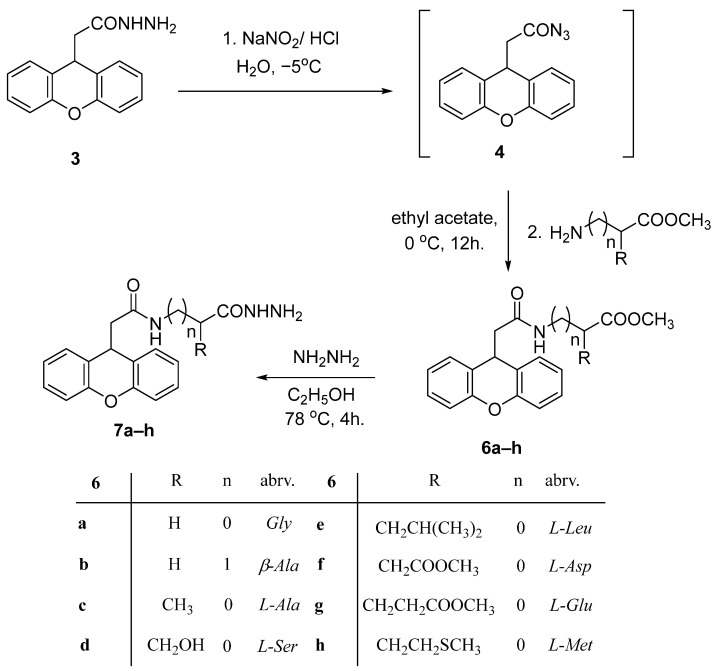
Preparation of methyl 2-(2-(9H-xanthen-9-yl)acetamido) alkanoates **6a–h** and 2-(2-(9H-xanthen-9-yl)acetamido) alkanhydrazides **7a**–**h**.

A variety of amines were successfully conjugated to the xanthene ring structure using the azide coupling approach. We will use this technique to connect amino acids next. In order to obtain methyl 2-(2-(9H-xanthen-9-yl)acetamido)alkanoates 6a–h, the ethyl acetate of azide 4 was thus added to the ethyl acetate solution of amino acid ester hydrochlorides: glycine, *β*-alanine, *L*-alanine, *L*-serine, *L*-leucine, *L*-aspartic, *L*-glutamic, and *L*-methionine, in the presence of triethyl amine. The mixture was then maintained at −5 °C for 24 h. to afford **6a–h** in 54–85% yields, Scheme 3.

Because of their diverse reactivity and ability to function as synthons for a wide range of complex molecules with a wide range of biological activities, heterocyclic hydrazides are an important class of chemical structures. In addition, multiple reports have demonstrated that converting poorly soluble carboxylic acid or amide derivatives into hydrazides can significantly improve aqueous solubility and formulation behavior by introducing additional hydrogen-bond donors and acceptors, while retaining or even enhancing biological activity. Tanaka et al. recently described a hydrazide-mediated solubilizing strategy for poorly soluble peptides using a dialkoxybenzaldehyde linker, which increased water solubility and enabled efficient handling of highly lipophilic sequences. Related hydrazide and hydrazone systems have also been widely explored in medicinal chemistry as bioisosteric replacements of amide or carboxylate functions with improved pharmacokinetic profiles. Motivated by these observations and by the need to counterbalance the intrinsic lipophilicity of the xanthene core, we therefore prepared a congener series of 2-(2-(9H-xanthen-9-yl)acetamido)alkanhydrazides **7a**–**h** to probe whether introducing terminal hydrazide functionality could improve solubility and facilitate future optimization of the most active analogues [26,27,28]. Thus, after four hours of reflux-condition reaction between the amino acid derivatives 6a–h and hydrazine hydrate in ethanol, 2-(2-(9H-xanthen-9-yl)acetamido) alkanhydrazides 7a–h were produced with 61–86% yields.

Based on ^1^H, ^13^C NMR and physicochemical studies, the structures of N-alkyl-2-(9H-xanthen-9-yl)acetamides **5a–i**, methyl 2-(2-(9H-xanthen-9-yl)acetamido) alkanoates **6a–h**, and 2-(2-(9H-xanthen-9-yl)acetamido) alkanhydrazides 7a–h were assigned. Signals at δ 1.01, 2.42, 3.97–4.08, 4.57–4.64, and 4.98 ppm in the ^1^H NMR spectrum of N-isopropyl-2-(9H-xanthen-9-yl)acetamide (**5c**) were found to correspond to the 2CH3, CH_2_CO, NCH, CH, and NH groups, respectively. The CH_2_CO, NCH, CH, and C=O groups were represented by signals at δ 36.4, 41.5, 48.4, and 169.5 ppm in the ^13^C NMR spectrum of 5c, respectively. However, the 1H NMR spectrum of methyl 2-(2-(9H-xanthen-9-yl)acetamido) acetate (6a); glycine revealed signals for CH_2_CO, OCH_3_, NCH_2_, CH, and NH at δ 2.57, 3.73, 3.98, 4.61, and 5.69 ppm, respectively. Signals for CH2CO, NCH2, CH, OCH3, C=O, and C=O were detected at δ 36.0, 41.3, 47.9, 52.4, 170.0, and 170.4 ppm in the ^13^C NMR spectrum of **6a**, respectively.

### 2.2. Impact of Compounds on Cell Viability

**Figure 3:**
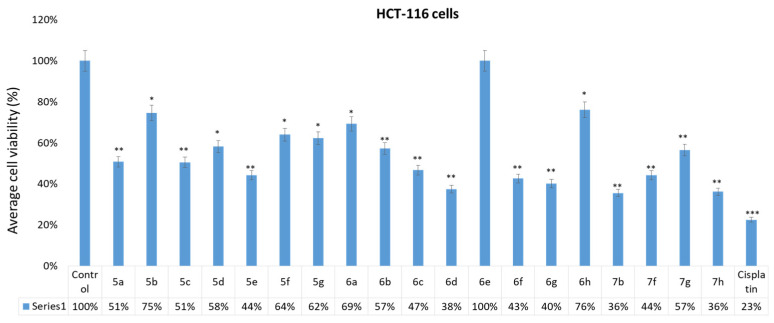
Effect of compounds (**5a**, **5b**, **5c**, **5d**, **5e**, **5f**, **5g**, **6a**, **6b**, **6c**, **6d**, **6e**, **6f**, **6g**, **6h**, **7b**, **7f**, **7g**, **7h**) on the HCT-116 cells showing average cell viability post 48 h of treatments. * *p* < 0.05; ** *p* < 0.01, *** *p* < 0.001.

In line with the above design, the antiproliferative activity of selected representatives from the three series was evaluated against HCT-116 cells to derive an initial SAR focused on the C-9 side-chain motif. The impact of compounds (**5a**, **5b**, **5c**, **5d**, **5e**, **5f**, **5g**, **6a**, **6b**, **6c**, **6d**, **6e**, **6f**, **6g**, **6h**, **7b**, **7f**, **7g**, **7h**) on HCT-116 cells was investigated by MTT assay. After 48 h treatment with the tested compounds, the cell viability results showed that compounds **5f**, **5g**, **6c**, **6d**, **6f**, **6g**, **7b**, **7f**, and **7h** possessed good cytotoxic activity with IC50 ranging from 66.97 to 99.62 µM, whereas compounds **5a**, **5c**, **5d**, **5e**, **6a**, and **6b** exhibited moderate activity with IC50 ranging from 101.76 to 135.19 µM. Compounds **5b** and **7g** and showed weak inhibition activity with IC50 of 210.77–242.78 µM, respectively, while compound **6e** was the only compound to show no cytotoxic effect on the tested cancer cells (Figure 3). Cisplatin with IC50 of 18.25 µM was used as standard drug. The inhibitory concentration (IC50) values of the samples on the cancer cells HCT-116 are presented in Table 1. Although this is an early-stage, focused library, several SAR trends can be discerned. Within the N-alkyl amide series, increasing the size and lipophilicity of the alkyl substituent generally improved potency, as illustrated by the superior activity of the long-chain tetradecyl analogue **5g** (IC50 77.56 µM) relative to the short-chain propyl and butyl analogues **5a** and **5b** (IC50 103.28 and 242.78 µM, respectively). In addition, incorporation of cyclic amines such as piperidine (5f, IC50 84.54 µM) maintained good activity, consistent with a tolerance for more bulky, basic side chains at this position. In the amino-acid-derived ester series **6a**–**h**, the *L*-alanine, *L*-serine, aspartate, and glutamate derivatives **6c**, **6d**, **6f**, and **6g** displayed better or comparable activity (IC50 75.35–99.62 µM) than the glycine or β-alanine analogues **6a** and **6b**, suggesting that both side-chain branching and additional polar or ionizable groups are beneficial. Finally, among the hydrazide series **7a**–**h**, the methionine-derived analogue **7h** and the γ-propionyl hydrazide **7b** were the most active (IC50 72.48 and 66.97 µM, respectively), pointing to a favorable combination of increased hydrogen-bonding capacity and a moderately flexible side chain. Taken together, these observations support the view that the xanthene core tolerates diverse C-9 substituents and that potency against HCT-116 cells can be fine-tuned by balanced modulation of side-chain lipophilicity and polarity at this single position.

### 2.3. Anti-Apoptotic Impact

**Figure 4:**
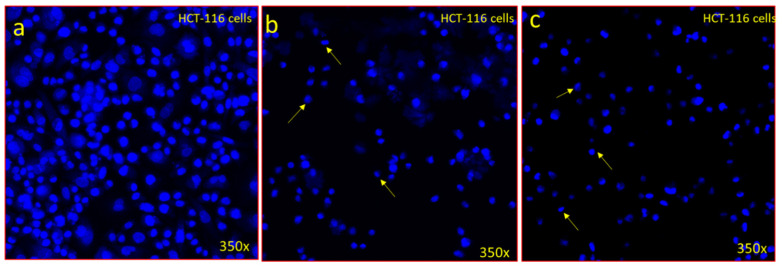
This shows the impact of compounds on DAPI stained cells where (**a**) is the control cells (cells are intact and healthy), treated with compound 2b (**b**), and compound **7h** (**c**) showing formation of apoptotic bodies (arrows). Image magnification 350×.

Apoptosis is a main pathway of cancer cell death. In order to assess the anti-apoptotic activity of our compounds, we have stained compound-treated HCT-116 cells with DAPI 4′,6-diamidino-2-phenylindole, which is used as marker for the apoptosis. The compounds (**7b** and **7h**) were selected for DAPI staining based on their highest cytotoxicity potential. The results showed that compounds (**7b** and **7h**) induced cell death (Figure 4). Moreover, chromatin condensation and formation of apoptotic bodies were observed (Figure 4b,c). The control cells (untreated) appeared healthy (Figure 4a).

### 2.4. Image Analysis

**Figure 5:**
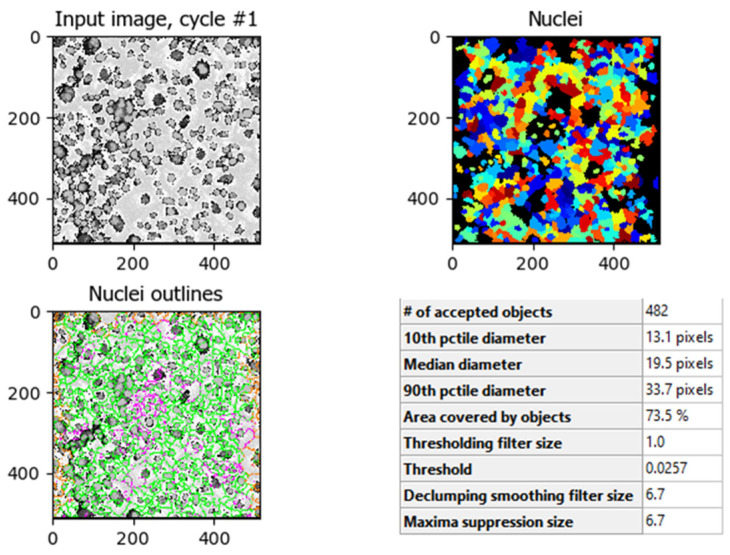
Nuclei count of DAPI stained control cells (untreated).

**Figure 6:**
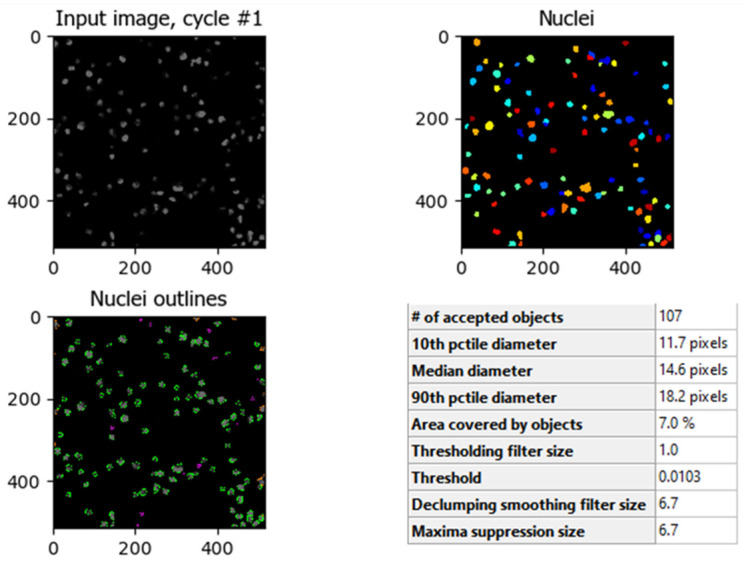
Nuclei count of DAPI stained cells treated with **7h** compound.

In the control sample (Figure 5), there is a high density of nuclei, with 482 nuclei detected, indicating a robust proliferation of HCT-116 cells under normal conditions. However, the cells treated with compound **7h** (Figure 6) show a significant reduction in the number of detected nuclei, down to 107 objects. This substantial reduction suggests that compound **7h** effectively inhibits cell proliferation, a key indicator of its anticancer activity.

The drastic reduction in both the number of nuclei and the total area covered by nuclei (from 73.5% in the control, Figure 5, to 7.0% in the treated sample, Figure 6) indicates that compound **7h** not only inhibits cell growth but also likely induces cell death. The smaller median diameter (14.6 pixels) and 90th percentile diameter (18.2 pixels) of the nuclei in the treated cells (Figure 6) compared to the control (Figure 5) could reflect nuclear condensation and fragmentation typically associated with apoptotic processes.

The decrease in the size distribution of nuclei after treatment (as seen in Figure 6, with smaller 10th, median, and 90th percentile diameters) suggests that compound **7h** may cause cell cycle arrest. The reduced size and number of nuclei could indicate that cells are arrested in a specific phase of the cell cycle, preventing further growth and division, thus contributing to its anticancer effects.

**Figure 7:**
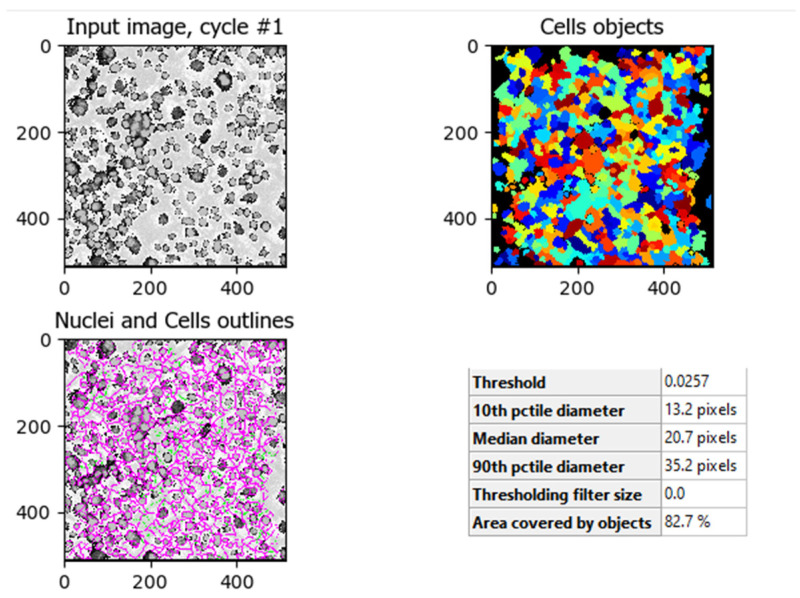
Cell count of DAPI stained control cells (untreated).

**Figure 8:**
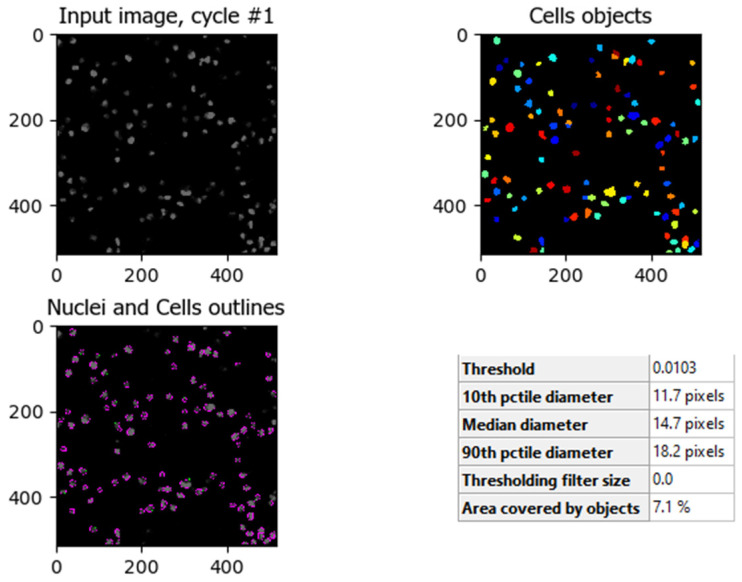
Cell count of DAPI stained cells treated with 7h compound.

The “Cells objects” panel in Figure 7 shows a high density of detected cells, represented by a variety of colors. The cells are closely packed, with many overlapping areas, which suggests a high level of cell proliferation typical of untreated HCT-116 cancer cells. The dense arrangement of cells indicates a healthy, proliferative state with minimal cell death. In Figure 8, the “Cells objects” panel displays a significantly reduced number of detected cells. The cells are well-separated, with much less overlap than in Figure 7. This sparse distribution suggests that compound **7h** effectively reduced the overall cell population, likely through inhibiting cell proliferation or inducing cell death.

The cells in Figure 7 cover a substantial area of the image, with 82.7% of the total area being occupied by cells. This high percentage of coverage indicates a dense cell culture with active proliferation, characteristic of untreated cancer cells, while the area covered by cells in Figure 8 is dramatically reduced to only 7.1%. This stark contrast in coverage reflects a significant loss of cell population due to the treatment with compound **7h**, highlighting its efficacy in reducing cell density and inhibiting cell growth.

### 2.5. Molecular Docking

**Figure 9:**
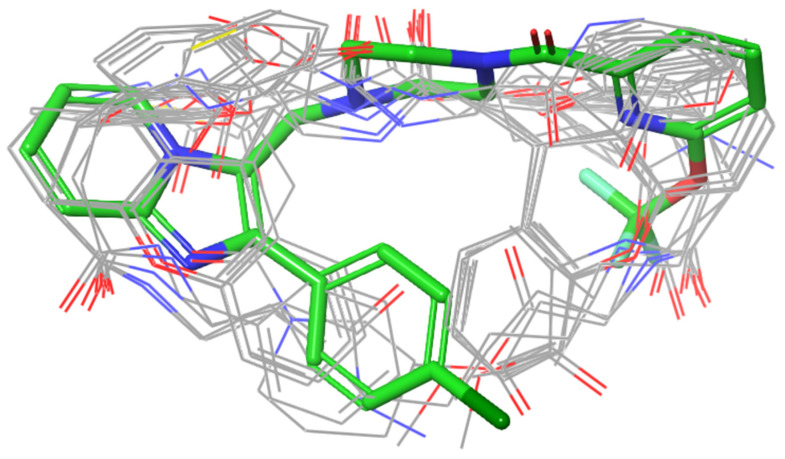
Binding pose superposition of the synthesized xanthene derivatives (line representation) and crystal ligand (stick representation) in potassium ion channel TASK-1 (PDB IDs: 6RV4) binding site.

The molecular docking studies in the TASK-1 channel were therefore used to complement the preliminary SAR derived from the HCT-116 assay and to rationalize how the diverse C-9 side chains in series 5–7 might modulate binding within the narrow, deeply buried binding pocket. The glide prediction of potassium ion channel TASK-1 binding was validated by redocking of the crystal ligand, which showed good accuracy, as evident from an RMSD of 2.1527 for the predicted binding pose relative to the crystal structure pose. The docking of our xanthene derivatives in the potassium ion channel TASK-1 revealed common and interesting poses. All inhibitors are well aligned in the narrow binding pocket and exhibited similar binding patterns that are closely similar to that of the crystal ligand (Figure 9).

The superposition of the binding poses of all inhibitors relative to the compound with the lowest experimental IC50 (7h) gave RMSD ranging from 0.3886 for compound **7g** to 2.3479 for compound **7b** (Table 2). This finding reflects the specificity of our xanthene structure scaffold in binding the TASK-1 binding site and validates this putative binding in a real environment.

**Figure 10:**
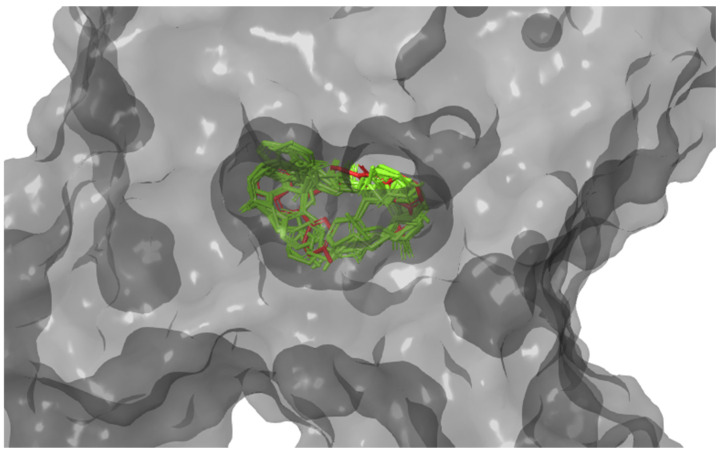
Surface representation of potassium ion channel TASK-1 (PDB IDs: 6RV4) showing the synthesized xanthene derivatives (green sticks) and the crystal ligand (red sticks) in the binding site.

The surface representation showed that all compounds, like the crystal ligand, occupied the deeply buried binding pocket that is accessed through a narrow substrate access channel, which emphasizes the role of the flexible chain of the compounds in reaching the binding site (Figure 10).

The calculated binding scores and binding energies reflected a favorable binding and affinity. The XP scores ranged from −11.114 to −7.6 for compounds **5g** and **5f,** respectively. The calculated binding energies showed good negative values ranging from −63.17 for compound **5g** to −16.39 for compound **5f**. The values for both XP scores and binding energy are comparable to those calculated for the crystal ligand, which scored −10.015 and −49.46 for both XP score and binding energy, respectively (Table 3).

**Figure 11:**
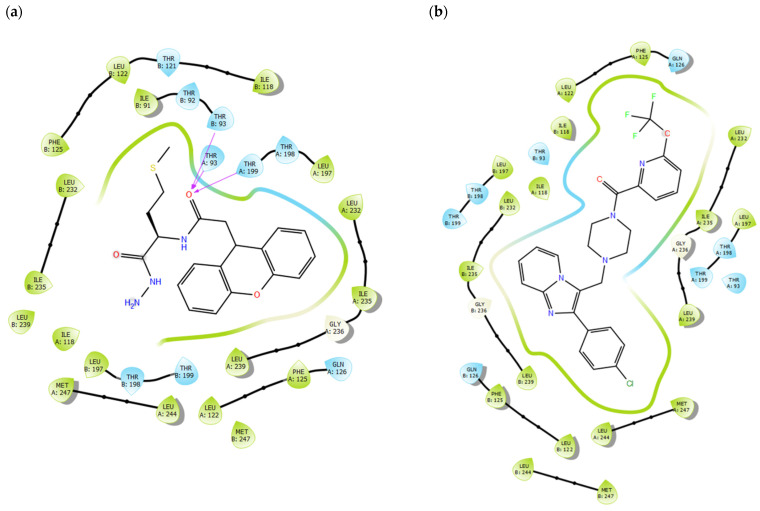
Two-dimensional ligand–protein binding interactions between (**a**) compound **7h** and (**b**) crystal ligand with potassium ion channel TASK-1 (PDB IDs: 6RV4) binding site. H bonds are represented as purple arrows.

**Figure 12:**
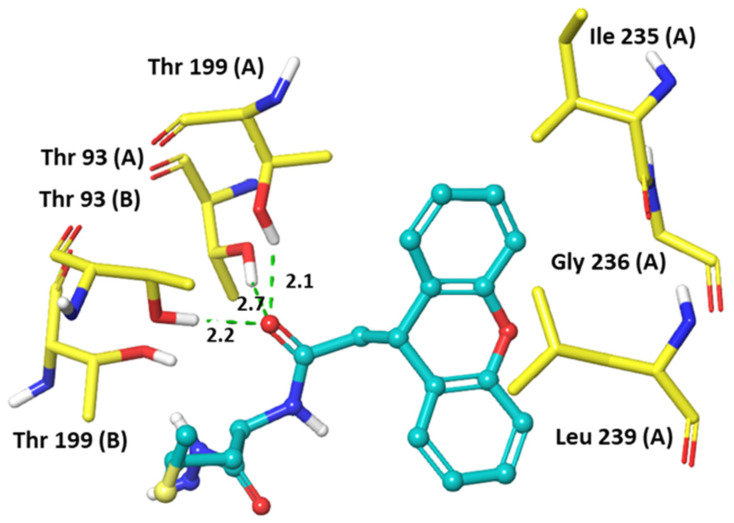
Three-dimensional representation of the binding interactions between compound **7h** as sticks (carbon atoms in cyan) and potassium ion channel TASK-1 (PDB IDs: 6RV4) binding site (chain A, chain B) shown as sticks (carbon atom in yellow). Potential electrostatic interactions are represented as green dotted lines and are measured in Angstrom.

The docking of compound **7h** showed that it was able to bind both chains of the K channel target. The compound occupied the binding pocket and established van der Waals interactions with its xanthene ring and Leu 232, Ile 235, Gly 236, Leu 239 from chain A, while the sidechain interacted with Ile 91, Thr 92, Thr 199, Leu 232, Ile 235, from chain B. Furthermore, **7h** was superior to the crystal ligand in forming H bond with its amide oxygen and Thr 93, Thr 199 from chain A, and Thr 93 from chain B (Figure 11 and Figure 12).

### 2.6. Molecular Dynamics Simulations

To complement the initial docking studies and evaluate the dynamic stability of the docked complex, a 100 ns all-atom molecular dynamics (MD) simulation was performed using Desmond in the NPT ensemble at 300 K. The simulated system contained 74,526 atoms, including 22,041 water molecules and appropriate counterions, with the protein described as a dimeric construct of 513 residues distributed over chains A and B and the bound small-molecule ligand (50 atoms, 27 heavy atoms).

**Figure 13:**
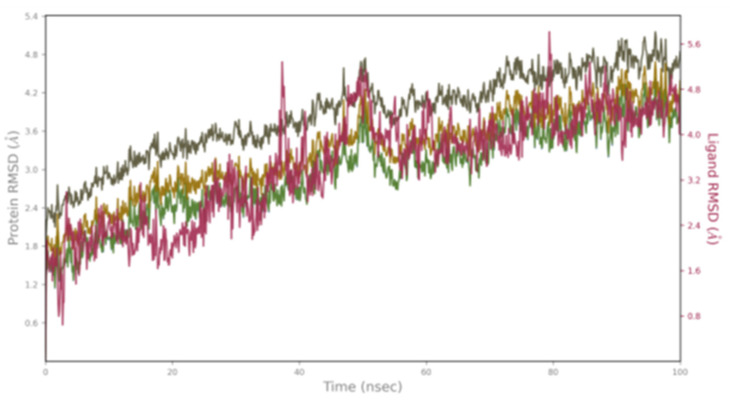
Root mean square deviation (RMSD) graphs for compound **7h**. The green graph shows fluctuations in the protein backbone from the initial reference point, while the red shows the ligand fluctuations. Mustard and grey graphs indicated fluctuations in the protein C-alpha atoms and side chains, respectively. The RMSD profile of the ligand relative to the protein binding pocket remained within 2–7 Å throughout the simulation, indicating stable accommodation within the binding site.

The backbone and ligand heavy-atom root mean square deviation (RMSD) profiles were monitored over the entire 0–100 ns trajectory to assess equilibration and global stability of the complex; complementary graphs for the stability of protein side chains and C-alpha atoms are also depicted (Figure 13). In the Desmond simulation interaction diagram, protein RMSD is computed after superposition of each frame on the reference backbone, whereas the “Lig fit Prot” metric reports the ligand RMSD after alignment on the protein backbone, thereby directly probing how tightly the ligand remains anchored within the binding pocket. The protein backbone RMSD remained within ~1–3 Å, indicating a well-equilibrated system without significant structural deviations.

**Figure 14:**
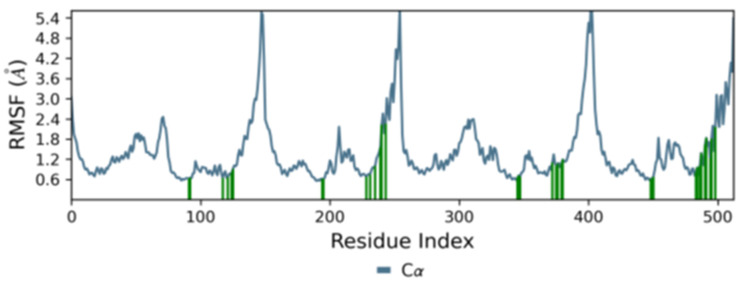
Root mean square fluctuation (RMSF) graphs for the protein complexed with compound **7h**. The blue graph shows fluctuations along the protein chain with peaks indicating areas of the protein that fluctuate the most during the simulation. Protein residues that interact with the ligand are marked with green-colored vertical bars.

Per-residue Cα root mean square fluctuation (RMSF) analysis was used to characterize local flexibility along the sequence and to distinguish rigid structural cores from more mobile loop and terminus regions (Figure 14). Peaks in the RMSF trace highlight residues with enhanced mobility, while vertical green bars identify residues that are in contact with the ligand at any point during the simulation, facilitating direct mapping of binding-site dynamics.

The secondary-structure analysis indicates that the protein maintains a predominantly α-helical fold throughout the trajectory, with an average helix content of 74.22% and no β-strand content (0.00% strand; 74.22% total secondary-structure elements). Monitoring secondary structure as a function of time and residue index confirms that the helical core remains well-defined over the simulated timescale, while any structural plasticity is largely confined to loop and terminal regions rather than extensive unfolding of the scaffold.

**Figure 15:**
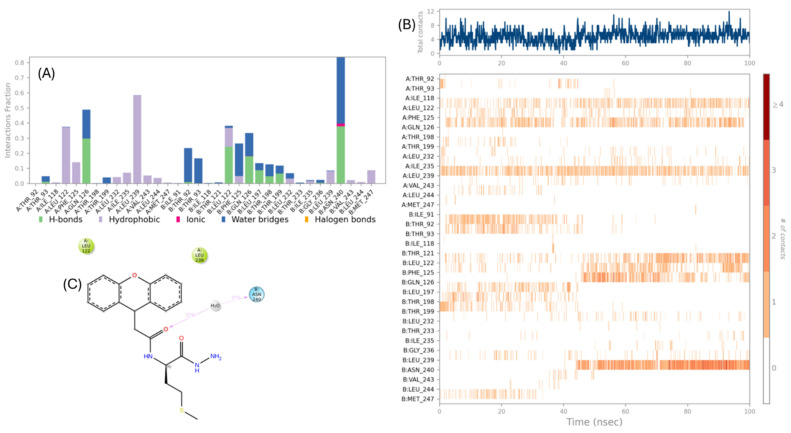
Interaction diagram of hit compound **7h** within the potassium ion channel TASK-1 (K2P3.1) (PDB IDs: 6RV4) binding pocket. (**A**) Interaction of compound **7h** with residues in each trajectory frame (**B**) The protein–ligand contacts showing the bonding interactions fraction and the nature of the interactions. (**C**) Graphical 2D illustration of compound **7h** interacting with the protein residues during MD simulation. H bonds are represented as purple arrows.

Protein–ligand contacts were quantified and categorized into hydrogen bonds, hydrophobic contacts, ionic interactions, water bridges, and halogen bonds using the simulation interaction diagram (Figure 15A). Protein–ligand interaction analysis revealed persistent hydrogen bonding, hydrophobic contacts, and water bridges throughout the trajectory.

A complementary contact timeline maps, for each frame, which residues are engaged with the ligand and with how many simultaneous contacts, providing a time-resolved picture of the interaction network (Figure 15B). The color intensity encodes the number of concurrent contacts, such that darker shades highlight residues that act as persistent anchors for the ligand along the trajectory and help to delineate a well-defined interaction hotspot within the binding pocket.

A two-dimensional schematic of ligand–protein interactions was generated to summarize, at the atomistic level, residues that remain engaged with the ligand for more than 30% of the simulation time across the 0–100 ns window (Figure 15C). In this representation, contacts are classified as hydrophobic, polar, water-mediated, or solvent exposure, and only interactions exceeding the 30% occupancy threshold are displayed, ensuring that the diagram focuses on the dominant, recurrent contacts rather than transient encounters.

The presence of multiple protein residues that satisfy this ≥30% occupancy criterion indicates the formation of a persistent interaction network around the ligand, consistent with a ligand that remains localized within a preferred binding region rather than diffusing freely through the solvent phase. This schematic therefore complements the contact timeline by highlighting the key residues that contribute most strongly to long-lived recognition of the ligand in the binding pocket.

Notably, residue Asn240 formed a highly persistent interaction with compound **7h**, maintaining a hydrogen bond and water bridge for ~80% of the simulation time, identifying it as a key anchoring residue (Figure 15).

**Figure 16:**
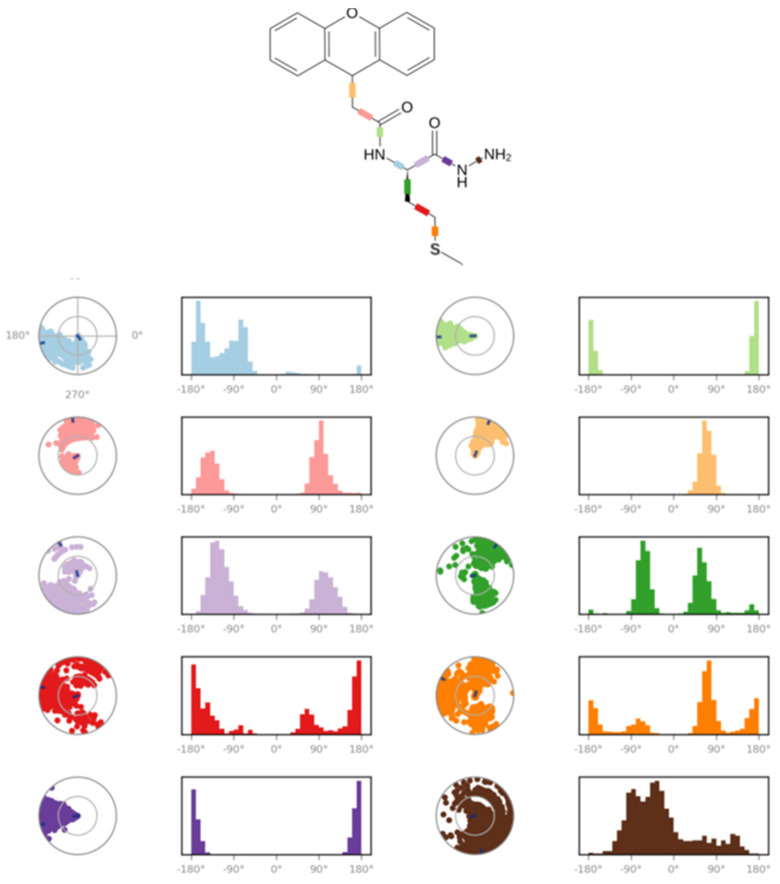
Ligand flexibility and torsional behavior of compound **7h**: (**top**) per-atom ligand RMSF mapped onto the 2D structure, highlighting more flexible versus rigid fragments; (**bottom**) torsion profile for each rotatable bond, combining radial plots of time evolution with histograms of torsion-angle populations and, when available, torsional potential curves, to assess conformational preferences and possible strain in the bound state.

Ligand atom-wise RMSF was calculated to dissect which parts of the small molecule are most flexible, with the results mapped onto the 2D structure to directly relate atomic fluctuations to specific fragments (Figure 16, top). This analysis is particularly informative for understanding how peripheral substituents, flexible linkers, or terminal groups contribute to entropic components of binding and whether certain moieties remain tightly packed against the binding pocket or sample broader conformational space.

The ligand torsion profile further tracks the time evolution and probability distribution of each rotatable bond, combining dial (radial) plots with histograms and, when available, torsional potential curves (Figure 16, bottom). Comparing the torsion histograms with the underlying torsional potentials provides insight into whether the bound state forces the ligand into energetically strained conformations or whether it occupies low-energy rotamers, thereby informing opportunities to rigidify or re-optimize specific torsional degrees of freedom in future design cycles.

**Figure 17:**
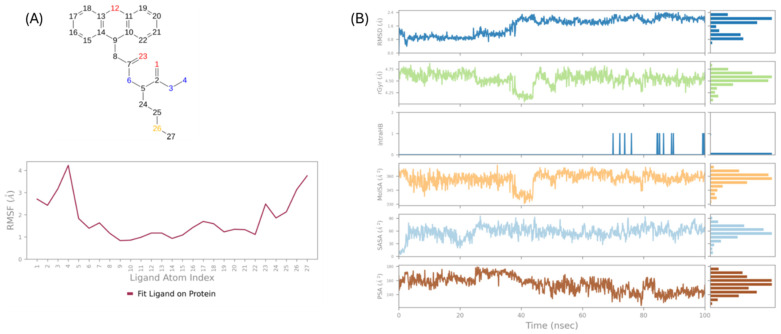
Compound **7h** (**A**) ligand root mean square fluctuation. (**B**) Ligand properties: From the top graph: Ligand RMSD: Root mean square deviation of a ligand with respect to the reference conformation; radius of gyration (rGyr); intramolecular hydrogen bonds (intraHB): number of internal hydrogen bonds (HB) within a ligand molecule; molecular surface area (MolSA): molecular surface calculation with 1.4 Å probe radius; solvent accessible surface area (SASA); polar surface area (PSA).

The ligand RMSD fluctuated between 3 and 4 Å, higher than the protein RMSD, reflecting conformational adjustments within the binding pocket rather than dissociation (Figure 17A). Atom-level RMSF analysis highlighted hydrazide and methylthio substituents as the most mobile, suggesting entropic contributions to binding, as revealed in Figure 17A.

Global ligand properties were monitored as a function of time, including ligand RMSD relative to the reference conformation, radius of gyration (rGyr), the number of intramolecular hydrogen bonds (intraHB), molecular surface area (MolSA), solvent accessible surface area (SASA), and polar surface area (PSA) (Figure 17B). The radius of gyration reports on the overall “extendedness” or compactness of the ligand, while intra-ligand hydrogen bonds can stabilize folded conformations and modulate the conformational entropy penalty upon binding.

MolSA, SASA, and PSA jointly describe how much of the ligand is buried versus solvent-exposed and how the polar component of the surface evolves during the trajectory, which is relevant for understanding desolvation, hydrogen-bonding capacity, and potential differences between bound and unbound solvation shells. Tracking these descriptors over 100 ns therefore complements the RMSD, RMSF, and contact analyses by quantifying how the ligand’s shape and solvation environment respond to the constraints of the binding pocket.

Taken together, the RMSD and RMSF analyses, secondary-structure monitoring, interaction timelines, and ligand-centered descriptors provide a coherent dynamic picture that complements the static docking poses and supports the suitability of the docked complex for further optimization. The results document a well-structured, predominantly α-helical protein, recurrent protein–ligand contacts persisting for a substantial fraction of the 100 ns trajectory, and a ligand whose flexibility and surface properties remain compatible with sustained engagement of the binding pocket.

## 3. Discussion

Ion channel inhibitors are gaining increasing attention across a wide range of tumor types. In this context, we designed and evaluated a series of xanthene-based derivatives targeting the TASK-1 (K2P3.1) channel to explore their potential anticancer activity [29,30,31]. The involvement of potassium channels in tumor proliferation and survival has been increasingly recognized [3]. Multiple potassium channel families are implicated in cancer progression, influencing cell proliferation, apoptosis, migration, invasion, and cell cycle regulation across a wide range of tumor types [32]. Voltage-gated potassium channels (Kv family), including Kv1.1, Kv1.3, Kv1.5, Kv7.1, and Kv11.1 (hERG), are frequently associated with tumor cell proliferation, apoptosis resistance, and metastatic behavior in cancers such as colorectal, breast, lung, prostate, pancreatic, and glioblastoma [3]. Calcium-activated potassium channels (KCa family) contribute primarily to cell proliferation and migration, particularly in prostate, breast, pancreatic, melanoma, and glial tumors. Inward rectifier potassium channels (Kir family) regulate proliferation, invasion, apoptosis, and cell cycle progression in small-cell lung cancer, gastric cancer, hepatocellular carcinoma, glioma, and breast cancer [3]. Two-pore domain potassium channels (K2P family) including TASK-1 (KCNK3), TASK-2, TASK-3, TREK-1, TREK-2, and TWIK channels are increasingly recognized as regulators of tumor growth, invasion, and apoptotic signaling in melanoma, colorectal cancer, lung cancer, pancreatic ductal adenocarcinoma, osteosarcoma, and ovarian cancer [3]. However, relatively few small-molecule modulators actually moved from bench to bedside in the treatment of colorectal cancer due to complex interplay of signaling networks [33].

A thorough search of SciFinder, Reaxys, and the primary literature revealed no prior reports of N-alkyl-2-(9H-xanthen-9-yl)acetamides, methyl 2-(2-(9H-xanthen-9-yl)acetamido)alkanoates, or 2-(2-(9H-xanthen-9-yl)acetamido)alkanhydrazides bearing the present substitution patterns at C-9, indicating that all compounds are structurally novel. Our design strategy was to exploit the well-validated xanthene pharmacophore as a planar, lipophilic scaffold for interaction with macromolecular targets such as K2P potassium channels, while systematically modulating the physicochemical properties and hydrogen-bonding capacity of the appended side chain at the benzylic C-9 position. To this end, we constructed three congeneric series: (i) N-alkyl-2-(9H-xanthen-9-yl)acetamides **5a–i**, bearing neutral or cationic aliphatic/heterocyclic amines with graded lipophilicity; (ii) methyl 2-(2-(9H-xanthen-9-yl)acetamido)alkanoates **6a–h**, incorporating α-amino-acid-derived side chains to introduce stereochemistry and polar/ionizable groups; and (iii) the corresponding 2-(2-(9H-xanthen-9-yl)acetamido)alkanhydrazides **7a–h**, designed to increase hydrogen-bond donor capacity and aqueous solubility while preserving the side-chain topology. This modular design around a single, pharmacologically relevant position enabled the creation of a focused library suitable for preliminary structure–activity relationship (SAR) exploration against HCT-116 colorectal cancer cells and for probing the compatibility of these substituents with binding to the TASK-1 (KCNK3) channel in silico.

Within the hydrazide series, compounds **7b**, **7f**, and **7h** all displayed micromolar cytotoxicity on HCT-116 cells, with IC_50_ values of 66.97, 78.05, and 72.48 µM, respectively, whereas **7g** was markedly less active (IC_50_ = 210.77 µM). Thus, 7b is marginally the most potent in terms of IC_50_, but 7h combines good antiproliferative activity with a clearly superior docking profile and binding mode. While docking provides a static representation of ligand binding, it does not account for dynamic stability under physiological conditions. To further validate the stability of the predicted binding mode, molecular dynamics (MD) simulations of the **7h**–TASK-1 complex were performed over 100 ns. The protein–ligand complex remained structurally stable, with backbone RMSD values within the expected range for an equilibrated system. The ligand RMSD, although moderately higher, reflected conformational adaptation within the binding pocket rather than dissociation. Importantly, persistent interactions were observed, including a stable hydrogen bond and water-bridge interaction with Asn240, maintained for a significant portion of the trajectory. RMSF analysis showed limited fluctuations in binding-site residues, supporting a stable interaction environment. Furthermore, secondary structure analysis confirmed preservation of the predominantly α-helical architecture of the TASK-1 channel. Collectively, these findings provide dynamic validation of the docking results and support the selection of compound **7h** as a promising lead candidate.

In TASK-1, **7h** engages both protein chains and establishes an extended H-bonding network through its amide carbonyl and hydrazide NH with Thr93 and Thr199 in both chains, in addition to extensive van der Waals contacts provided by the xanthene core and the methionine-derived side chain. This dual-chain binding and additional H-bonding are not observed to the same extent for the other active hydrazides and rationalize the selection of **7h** as the most promising lead despite only modest differences in IC_50_. From a physicochemical perspective, **7h** presents a balanced amphiphilic profile: the xanthene core and methionine thioether confer sufficient lipophilicity and conformational flexibility to traverse the cell membrane and to occupy the narrow hydrophobic cleft of TASK-1, whereas the amide and hydrazide groups provide polar contacts required for productive binding. In contrast, **7g**, which bears a more polar dicarboxamide backbone derived from glutamic acid, sacrifices cell permeability and shows markedly weaker cellular activity despite a reasonably favorable docking score. Overall, 7h achieves a better compromise between binding affinity, binding geometry, and cell-level properties than the other congeners, justifying its prioritization for mechanistic and imaging studies.

Comparison of the amino-acid ester series (**6a–h**) with their corresponding hydrazides (**7a–h**) highlights the importance of the terminal hydrazide motif for tuning both polarity and binding interactions. For example, conversion of the β-alanine ester **6b** (IC_50_ = 105.13 µM) into the corresponding hydrazide **7b** (IC_50_ = 66.97 µM) leads to ~1.5-fold enhancement in potency. An even more pronounced effect is observed for the methionine pair: the ester **6h** is the least active compound in the set (IC_50_ = 353.75 µM), whereas its hydrazide analogue **7h** exhibits a five-fold improvement in activity (IC_50_ = 72.48 µM). In contrast, for the aspartate-derived pair, 6f (IC_50_ = 75.35 µM) and 7f (IC_50_ = 78.05 µM) display comparable activity, indicating that the hydrazide effect is context dependent and modulated by the nature of the side chain.

At the binding site level, the hydrazide introduces an additional H-bond donor and acceptor and increases the conformational reach of the terminal chain. Docking of **7h** shows that the hydrazide participates in a potential polar contact with key polar residues (Thr93 from chain B and Thr199) while maintaining the xanthene ring in the hydrophobic pocket defined by Leu232, Ile235, Gly236, and Leu239. This polydentate polar interaction pattern cannot be fully replicated by the corresponding esters, which present fewer polar contacts and a more sterically encumbered terminal carbonyl. Consequently, the hydrazide group appears to act as a polar anchor that enhances productive engagement of the TASK-1 channel in those scaffolds where the side chain offers appropriate geometry and flexibility, as illustrated most clearly by the **6h/7h** pair.

In the N-alkyl amide series (**5a–i**), progressive modulation of the N-alkyl substituent reveals a significant contribution of lipophilicity and chain length. Short and medium linear chains such as in **5a** (n-propyl, IC_50_ = 103.28 µM) and **5b** (n-butyl, IC_50_ = 242.78 µM) give moderate to weak antiproliferative effects, whereas introduction of a much longer tetradecyl chain in **5g** significantly improves activity (IC_50_ = 77.56 µM). This gain in potency is mirrored at the computational level: **5g** exhibits the most favorable docking metrics in the series (XP score = −11.114 kcal/mol; MM-GBSA = −63.17 kcal/mol), exceeding even the crystal ligand, suggesting that its extended hydrophobic tail efficiently occupies the deep lipophilic cavity of the TASK-1 channel.

However, the improvement is not strictly monotonic with increasing lipophilicity, as illustrated by the relatively weak **5b**. This indicates that both the overall hydrophobic character and the topological reach of the side chain must be considered. The tetradecyl chain of **5g** is long and flexible enough to traverse the narrow access channel and to make extensive hydrophobic contacts deep inside the binding pocket, thereby stabilizing the ligand–channel complex. In contrast, intermediate chain lengths may position the terminal alkyl group in suboptimal regions, leading to poorer complementarity and weaker activity. At the cellular level, very high lipophilicity also carries the risk of reduced aqueous solubility and potential aggregation; thus, **5g** may be approaching the upper limit of beneficial lipophilicity in this chemotype, providing strong binding without completely compromising solubility on the time scale of the assay.

The leucine-derived ester 6e is unique in being completely inactive in the MTT assay (NI), despite belonging to an otherwise active amino-acid ester series in which 6c, 6d, 6f, and **6g** all show IC_50_ values below 100 µM. Interestingly, docking predicts a relatively favorable binding profile for **6e** (XP score = −8.716 kcal/mol; MM-GBSA = −29.28 kcal/mol), comparable to several active analogues. This discrepancy suggests that the loss of cellular activity is more likely related to physicochemical or conformational factors affecting target engagement in the cellular context rather than to intrinsic inability to interact with the TASK-1 binding site.

Structurally, **6e** carries a branched isobutyl side chain at the α-carbon (L-leucine), together with a terminal methyl ester. This combination may have several unfavorable consequences: (i) the bulky β-branched group can introduce steric clashes within the narrow access channel of TASK-1 and restrict the conformational freedom required to adopt the optimal bound pose predicted in silico; (ii) the relatively high hydrophobicity and compact shape of the leucine side chain, coupled with the neutral ester terminus, may promote non-specific membrane partitioning or aggregation rather than productive channel binding; and (iii) the ester is not expected to be efficiently hydrolyzed under the assay conditions to reveal a more polar, channel-compatible carboxylate, unlike endogenous peptide substrates. Taken together, these factors provide a plausible explanation for the lack of measurable cytotoxicity of 6e despite its reasonable docking score.

Comparison of the experimental IC_50_ values with the calculated XP scores and MM-GBSA binding energies indicates only a weak qualitative correlation between predicted binding affinity at TASK-1 and antiproliferative potency on HCT-116 cells. As noted above, **5g** shows the most favorable docking metrics (XP = −11.114 kcal/mol, MM-GBSA = −63.17 kcal/mol) but exhibits only moderate cellular potency (IC_50_ = 77.56 µM), comparable to several compounds with considerably less favorable scores, such as 6d (IC_50_ = 79.38 µM; XP = −7.963 kcal/mol) and **6f** (IC_50_ = 75.35 µM; XP = −8.928 kcal/mol). Conversely, **6h** has a reasonably good, predicted binding (XP = −8.811 kcal/mol; MM-GBSA = −32.45 kcal/mol) yet is the least active compound in the biological assay (IC_50_ = 353.75 µM). The methionine hydrazide **7h** stands out as a case where favorable docking (XP = −9.349 kcal/mol; MM-GBSA = −35.75 kcal/mol) coincides with good cytotoxicity (IC_50_ = 72.48 µM), but this is not a general rule across the set.

Quantitative image analysis of both nuclei and whole cells demonstrates that compound **7h** significantly suppresses HCT-116 cell proliferation, as reflected by the marked reduction in nuclear count, total cell number, and area coverage. In addition, decreased nuclear and cellular size, together with chromatin condensation, support the induction of apoptotic cell death. Collectively, these findings suggest that **7h** exerts its anticancer activity through combined inhibition of cell proliferation and promotion of apoptosis. Although docking indicates a shared binding mode of the active derivatives within the TASK-1 pocket, the correlation between predicted binding affinity and IC_50_ values is limited. Variations in cellular potency are likely influenced by factors such as membrane permeability, solubility, and intracellular stability. Therefore, the computational results should be regarded as supportive evidence of TASK-1 engagement rather than quantitative predictors of antiproliferative activity.

Despite the encouraging findings, several limitations should be acknowledged. The proposed involvement of TASK-1 inhibition is supported by molecular docking and further reinforced by molecular dynamics (MD) simulations, which demonstrated stable protein–ligand interactions over the simulation period; however, these findings still lack direct electrophysiological validation. Moreover, apoptosis assessment was primarily based on DAPI staining and morphological analysis; incorporation of additional biochemical assays, such as Annexin V/PI staining or caspase activation studies, would provide stronger mechanistic evidence. Future investigations will therefore focus on functional validation of TASK-1 channel inhibition, assessment of selectivity across other K2P family members, and evaluation of cytotoxic selectivity in normal colon epithelial cells. Collectively, the present work establishes xanthene-based hydrazide derivatives as a structurally tractable platform for potassium channel-targeted anticancer design, providing a solid foundation for the development of next-generation TASK-1-directed therapeutics.

## 4. Materials and Methods

### 4.1. Chemical Synthesis

General procedures. The solvents were dried and purified as normal. The petroleum ether utilized had a boiling point between 40 and 60 degrees Celsius. Thin layer chromatography (TLC): UV absorption is used to identify silica gel 60 F254 plastic plates (E. Merck, layer thickness 0.2 mm). Elemental analyses were carried out using Flash EA-1112 equipment. A Buchi 510 melting-point device was used to determine the melting points, and the results are uncorrected. Using tetramethylsilane as an internal standard, ^1^H and ^13^C NMR spectra were acquired at 400 MHz and 100 MHz, respectively, using a Bruker AC 400 in CDCl_3_ and DMSO solution (see Supplementary Materials). The preparation of methyl 2-(9H-xanthen-9-yl)acetate (2) followed published procedures [34].

Preparation of 2-(9H-xanthen-9-yl)acetohydrazide (3).

Ethanol 95% (20 mL) was used to dissolve a combination of methyl 2-(9H-xanthen-9-yl)acetate (2) (2.54 g, 10.0 mmol) and hydrazine hydrate (1.0 mL, 20 mmol), which was then refluxed for 10 h at 78 °C (TLC monitored). After cooling the reaction mixture, the crystals that formed were filtered out. Pure hydrazide 3 was created by crystallizing the obtained material with ethanol. white crystals, yield 84%. mp 159–161 °C. 1H NMR spectrum, (400 MHz, CDCl_3_), 1H NMR spectrum, (400 MHz, CDCl_3_), δ, ppm (J, Hz): 2.71 (d, J = 9.4 Hz, 2 H, CH_2_), 3.94 (bs, 2H, NH_2_), 4.23 (t, J = 9.4 Hz, 1 H, CH), 6.92–7.07 (m, 4 H, Ar-H), 7.22–7.29 (m, 2 H, Ar-H), 7.44–7.51 (m, 2 H, Ar-H), 9.13 (bs, 1H, NH). 13C-NMR (100.0 MHz, CDCl_3_), δ, ppm: 37.1 (CH2), 39.4 (CH), 116.7, 123.5, 124.1, 128.7, 129.2, 129.5, 133.8, 149.4, 151.6 (Ar-C), 171.3 (CO). MS (MALDI, positive mode, matrix DHB) *m*/*z*: 277.3 (M + Na)+. Anal. Calcd. For C_15_H_14_N_2_O_2_ (254.30) C, 70.85; H, 5.55; N, 11.02. Found: C, 71.19; H, 5.68 N, 11.36.

Preparation of N-Alkyl-2-(9H-xanthen-9-yl)acetamides 5a–i.

In an ice bath at −5 °C, a solution of 2-(9H-Xanthen-9-yl)acetohydrazide (3) (0.25 g 1.0 mmol) in 3 mL 1 N HCl and 1 mL acetic acid was continuously stirred. A portion-wise, continuously swirling cold sodium nitrite solution (0.1 g, 1.5 mmol) in 2 mL of water was added to this mixture. To obtain the in situ produced ethyl acetate solution of the corresponding azide 4, the reaction mixture was further agitated at −5 °C for one hour, extracted with ethyl acetate, and then washed with sodium carbonate (10 mL, 1 N), HCl (10 mL, 1 N), and water (10 mL). Finally, it was dried over sodium sulfate. After quickly adding cold ethyl acetate azide solution 4 to a cold ethyl acetate solution of amines, including propyl, butyl, isopropyl, allyl, benzyl, cyclohexyl, morpholine, piperidine, pyrrolidine, and β-naphthylethylenediamine, the mixture was placed in the freezer for 12 h. After another 12 h at ambient temperature, the ethyl acetate solution was washed with sodium carbonate, 1N HCl, and water and dried over sodium sulfate. The ethyl acetate solution was filtered, evaporated, and crystallized from the ethyl acetate pet ether combination to afford N-Alkyl-2-(9H-xanthen-9-yl)acetamides 5a–i in good yields.

N-Propyl-2-(9H-xanthen-9-yl)acetamide (**5a**).

From n-propylamine and the azide 6. white crystals, yield 74%. mp 101–102 °C. 1H NMR spectrum, (400 MHz, CDCl_3_), δ, ppm (J, Hz): 0.80 (t, J = 7.5 Hz, 3 H, CH_3_), 1.33–1.44 (m, 2 H, CH_2_), 2.46 (d, J = 6.0 Hz, 2 H, CH_2_CO), 3.08–3.15 (m, 2 H, NCH_2_), 4.57–4.63 (m, 1 H, CH), 5.21 (bs, 1 H, NH), 7.03–7.12 (m, 5 H, Ar-H), 7.21–7.32 (m, 3 H, Ar-H). 13C-NMR (100.0 MHz, CDCl_3_), δ, ppm: 11.3 (CH_3_), 22.6 (CH_2_), 36.3 (CH_2_CO), 41.3 (NHCH_2_), 48.5 (CH), 116.4 (Cq), 116.5 (CHAr), 123.4 (Cq), 123.5 (CHAr), 124.5 (Cq), 128.0 (CHAr), 128.8 (CHAr), 152.0 (Cq), 170.3 (C=O). MS (MALDI, positive mode, matrix DHB) *m*/*z*: 304.3 (M + Na)+. Anal. Calcd. For C_18_H_19_NO_2_ (281.36) C, 76.84; H, 6.81; N, 4.98. Found: C, 76.54; H, 6.97; N, 5.23.

N-Butyl-2-(9H-xanthen-9-yl)acetamide (**5b**).

From n-butylamine and the azide 4. white crystals, yield 66%. mp 111–112 °C. 1H NMR spectrum, (400 MHz, CDCl_3_), δ, ppm (J, Hz): 0.85–0.91 (m, 3 H, CH_3_), 1.18–1.39 (m, 4 H, 2CH_2_), 2.46 (d, J = 6.0 Hz, 2 H, CH_2_CO), 3.13–3.19 (m, 2 H, NCH_2_), 4.57–4.62 (m, 1 H, CH), 5.15 (bs, 1 H, NH), 7.04–7.13 (m, 5 H, Ar-H), 7.22–7.34 (m, 3 H, Ar-H). 13C-NMR (100.0 MHz, CDCl_3_), δ, ppm: 13.7 (CH_3_), 19.9 (CH_2_), 31.3 (CH_2_), 35.9 (CH_2_CO), 39.3 (NHCH_2_), 48.4 (CH), 116.5 (Cq), 116.6 (CHAr), 123.5 (Cq), 123.9 (CHAr), 124.5 (Cq), 128.0 (CHAr), 128.8 (CHAr), 152.1 (Cq), 170.4 (C=O). MS (MALDI, positive mode, matrix DHB) *m*/*z*: 318.3 (M + Na)+. Anal. Calcd. For C_19_H_21_NO_2_ (295.38) C, 77.26; H, 7.17; N, 4.74. Found: C, 77.01; H, 7.43; N, 4.92.

N-Isopropyl-2-(9H-xanthen-9-yl)acetamide (**5c**).

From isopropylamine and the azide 4. white crystals, yield 68%. mp 89–90 °C. 1H NMR spectrum, (400 MHz, CDCl_3_), δ, ppm (J, Hz): 1.01 (d, J= 6.0 Hz, 6 H, 2CH_3_), 2.43 (d, J = 6.0 Hz, 2 H, CH_2_CO), 3.97–4.08 (m, 1 H, NCH), 4.57–4.64 (m, 1 H, CH), 4.98 (bs, 1 H, NH), 7.04–7.13 (m, 5 H, Ar-H), 7.21–7.36 (m, 3 H, Ar-H). 13C-NMR (100.0 MHz, CDCl_3_), δ, ppm: 22.5 (2CH_3_), 36.4 (CH_2_CO), 41.5 (NHCH), 48.4 (CH), 116.5 (Cq), 116.6 (CHAr), 123.4 (Cq), 123.6 (CHAr), 124.5 (Cq), 128.0 (CHAr), 128.8 (CHAr), 152.0 (Cq), 169.5 (C=O). MS (MALDI, positive mode, matrix DHB) *m*/*z*: 304.3 (M + Na)+. Anal. Calcd. For C18H19NO2 (281.36) C, 76.84; H, 6.81; N, 4.98. Found: C, 77.12; H, 6.54; N, 5.14.

N-Allyl-2-(9H-xanthen-9-yl)acetamide (**5d**).

From allyl amine and the azide 4. white crystals, yield 61%. mp 77–79 °C. 1H NMR spectrum, (400 MHz, CDCl_3_), δ, ppm (J, Hz): 2.50 (d, J = 6.0 Hz, 2 H, CH_2_CO), 3.78–3.83 (m, 1 H, NCH_2_), 4.62 (t, J = 6.0 Hz, 1 H, CH), 4.98–5.08 (m, 2 H, CH=CH_2_), 5.21 (bs, 1 H, NH), 5.63–5.76 (m, 1 H, CH=CH_2_), 7.05–7.07 (m, 4 H, Ar-H), 7.22–7.33 (m, 4 H, Ar-H). 13C-NMR (100.0 MHz, CDCl_3_), δ, ppm: 36.3 (CH_2_CO), 41.9 (NHCH_2_), 48.3 (CH), 116.5 (Cq), 116.6 (CHAr), 116.7 (CH=CH_2_), 123.4 (Cq), 123.6 (CHAr), 124.6 (Cq), 128.0 (CHAr), 128.8 (CHAr), 133.6 (CH=CH_2_), 152.0 (Cq), 170.2 (C=O). MS (MALDI, positive mode, matrix DHB) *m*/*z*: 302.3 (M + Na)+. Anal. Calcd. For C_18_H_17_NO_2_ (279.34) C, 77.40; H, 6.13; N, 5.01. Found: C, 77.13; H, 6.41; N, 4.87.

N-Benzyl-2-(9H-xanthen-9-yl)acetamide (**5e**).

From benzyl amine and the azide 4. white crystals, yield 88%. mp 129–130 °C. 1H NMR spectrum, (400 MHz, CDCl_3_), δ, ppm (J, Hz): 2.52 (d, J= 6.0 Hz, 2 H, CH_2_CO), 4.35 (d, J= 6.0 Hz, 2 H, NHCH_2_), 4.58–4.67 (m, 1 H, CH), 5.48 (bs, 1 H, NH), 7.04–7.13 (m, 5 H, Ar-H), 7.21–7.36 (m, 8 H, Ar-H). 13C-NMR (100.0 MHz, CDCl_3_), δ, ppm: 36.2 (CH_2_CO), 43.7 (NHCH_2_), 48.3 (CH), 116.4 (Cq), 116.5 (CHAr), 123.5 (Cq), 123.6 (CHAr), 124.6 (Cq), 127.5 (Cq), 127.8 (CHAr), 128.1 (CHAr), 128.6 (CHAr), 128.8 (CHAr), 137.7 (CHAr), 152.1 (Cq), 170.2 (C=O). MS (MALDI, positive mode, matrix DHB) *m*/*z*: 352.3 (M + Na)+. Anal. Calcd. For C_22_H_19_NO_2_ (329.40) C, 80.22; H, 5.81; N, 4.25. Found: C, 80.57; H, 6.12; N, 4.05.

1-(Piperidin-1-yl)-2-(9H-xanthen-9-yl)ethan-1-one (**5f**).

From piperidine and azide 4. white crystals, yield 69%. mp 141–142 °C. 1H NMR spectrum, (400 MHz, CDCl_3_), δ, ppm (J, Hz): 1.37–1.54 (m, 6 H, 3CH_2_), 2.50 (d, J = 6.0 Hz, 2 H, CH_2_CO), 3.43–3.46 (m, 4 H, 2NCH_2_), 4.58–4.67 (m, 1 H, CH), 7.05–7.11 (m, 5 H, Ar-H), 7.22–7.27 (m, 3 H, Ar-H). 13C-NMR (100.0 MHz, CDCl_3_), δ, ppm: 22.6 (CH2), 22.8 (CH_2_), 23.9 (CH_2_), 36.6 (CH_2_CO), 42.8 (NCH2), 44.2 (NCH_2_), 46.8 (CH), 116.4 (Cq), 116.5 (CHAr), 123.4 (Cq), 123.5 (CHAr), 124.4 (Cq), 127.9 (CHAr), 128.8 (CHAr), 152.3 (Cq), 168.8 (C=O). MS (MALDI, positive mode, matrix DHB) *m*/*z*: 330.3 (M + Na)+. Anal. Calcd. For C_20_H_21_NO_2_ (307.4) C, 78.15; H, 6.89; N, 4.56. Found: C, 77.87; H, 7.12; N, 4.32.

N-Tetradecyl-2-(9H-xanthen-9-yl)acetamide (**5g**).

From tetradecyl amine and the azide 4. white crystals, yield 59%. mp 59–60 °C. 1H NMR spectrum, (400 MHz, CDCl_3_), δ, ppm (J, Hz): 086–091 (m, 3 H, CH_3_), 1.25–1.33 (m, 24 H, 12CH_2_), 2.47 (d, J = 6.0 Hz, 2 H, CH_2_CO), 3.26–3.31 (m, 2H, NCH_2_), 4.58 (t, J = 6.0 Hz, 1 H, CH), 5.22 (bs, 1H, NH), 7.03–7.12 (m, 4 H, Ar-H), 7.21–7.34 (m, 4 H, Ar-H). 13C-NMR (100.0 MHz, CDCl_3_), δ, ppm: 14.1 (CH_3_), 22.7 (CH_2_), 26.7 (CH_2_), 26.8 (CH_2_), `36.2 (CH_2_CO), 39.6 (NHCH_2_), 48.5 (CH), 116.4 (Cq), 116.5 (CHAr), 123.4 (Cq), 123.6 (CHAr), 124.7 (Cq), 128.0 (CHAr), 128.8 (CHAr), 152.0 (Cq), 170.1 (C=O). MS (MALDI, positive mode, matrix DHB) *m*/*z*: 458.6 (M + Na)+. Anal. Calcd. For C_29_H_41_NO_2_ (435.65) C, 79.95; H, 9.49; N, 3.22. Found: C, 80.23; H, 9.53; N, 3.43.

N-(2-(Naphthalen-2-ylamino)ethyl)-2-(9H-xanthen-9-yl)acetamide (**5h**).

From β-naphthylethylenediamine and the azide 4. white crystals, yield 67%. mp 109–110 °C. 1H NMR spectrum, (400 MHz, CDCl_3_), δ, ppm (J, Hz): 2.55 (d, J= 4.6 Hz, 2 H, CH_2_), 3.26–3.29 (m, 2 H, CH_2_), 3.50–3.53 (m, 2 H, CH_2_), 4.57–4.63 (m, 1 H, CH), 5.17 (bs, 1 H, NH), 5.39 (bs, 1 H, NH), 7.14–7.30 (m, 4 H, Ar-H), 7.33–7.37 (m, 5 H, Ar-H), 7.54–7.69 (m, 4 H, Ar-H), 7.72–7.89 (m, 4 H, Ar-H). 13C-NMR (100.0 MHz, CDCl_3_), δ, ppm: 36.4 (CH), 38.7 (CH_2_), 47.4 (CH2), 48.2 (CH_2_), 116.6, 120.4, 123.4, 123.6, 123.8, 124.4, 124.5, 125.6, 126.2, 126.3, 128.0, 128.1, 128.6, 128.7, 134.3, 152.1 (C-Ar), 171.7 (CO). MS (MALDI, positive mode, matrix DHB) *m*/*z*: 431.5 (M + Na)+. Anal. Calcd. For C_27_H_24_N_2_O_2_ (408.50) C, 79.39; H, 5.92; N, 6.86. Found: C, 79.62; H, 6.21; N, 6.59.

1-Morpholino-2-(9H-xanthen-9-yl)ethan-1-one (**5i**).

From morpholine and the azide 4. white crystals, yield 81%. mp 117–119 °C. 1H NMR spectrum, (400 MHz, CDCl_3_), δ, ppm (J, Hz): 2.55 (d, J = 6.0 Hz, 2 H, CH_2_CO), 3.47–3.57 (m, 4 H, 2 NCH_2_), 3.68–3.71 (m, 4 H, 2 OCH_2_), 4.58–4.63 (m, 1 H, CH), 7.05–7.14 (m, 5 H, Ar-H), 7.22–7.34 (m, 3 H, Ar-H). 13C-NMR (100.0 MHz, CDCl_3_), δ, ppm: 35.9 (CH_2_CO), 47.4 (NCH2), 48.2 (NCH_2_), 62.5 (2OCH_2_), 116.4, 116.6, 123.4, 123.6, 124.4, 128.0, 128.7, 152.1 (C-Ar), 173.0 (C=O). MS (MALDI, positive mode, matrix DHB) *m*/*z*: 332.3 (M + Na)+. Anal. Calcd. For C_19_H_19_NO_3_ (309.36) C, 73.77; H, 6.19; N, 4.53. Found: C, 73.82; H, 6.52; N, 4.87.

Preparation of methyl 2-(2-(9H-xanthen-9-yl)acetamido) alkanoates 6a–h.

Using 2-(9H-xanthen-9-yl)acetohydrazide (3), amino acid methyl ester hydrochloride, glycine, *β*-alanine, *L*-alanine, *L*-serine. *L*-leucine, *L*-aspartic acid, *L*-glutamic acid, and *L*-methionine in the presence of triethylamine, the azide coupling process was described above.

Methyl 2-(2-(9H-xanthen-9-yl)acetamido) acetate (**6a**).

From glycine and the azide 4. white crystals, yield 85%. mp 129–130 °C. 1H NMR spectrum, (400 MHz, CDCl_3_), δ, ppm (J, Hz): 2.57 (d, J = 6.0 Hz, 2 H, CH_2_CO), 3.73 (s, 1 H, OCH_3_), 3.98 (d, J = 6.0 Hz, 2 H, NHCH_2_), 4.61 (t, J = 6.0 Hz, 1 H, CH), 5.69 (bs, 1 H, NH), 7.03–7.12 (m, 5 H, Ar-H), 7.21–7.34 (m, 3 H, Ar-H). 13C-NMR (100.0 MHz, CDCl3), δ, ppm: 36.0 (CH_2_CO), 41.3 (NHCH_2_), 47.9 (CH), 52.4 (OCH_3_), 116.4 (Cq), 116.8 (CHAr), 123.4 (Cq), 123.6 (CHAr), 124.5 (Cq), 128.1 (CHAr), 128.8 (CHAr), 152.0 (Cq), 170.0 (C=O), 170.4 (C=O). MS (MALDI, positive mode, matrix DHB) *m*/*z*: 334.3 (M + Na)+. Anal. Calcd. For C_18_H_17_NO_4_ (311.34) C, 69.44; H, 5.50; N, 4.50. Found: C, 69.68; H, 5.35; N, 4.83.

Methyl 3-(2-(9H-xanthen-9-yl)acetamido) propanoate (**6b**).

From *β*-alanine and the azide 4. white crystals, yield 79%. mp 95–96 °C. 1H NMR spectrum, (400 MHz, CDCl_3_), δ, ppm (J, Hz): 2.42–2.47 (m, 4 H, 2CH_2_CO), 3.39–3.45 (m, 2 H, NHCH_2_), 3.64 (s, 3 H, OCH_3_), 4.54–4.62 (m, 1 H, CH), 5.71 (bs, 1 H, NH), 7.02–7.05 (m, 4 H, Ar-H), 7.20–7.31 (m, 4 H, Ar-H). 13C-NMR (100.0 MHz, CDCl_3_), δ, ppm: 33.6 (CH_2_CO), 34.6 (NHCH_2_), 36.3 (CH_2_CO), 48.2 (CH), 51.7 (OCH_3_), 116.4 (Cq), 116.5 (CHAr), 123.4 (Cq), 123.6 (CHAr), 124.6 (Cq), 128.0 (CHAr), 128.8 (CHAr), 152.1 (Cq), 170.3 (C=O), 172.7 (C=O). MS (MALDI, positive mode, matrix DHB) *m*/*z*: 348.3 (M + Na)+. Anal. Calcd. For C_19_H_19_NO_4_ (325.36) C, 70.14; H, 5.89; N, 4.31. Found: C, 70.47; H, 6.09; N, 4.05.

Methyl 2-(2-(9H-xanthen-9-yl)acetamido) propanoate (**6c**).

From *L*-alanine and the azide 4. white crystals, yield 73%. mp 87–88 °C. 1H NMR spectrum, (400 MHz, CDCl_3_), δ, ppm (J, Hz): 1.28 (d, J = 6.0 Hz, 3 H, CH_3_), 2.55 (d, J = 6.0 Hz, 2 H, CH_2_CO), 3.60 (s, 3 H, OCH_3_), 4.44–4.51 (m, 2 H, NHCH, CH), 5.31 (bs, 1 H, NH), 7.09–7.13 (m, 5 H, Ar-H), 7.22–7.36 (m, 3 H, Ar-H). 13C-NMR (100.0 MHz, CDCl_3_), δ, ppm: 17.2 (CH_3_), 35.9 (CH_2_CO), 47.4 (CH), 49.0 (NHCH), 52.4 (OCH_3_), 116.5 (Cq), 116.6 (CHAr), 123.4 (Cq), 123.6 (CHAr), 124.5 (Cq), 128.1 (CHAr), 128.8 (CHAr), 152.1 (Cq), 172.7 (C=O), 172.9 (C=O). MS (MALDI, positive mode, matrix DHB) *m*/*z*: 348.3 (M + Na)+. Anal. Calcd. For C_19_H_19_NO_4_ (325.36) C, 70.14; H, 5.89; N, 4.31. Found: C, 70.57; H, 6.21; N, 4.65.

Methyl 3-hydroxy-2-(2-(9H-xanthen-9-yl)acetamido) propanoate (**6d**).

From *L*-serine and the azide 4. white crystals, yield 54%. mp 116–117 °C. 1H NMR spectrum, (400 MHz, CDCl_3_), δ, ppm (J, Hz): 2.53 (d, J = 6.0 Hz, 2 H, CH_2_CO), 3.06–3.15 (m, 2 H, OCH_2_), 3.73 (s, 3 H, OCH_3_), 3.79–3.89 (m, 1 H, CH), 4.56–4.61 (m, 1 H, CH), 5.31 (bs, 1 H, OH), 6.35 (bs, 1H, NH), 7.05–7.13 (m, 5 H, Ar-H), 7.22–7.34 (m, 3 H, Ar-H). 13C-NMR (100.0 MHz, CDCl_3_), δ, ppm: 35.8 (CH_2_CO), 46.2 (CH), 52.6 (OCH_3_), 54.7 (NHCH), 62.8 (OCH_2_), 116.5 (Cq), 116.6 (CHAr), 123.4 (Cq), 123.6 (CHAr), 124.5 (Cq), 128.1 (CHAr), 128.8 (CHAr), 152.0 (Cq), 170.7 (C=O), 173.4 (C=O). MS (MALDI, positive mode, matrix DHB) *m*/*z*: 364.3 (M + Na)+. Anal. Calcd. For C_19_H_19_NO_5_ (341.36) C, 66.85; H, 5.61; N, 4.10. Found: C, 67.03; H, 5.46; N, 4.34.

Methyl 4-methyl-2-(2-(9H-xanthen-9-yl)acetamido) pentanoate (**6e**).

From *L*-leucine and the azide 4. white crystals, yield 64%. mp 57–58 °C. 1H NMR spectrum, (400 MHz, CDCl_3_), δ, ppm (J, Hz): 0.86–0.91 (m, 6 H, 2CH_3_), 1.36–1.44 (m, H, CH), 2.45–2.62 (m, 4 H, CH2, CH_2_CO), 3.69 (s, 3 H, OCH_3_), 4.56–4.63 (m, 2 H, CH, CH), 5.64 (bs, 1 H, NH), 7.07–7.11 (m, 5 H, Ar-H), 7.20–7.31 (m, 3 H, Ar-H). 13C-NMR (100.0 MHz, CDCl_3_), δ, ppm: 22.7 (2CH_3_), 24.5 (CH), 30.3 (CH_2_CO), 35.9 (CH_2_), 47.3 (CH), 50.6 (NHCH), 52.3 (OCH_3_), 116.4 (Cq), 116.5 (CHAr), 123.3 (Cq), 123.5 (CHAr), 124.5 (Cq), 128.0 (CHAr), 128.8 (CHAr), 152.0 (Cq), 170.1 (C=O), 173.3 (C=O). MS (MALDI, positive mode, matrix DHB) *m*/*z*: 390.4 (M + Na)+. Anal. Calcd. For C_22_H_25_NO_4_ (367.45) C, 71.91; H, 6.86; N, 3.81. Found: C, 72.15; H, 6.91; N, 3.44.

Dimethyl 2-(2-(9H-xanthen-9-yl)acetamido) butan1,4-dioate (**6f**).

From *L*-aspartic acid and the azide 4. white crystals, yield 75%. mp 105–106 °C. 1H NMR spectrum, (400 MHz, CDCl_3_), δ, ppm (J, Hz): 2.48–2.64 (m, 4 H, 2CH_2_CO), 3.63 (s, 3 H, OCH_3_), 3.71 (s, 3 H, OCH_3_), 4.57–4.63 (m, 1 H, CH), 4.79–4.85 (m, 1 H, NHCH), 6.19 (bs, 1 H, NH), 7.02–7.07 (m, 4 H, Ar-H), 7.25–7.34 (m, 4 H, Ar-H). 13C-NMR (100.0 MHz, CDCl_3_), δ, ppm: 35.9 (CH_2_CO), 36.1 (CH_2_CO), 48.2 (CH), 51.9 (NHCH), 52.7 (OCH_3_), 54.9 (OCH_3_), 116.5 (Cq), 116.6 (CHAr), 123.4 (Cq), 123.6 (CHAr), 124.5 (Cq), 128.0 (CHAr), 128.8 (CHAr), 152.1 (Cq), 169.9 (C=O), 170.9 (CO), 171.3 (CO). MS (MALDI, positive mode, matrix DHB) *m*/*z*: 406.4 (M + Na)+. Anal. Calcd. For C_21_H_21_NO_6_ (383.40) C, 65.79; H, 5.52; N, 3.65. Found: C, 65.93; H, 5.2 N, 3.39.

Dimethyl 2-(2-(9H-xanthen-9-yl)acetamido) pentan-1,5-dioate (**6g**).

From *L*-glutamic acid and the azide 4. white crystals, yield 69%. mp 95–96 °C. 1H NMR spectrum, (400 MHz, CDCl_3_), δ, ppm (J, Hz): 2.11–2.23 (m, 2 H, CH_2_), 2.52–2.58 (m, 4 H, 2CH_2_CO), 3.67 (s, 3 H, OCH_3_), 3.71 (s, 3 H, OCH_3_), 4.57–4.62 (m, 2 H, CH, NHCH), 5.99 (bs, 1 H, NH), 7.04–7.13 (m, 4 H, Ar-H), 7.20–7.34 (m, 4 H, Ar-H). 13C-NMR (100.0 MHz, CDCl_3_), δ, ppm: 27.1 (CH2), 29.7 (CH_2_CO), 36.0 (CH_2_CO), 47.9 (CH), 51.5 (CH), 51.8 (OCH_3_), 52.5 (OCH_3_), 116.5 (Cq), 116.6 (CHAr), 123.4 (Cq), 123.6 (CHAr), 124.5 (Cq), 128.0 (CHAr), 128.7 (CHAr), 152.0 (Cq), 170.2 (C=O), 172.0 (C=O), 173.2 (C=O). MS (MALDI, positive mode, matrix DHB) *m*/*z*: 420.4 (M + Na)+. Anal. Calcd. For C_22_H_23_NO_6_ (397.43) C, 66.49; H, 5.83; N, 3.52. Found: C, 66.84; H, 5.56; N, 3.75.

Methyl 4-methylmercapto -2-(2-(9H-xanthen-9-yl)acetamido) butanoate (**6h**).

From *L*-methionine. white crystals, yield 58%. mp 71–72 °C. 1H NMR spectrum, (400 MHz, CDCl_3_), δ, ppm (J, Hz): 2.05 (s, 3 H, SCH_3_), 2.49–2.61 (m, 4 H, CH_2_CO, CH_2_), 3.06–3.16 (m, 2 H, CH_2_), 3.72–3.79 (m, 4 H, NCH, OCH_3_), 4.55–4.59 (m, 1 H, CH), 5.91 (bs, 1 H, NH), 7.04–7.13 (m, 5 H, Ar-H), 7.22–7.35 (m, 3 H, Ar-H). 13C-NMR (100.0 MHz, CDCl_3_), δ, ppm: 15.3 (SCH_3_), 29.7 (CH_2_), 31.4 (CH_2_), 35.9 (CH_2_CO), 47.4 (CH), 51.4 (CH), 52.5 (OCH_3_), 116.5 (Cq), 116.6 (CHAr), 123.4 (Cq), 123.6 (CHAr), 124.5 (Cq), 128.0 (CHAr), 128.8 (CHAr), 152.0 (Cq), 170.0 (C=O), 172.9 (C=O). MS (MALDI, positive mode, matrix DHB) *m*/*z*: 408.4 (M + Na)+. Anal. Calcd. For C_21_H_23_NO_4_S (385.48) C, 65.43; H, 6.01; N, 3.63. Found: C, 65.67; H, 5.85; N, 3.46.

2-(2-(9H-Xanthen-9-yl)acetamido) alkanhydrazides 7a–h.

Hydrazides 7a–h was prepared according to the described procedure above using the esters; methyl 2-(2-(9H-xanthen-9-yl)acetamido) alkanoates 6a–h, reaction time 6 h.

2-(2-(9H-Xanthen-9-yl)acetamido) ethanhydrazide (**7a**).

White crystals, yield 86%. mp 169–170 °C. 1H NMR spectrum, (400 MHz, DMSO d6), δ, ppm (J, Hz): 2.38 (d, J = 6.0 Hz, 2 H, CH_2_CO), 3.61 (d, J = 6.0 Hz, 2 H, NHCH_2_), 4.22 (bs, 2 H, NH_2_), 4.44–4.52 (m, 1 H, CH), 7.04–7.12 (m, 5 H, Ar-H), 7.22–7.34 (m, 3 H, Ar-H), 8.08 (bs, 1 H, NH), 8.69 (bs, 1H, NH). 13C-NMR (100.0 MHz, DMSO d6), δ, ppm: 35.2 (CH2CO), 42.7 (NHCH_2_), 47.3 (CH), 116.5 (Cq), 116.8 (CHAr), 123.9 (Cq), 125.2 (CHAr), 125.5 (Cq), 128.3 (CHAr), 128.4 (CHAr), 151.8 (Cq), 168.6 (C=O), 170.3 (C=O). MS (MALDI, positive mode, matrix DHB) *m*/*z*: 334.3 (M + Na)+. Anal. Calcd. For C_17_H_17_N_3_O_3_ (311.34) C, 65.58; H, 5.50; N, 13.50 Found: C, 65.22; H, 5.86; N, 13.24.

3-(2-(9H-Xanthen-9-yl)acetamido) propanhydrazide (**7b**).

White crystals, yield 78%. mp 126–127 °C. 1H NMR spectrum, (400 MHz, DMSO), δ, ppm (J, Hz): 2.11 (t, J = 9.4 Hz, 2 H, CH_2_CO), 2.38 (d, J = 6.4 Hz, 2 H, CH_2_), 3.15–3.22 (m, 2 H, NCH_2_), 4.13 (bs, 2 H, NH_2_), 4.47 (t, J = 6.4 Hz, 1 H, CH), 7.06–7.13 (m, 5 H, Ar-H), 7.22–7.35 (m, 3 H, Ar-H), 7.83 (bs, 1 H, NH), 8.99 (bs, 1 H, NH). 13C-NMR (100.0 MHz, DMSO), δ, ppm: 33.9 (CH_2_CO), 35.5 (CH_2_CO), 35.7 (NCH_2_), 47.7 (CH), 116.5 (Cq), 116.8 (CHAr), 123.9 (Cq), 125.2 (CHAr), 125.5 (Cq), 128.3 (CHAr), 129.3 (CHAr), 151.8 (Cq), 169.9 (C=O), 170.1 (C=O). MS (MALDI, positive mode, matrix DHB) *m*/*z*: 348.3 (M + Na)+. Anal. Calcd. For C_18_H_19_N_3_O_3_ (325.37) C, 66.45; H, 5.89; N, 12.91. Found: C, 66.17; H, 6.11; N, 13.09.

2-(2-(9H-Xanthen-9-yl)acetamido) propanhydrazide (**7c**).

White crystals, yield 72%. mp 111–112 °C. 1H NMR spectrum, (400 MHz, DMSO), δ, ppm (J, Hz): 1.03 (d, J = 6.0 Hz, 3 H, CH_3_), 2.44 (d, J = 7.2 Hz, 2 H, CH_2_CO), 3.97–4.08 (m, 1H, NCH), 4.22 (bs, 2H, NH_2_), 4.59–4.66 (m, 1 H, CH), 4.98 (bs, 1 H, NH), 7.07–7.15 (m, 5 H, Ar-H), 7.22–7.37 (m, 3 H, Ar-H), 9.28 (bs, 1H, NH). 13C-NMR (100.0 MHz, DMSO), δ, ppm: 17.2 (CH_3_), 35.9 (CH_2_CO), 47.4 (CH), 49.4 (NHCH), 116.5 (Cq), 116.6 (CHAr), 123.4 (Cq), 123.6 (CHAr), 124.5 (Cq), 128.1 (CHAr), 128.7 (CHAr), 152.1 (Cq), 166.9 (C=O), 171.4 (C=O). MS (MALDI, positive mode, matrix DHB) *m*/*z*: 348.3 (M + Na)+. Anal. Calcd. For C_18_H_19_N_3_O_3_ (325.37) C, 66.45; H, 5.89; N, 12.91. Found: C, 66.19; H, 6.12; N, 12.66.

3-Hydroxy-2-(2-(9H-xanthen-9-yl)acetamido) propanhydrazide (**7d**).

White crystals, yield 69%. mp 162–163 °C. 1H NMR spectrum, (400 MHz, DMSO), δ, ppm (J, Hz): 2.53 (d, J = 6.0 Hz, 2 H, CH_2_CO), 3.74–3.89 (m, 2 H, OCH_2_), 4.13 (bs, 2 H, NH_2_), 4.55–4.61 (m, 2 H, NCH, CH), 5.31 (bs, 1 H, OH), 6.35 (bs, 1H, NH), 7.06–7.12 (m, 5 H, Ar-H), 7.22–7.34 (m, 3 H, Ar-H), 9.21 (bs, 1H, NH). 13C-NMR (100.0 MHz, DMSO), δ, ppm: 36.1 (CH), 46.2 (CH_2_), 54.7 (NHCH), 62.8 (CH_2_OH), 116.5, 118.2, 123.5, 123.8, 124.6, 124.8, 126.6, 128.3, 128.6, 134.8, 152.2, 152.3 (C-Ar), 169.5, 170.8 (2 CO). MS (MALDI, positive mode, matrix DHB) *m*/*z*: 364.3 (M + Na)+. Anal. Calcd. For C_18_H_19_N_3_O_4_ (341.37). C, 63.33; H, 5.61; N, 12.31 Found: C, 63.76; H, 5.45; N, 12.24.

4-Methyl-2-(2-(9H-xanthen-9-yl)acetamido) pentanhydrazide (**7e**).

White crystals, yield 69%. mp 142–143 °C. 1H NMR spectrum, (400 MHz, DMSO), δ, ppm (J, Hz): 0.86–0.91 (m, 6 H, 2CH_3_), 1.35–1.54 (m, 3 H, CH, CH_2_), 2.53–2.55 (m, 2 H, CH_2_CO), 4.09 (bs, 2H, NH_2_), 4.56–4.64 (m, 2 H, NCH, CH), 5.65 (bs, 1 H, NH), 7.06–7.12 (m, 5 H, Ar-H), 7.21–7.32 (m, 3 H, Ar-H), 9.24 (bs, 1H, NH). 13C-NMR (100.0 MHz, DMSO), δ, ppm: 21.9 (2CH_3_), 24.1 (CH), 33.6 (CH_2_), 35.9 (CH_2_CO), 46.9 (CH), 50.6 (NHCH), 116.5 (Cq), 116.6 (CHAr), 123.6 (Cq), 124.5 (CHAr), 124.6 (Cq), 128.3 (CHAr), 128.7 (CHAr), 152.1 (Cq), 170.1 (C=O), 173.3 (C=O). MS (MALDI, positive mode, matrix DHB) *m*/*z*: 390.4 (M + Na)+. Anal. Calcd. For C_21_H_25_N_3_O_3_ (367.45) C, 68.64; H, 6.86; N, 11.44. Found: C, 68.37; H, 6.98; N, 11.27.

2-(2-(9H-xanthen-9-yl)acetamido) butan1,4-dihydrazide (**7f**).

White crystals, yield 78%. mp 176–177 °C. 1H NMR spectrum, (400 MHz, DMSO), δ, ppm (J, Hz): 2.50–2.61 (m, 4 H, 2CH_2_CO), 4.10 (bs, 2H, NH_2_), 4.17 (bs, 2H, NH_2_), 4.56–4.64 (m, 1 H, CH), 4.76–4.84 (m, 2 H, NCH), 6.21 (bs, 1 H, NH), 7.02–7.13 (m, 5 H, Ar-H), 7.22–7.36 (m, 3 H, Ar-H), 9.17 (bs, 1H, NH), 9.23 (bs, 1H, NH). 13C-NMR (100.0 MHz, DMSO), δ, ppm: 35.9 (CH_2_CO), 36.3 (CH_2_CO), 47.4 (CH), 48.2 (NHCH), 116.5 (Cq), 116.6 (CHAr), 123.2 (Cq), 123.6 (Cq), 124.3 (CHAr), 124.6 (Cq), 128.1 (CHAr), 128.6 (CHAr), 152.1 (Cq), 168.5 (C=O),168.8 (C=O), 169.9 (C=O). MS (MALDI, positive mode, matrix DHB) *m*/*z*: 406.4 (M + Na)+. Anal. Calcd. For C_19_H_21_N_5_O_4_ (383.41) C, 59.52; H, 5.52; N, 18.27. Found: C, 59.526; H, 5.85; N, 18.11.

2-(2-(9H-Xanthen-9-yl)acetamido) pentan-1,5-dihydrazide (**7g**).

White crystals, yield 76%. mp 187–188 °C. 1H NMR spectrum, (400 MHz, DMSO), δ, ppm (J, Hz): 1.59–1.69 (m, 2 H, CH_2_), 1.72–1.92 (m, 2 H, CH_2_CO), 2.38 (d, J = 7.2 Hz, 2 H, CH_2_CO), 4.16–4.50 (m, 2H, 2NH_2_, CH, CH), 7.03–7.13 (m, 5 H, Ar-H), 7.21–7.37 (m, 3 H, Ar-H), 8.07 (bs, 1 H, NH), 8.96 (bs, 1H, NH), 9.18 (bs, 1H, NH). 13C-NMR (100.0 MHz, DMSO), δ, ppm: 27.5 (CH2), 35.2 (CH_2_CO), 35.3 (CH_2_CO), 47.4 (CH), 51.3 (NHCH), 116.5 (Cq), 116.6 (CHAr), 123.8 (Cq), 125.1 (CHAr), 125.5 (CHAr), 128.3 (CHAr), 128.7 (CHAr), 129.3 (CHAr), 152.1 (Cq), 169.9 (C=O), 170.7 (C=O), 171.9 (C=O). MS (MALDI, positive mode, matrix DHB) *m*/*z*: 420.4 (M + Na)+. Anal. Calcd. For C_20_H_23_N_5_O_4_ (397.44) C, 60.44; H, 5.83; N, 17.62. Found: C, 60.78; H, 6.07; N, 17.86.

4-Methylmercapto -2-(2-(9H-xanthen-9-yl)acetamido) butanhydrazide (**7h**).

White crystals, yield 61%. mp 124–125 °C. 1H NMR spectrum, (400 MHz, DMSO d6), δ, ppm (J, Hz): 2.05 (s, 3 H, SCH_3_), 2.42–2.63 (m, 4 H, CH_2_CO, CH_2_), 3.06–3.16 (m, 2 H, CH_2_), 3.73–3.75 (m, 1 H, NCH), 4.22 (bs, 2H, NH_2_), 4.50–4.60 (m, 1 H, CH), 5.90 (bs, 1 H, NH), 7.08–7.13 (m, 5 H, Ar-H), 7.19–7.36 (m, 3 H, Ar-H), 9.23 (bs, 1 H, NH). 13C-NMR (100.0 MHz, DMSO d6), δ, ppm: 15.3 (SCH_3_), 29.6 (CH_2_), 31.4 (CH_2_), 35.9 (CH_2_CO), 47.4 (CH), 51.4 (CH), 116.5 (Cq), 116.8 (CHAr), 123.4 (Cq), 123.6 (CHAr), 124.5 (Cq), 128.0 (CHAr), 128.7 (CHAr), 152.1 (Cq), 170.0 (C=O), 172.9 (C=O). MS (MALDI, positive mode, matrix DHB) m/z: 408.4 (M + Na)+. Anal. Calcd. For C_20_H_23_N_3_O_3_S (385.48) C, 62.32; H, 6.01; N, 10.90. Found: C, 62.56; H, 5.88; N, 11.04.

### 4.2. Biological Studies

#### 4.2.1. MTT Assay

Human colorectal carcinoma (HCT-116) cells were purchased from the American Type Culture Collection (ATCC^®^, Manassas, VA, USA; ATCC^®^ CCL-247™) and used to examine the impact of compounds (**5a**, **5b**, **5c**, **5d**, **5e**, **5f**, **5g**, **6a**, **6b**, **6c**, **6d**, **6e**, **6f**, **6g**, **6h**, **7b**, **7f**, **7g**, **7h**). The MTT assay was followed as per previously published studies [35,36]. Cells were seeded in the 96-well plates containing special media of DMEM and kept in CO2 incubator for 24 h. Once reaching 70–80% confluence, the cells were treated with compounds (**5a**, **5b**, **5c**, **5d**, **5e**, **5f**, **5g**, **6a**, **6b**, **6c**, **6d**, **6e**, **6f**, **6g**, **6h**, **7b**, **7f**, **7g**, **7h**) with dosages (50–400 µM) for 48 h and processed for MTT assay. Cisplatin (5–50 µM) served as positive control. The plates were examined under a plate reader supplied by Bio-Tek Instruments, Winooski, VT, USA at 570 nm wavelength and optical density (OD) was obtained to calculate the percentage of cell viability. The data are presented in the graph as mean ± standard deviation obtained from triplicates and statistically evaluated by GraphPad Version 6.0 Prism Software USA.

#### 4.2.2. Apoptotic DAPI Nuclear Staining

To examine the impact of the treatment of the compounds on the nucleus of HCT-116 cancer cells, we have selected the compounds that have shown the highest cancer cell death due to treatment. We treated the cells with compounds (**7b**) (66.96 µM) and (**7h**) (72.48 µM) for 48 h. After that, cells were treated with paraformaldehyde and then labeled with DAPI dye [37]. The blue fluorescent staining was examined under a confocal scanning microscope (Zeiss, Oberkochen, Germany).

#### 4.2.3. Image Acquisition

Fluorescence microscopy was used to acquire images of HCT-116 cells under two conditions: untreated (control) and treated with compound **7h**. All images were captured at the same magnification and exposure settings to ensure consistency and comparability across the datasets.

- Image processing

The acquired images were imported into CellProfiler 4.2.8 for processing. This step involved adjusting the image contrast, reducing background noise, and enhancing the signal-to-noise ratio to ensure clear differentiation between cellular objects and background. Preprocessing steps such as grayscale conversion and intensity normalization were applied uniformly across all samples.

- Identification of Primary and Secondary Objects

Primary Objects (Nuclei): CellProfiler was used to identify primary objects, such as nuclei, within the images. Automated thresholding algorithms were employed to distinguish nuclei from the background based on intensity differences.

Secondary Objects (Cells): After identifying the nuclei, CellProfiler expanded the segmentation to identify whole cells (secondary objects) by defining cell boundaries around each nucleus. This step allowed for the accurate detection and delineation of individual cells, even in densely populated regions.

- Quantitative Measurements

CellProfiler provided quantitative data for each identified object, including the number of nuclei and cells, their size (diameter), and the area covered by these objects. For nuclei, metrics such as the 10th, median, and 90th percentile diameters were calculated to evaluate changes in nuclear size and distribution due to treatment. For whole cells, the software calculated the total area covered by cells as a percentage of the image area, reflecting the changes in cell density and proliferation status between treated and untreated samples.

- Thresholding and Filtering.

Consistent thresholding parameters were set within CellProfiler to ensure uniform object identification criteria across all images. The threshold values were optimized to effectively separate objects of interest (nuclei and cells) from the background. Declumping algorithms and smoothing filters were applied to separate closely packed cells, ensuring accurate counting and measurement, particularly in control images where cells were densely clustered.

- Data Analysis and Interpretation.

The quantitative data obtained from CellProfiler were analyzed to compare the effects of compound **7h** on HCT-116 cells against untreated control cells. Key parameters such as the reduction in cell number, changes in object size, and decreases in area covered by cells were assessed to determine the compound’s antiproliferative and cytotoxic effects. By utilizing CellProfiler, an advanced and automated image analysis tool, we obtained precise and reproducible data on cell proliferation and morphological changes. This enabled a detailed evaluation of the potential anticancer effects of compound **7h** on HCT-116 cells.

### 4.3. Molecular Docking Studies

#### 4.3.1. Software

The molecular modelling software Maestro by Schrödinger (Version 2024-4 software release) was used for computational studies.

#### 4.3.2. Crystal Structures

The X-ray coordinates of potassium ion channel TASK-1 with a bound inhibitor (PDB IDs: 6RV4) were obtained from the Research Collaboratory for Structural Bioinformatics (RCSB) Protein Data Bank (PDB) [38].

#### 4.3.3. Protein Preparation

Protein preparation was performed using the Protein Preparation Workflow on Maestro (Version 2024-4 software release) at a pH of 7.4 with adjusting ionization states. The targets’ structures were then minimized using OPLS3 force field with a default value for rmsd of 0.30 Å for non-hydrogen atoms after excluding water molecules and adding polar hydrogens [39].

#### 4.3.4. Receptor Grid Generation

A grid box of 20 Å3 was generated for the binding site at the center of the bound ligand with a 1.00 van der Waals radius, along with a cutoff of 0.25 for partial charges without any constraints.

#### 4.3.5. Ligand Library Preparation

The ligands were prepared using LigPrep, by generating the most likely ionization states at a pH of 7 ± 2, followed by structure optimization with the OPLS3 force field [39].

#### 4.3.6. Validation of Molecular Docking

The crystal ligand was docked into the target protein using the same docking criteria used for the prepared ligands, and then the root mean square deviation (RMSD) of the alignment of the lowest binding energy conformation with the crystal conformation was calculated using Maestro’s structure superimposition tool [39,40].

#### 4.3.7. Molecular Docking

The extra precision mode (XP) on glide was used for all docking procedures with a van der Waals (vdw) radius scaling factor of 0.80 and a partial charge cut-off of 0.15 and with no constraints, and the XP scores were calculated. Molecular mechanics–generalized Born surface area (MM-GBSA) binding free energy was computed using the Prime MM-GBSA module [40,41].

#### 4.3.8. Molecular Dynamics (MD) Simulations

Molecular dynamics (MD) simulations were performed to evaluate the stability and dynamic behavior of the protein–ligand complexes using the Desmond module of the Schrödinger suite [42,43]. The X-ray crystal structure of the potassium ion channel TASK-1 (K2P3.1) (PDB ID: 6RV4) was used as the protein model. The system corresponds to a dimeric construct comprising chains A and B, and the selected ligand-bound complex 7h was prepared using the System Builder tool. The simple point charge (SPC) water model was used to add water to each system in an orthorhombic simulation box with the right buffer distances. To make the system neutral, counterions were added, and sodium and chloride ions were added to reach a physiological salt concentration of 0.15 M. For all of the simulations, we used the OPLS5 force field [44].

The Desmond Simulation Interaction Diagram tool was used to carry out trajectory analyses. To check the stability and equilibration of the system, the root mean square deviation (RMSD) of the heavy atoms in the protein backbone and the ligand was calculated. The “Lig fit Prot” RMSD metric was applied to observe how stable the ligand was compared to the protein binding pocket [45]. The root mean square fluctuation (RMSF) of protein Cα atoms was utilized to measure the flexibility of the residues and to find areas of the protein structure that were changing. Furthermore, secondary structure elements were evaluated during the whole simulation to check the stability of the structure and the stability of the conformation [46]. Protein–ligand interactions were analyzed over the trajectory and categorized into hydrogen bonds, hydrophobic interactions, ionic contacts, water bridges, and halogen bonds. Interaction timelines and contact persistence were evaluated, and residues exhibiting significant interaction occupancy over the simulation period were identified as key contributors to ligand binding [46].

## 5. Conclusions

A series of 27 N-Alkyl-2-(9H-xanthen-9-yl)acetamides and methyl 2-(2-(9H-xanthen-9-yl)acetamido)alkanoates were synthesized via azide coupling, and corresponding hydrazides were obtained using hydrazine. Compounds **5f**, **5g**, **6c**, **6d**, **6f**, **6g**, **7b**, **7f**, and **7h** exhibited good cytotoxic activity (IC50 66.97–99.62 µM). DAPI staining and CellProfiler analysis for compound **7h** revealed reduced cell proliferation, nuclear size, and cellular density, likely through cell cycle arrest and apoptosis. Docking and molecular dynamics studies indicated that TASK-1 (K2P3.1) inhibition is a plausible mechanism, with stable binding interactions observed over 100 ns. These findings highlight compound **7h** as a promising lead for further molecular optimization and broader exploration as a TASK-1-targeted therapeutic in colorectal cancer.

## Figures and Tables

**Table 1:** List of compounds with IC50 values for HCT-116 cells.

Compound	IC_50_ µM
**5a**	103.28
**5b**	242.78
**5c**	109.41
**5d**	101.76
**5e**	135.19
**5f**	84.54
**5g**	77.56
**6a**	107.62
**6b**	105.13
**6c**	99.62
**6d**	79.38
**6e**	NI
**6f**	75.35
**6g**	76.2
**6h**	353.75
**7b**	66.97
**7f**	78.05
**7g**	210.77
**7h**	72.48
Cisplatin	18.25

NI: Non inhibitory.

**Table 2:** Calculated RMSD of the binding poses of the synthesized xanthene derivatives relative to 7h.

Compound	RMSD
**7g**	0.3886
**6f**	0.4164
**5f**	0.9607
**6d**	1.0362
**6g**	1.4008
**5e**	1.5547
**5g**	1.7697
**7f**	1.8554
**5c**	1.8771
**5d**	1.8987
**6a**	1.9657
**6C**	2.1039
**6b**	2.2059
**5a**	2.2136
**6h**	2.2272
**5b**	2.3024
**7b**	2.3479

**Table 3:** Calculated XP scores and binding affinities (MM-GBSA) for the synthesized xanthene derivatives and crystal ligand ranked according to their MM-GBSA score.

Compound	XP Score (kcal/mol)	MM-GBSA (kcal/mol)
**5g**	−11.114	−63.17
**7g**	−8.914	−46.48
**5b**	−8.082	−44.56
**6b**	−8.064	−41.14
**6g**	−8.951	−40.19
**6a**	−7.476	−39.36
**5a**	−7.26	−38.42
**6d**	−7.963	−37.28
**5d**	−7.144	−36.99
**7h**	−9.349	−35.75
**5e**	−9.57	−33.31
**6h**	−8.811	−32.45
**6f**	−8.928	−32.1
**7b**	−7.658	−31.52
**7f**	−8.961	−29.33
**6e**	−8.716	−29.28
**6c**	−6.932	−26.32
**5c**	−7.307	−25.59
**5f**	−7.6	−16.39
Crystal Ligand	−10.015	−49.46

## Data Availability

The original contributions presented in this study are included in the article/Supplementary Material. Further inquiries can be directed to the corresponding authors.

## Supplementary Materials

The following supporting information can be downloaded at: https://www.mdpi.com/article/10.3390/ijms27094069/s1.
