# Supplementary material for: N-Substituted-2-(9H-Xanthen-9-yl)acetamide Derivatives Induce In Vitro Colon Cancer Cell Death via TASK-1 Inhibition: Lead Compounds for Further Optimization as TASK-1-Targeted Therapeutics in Colorectal Cancer
Source: Int J Mol Sci. 2026 May 1;27(9):4069. doi: 10.3390/ijms27094069 (PMC13164145; doi:10.3390/ijms27094069)
Supplement: Supplementary file 1 [file ijms-27-04069-s001.zip › ijms-4192423-supplementary.pdf]

N-Substituted-2-(9H-xanthen-9-yl)acetamide Derivatives Induce In Vitro Colon Cancer Cell Death via TASK-1 Inhibition: Lead Compounds for Further Optimization as TASK-1-Targeted Therapeutics in Colorectal Cancer

Supplementary material table of content

Figures	Page
Figure S1 ^{1}H NMR of **5a**.	4
Figure S2 ^{13}C NMR of **5a**.	4
Figure S3 MALDI of compound **5a**.	5
Figure S4 ^{1}H NMR of **5b**.	5
Figure S5 ^{13}C NMR of **5b**.	6
Figure S6 MALDI of compound **5b**.	6
Figure S7 ^{1}H NMR of **5c**.	7
Figure S8 ^{13}C NMR of **5c**.	7
Figure S9 MALDI of compound **5c**.	8
Figure S10 ^{1}H NMR of **5d**.	8
Figure S11 ^{13}C NMR of **5d**.	9
Figure S12 MALDI of compound **5d**.	9
Figure S13 ^{1}H NMR of **5e**.	10
Figure S14 ^{13}C NMR of **5e**.	10
Figure S15 MALDI of compound **5e**.	11
Figure S16 ^{1}H NMR of **5f**.	11
Figure S17 ^{13}C NMR of **5f**.	12
Figure S18 MALDI of compound **5f**.	12
Figure S19 ^{1}H NMR of **5g**.	13
Figure S20 ^{13}C NMR of **5g**.	13
Figure S21 MALDI of compound **5g**.	14
Figure S22 ^{1}H NMR of **5h**.	14
Figure S23 ^{13}C NMR of **5h**.	15
Figure S24 MALDI of compound **5h**.	15
Figure S25 ^{1}H NMR of **5i**.	16
Figure S26 ^{13}C NMR of **5i**.	16
Figure S27 MALDI of compound **5i**.	17
Figure S28 ^{1}H NMR of **6a**.	17
Figure S29 ^{13}C NMR of **6a**.	18
Figure S30 MALDI of compound **6a**.	18
Figure S31 ^{1}H NMR of **6b**.	19
Figure S32 ^{13}C NMR of **6b**.	19
Figure S33 MALDI of compound **6b**.	20
Figure S34 ^{1}H NMR of **6c**.	20
Figure S35 ^{13}C NMR of **6c**.	21

Figures	Page
Figure S36 MALDI of compound **6c**.	21
Figure S37 ^{1}H NMR of **6d**.	22
Figure S38 ^{13}C NMR of **6d**.	22
Figure S39 MALDI of compound **6d**.	23
Figure S40 ^{1}H NMR of **6e**.	23
Figure S41 ^{13}C NMR of **6e**.	24
Figure S42 MALDI of compound **6e**.	24
Figure S43 ^{1}H NMR of **6f**.	25
Figure S44 ^{13}C NMR of **6f**.	25
Figure S45 MALDI of compound **6f**.	26
Figure S46 ^{1}H NMR of **6g**.	26
Figure S47 ^{13}C NMR of **6g**.	27
Figure S48 MALDI of compound **6g**.	27
Figure S49 ^{1}H NMR of **6h**.	28
Figure S50 ^{13}C NMR of **6h**.	28
Figure S51 MALDI of compound **6h**.	29
Figure S52 ^{1}H NMR of **7a**.	29
Figure S53 ^{13}C NMR of **7a**.	30
Figure S54 MALDI of compound **7a**.	30
Figure S55 ^{1}H NMR of **7b**.	31
Figure S56 ^{13}C NMR of **7b**.	31
Figure S57 MALDI of compound **7b**.	32
Figure S58 ^{1}H NMR of **7c**.	32
Figure S59 ^{13}C NMR of **7c**.	33
Figure S60 MALDI of compound **7c**.	33
Figure S61 ^{1}H NMR of **7d**.	34
Figure S62 ^{13}C NMR of **7d**.	34
Figure S63 MALDI of compound **7c**.	35
Figure S64 ^{1}H NMR of **7e**.	35
Figure S65 ^{13}C NMR of **7e**.	36
Figure S66 MALDI of compound **7e**.	36
Figure S67 ^{1}H NMR of **7f**.	37
Figure S68 ^{13}C NMR of **7f**.	37
Figure S69 MALDI of compound **7f**.	38
Figure S70 ^{1}H NMR of **7g**.	38
Figure S71 ^{13}C NMR of **7g**.	39
Figure S72 MALDI of compound **7g**.	39
Figure S73 ^{1}H NMR of **7h**.	40
Figure S74 ^{13}C NMR of **7h**.	40
Figure S75 MALDI of compound **7h**.	41

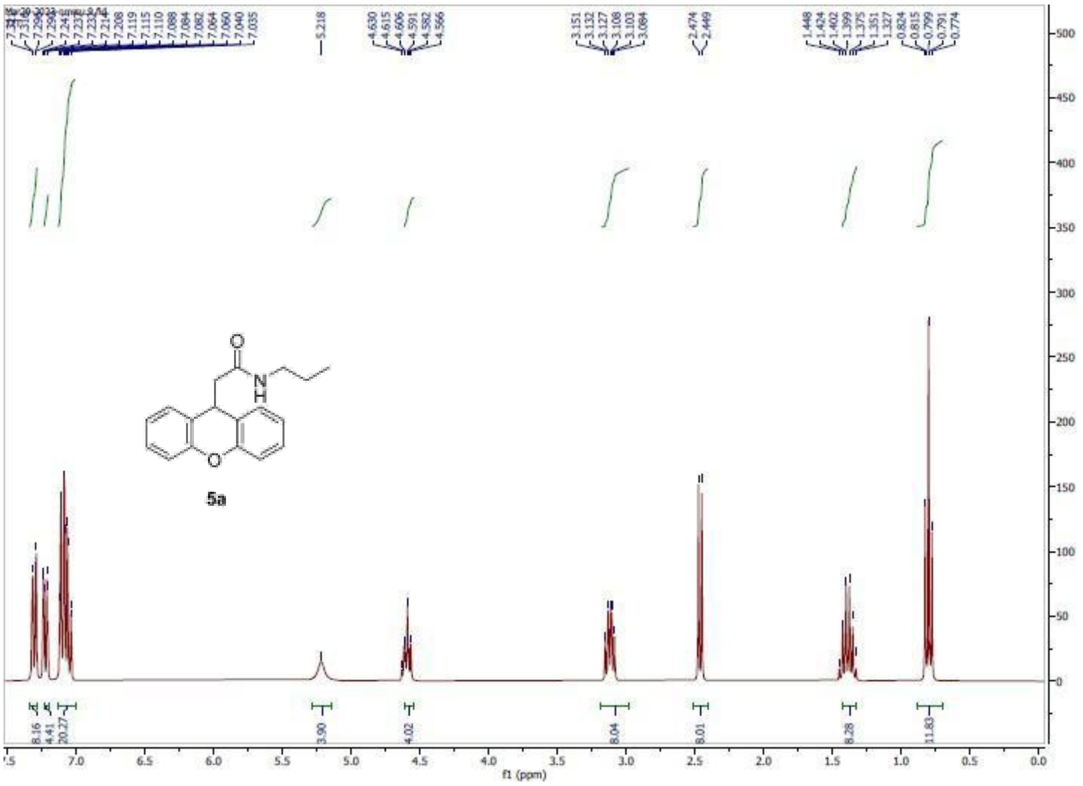

Figure S1. ^{1}H NMR of **5a**.

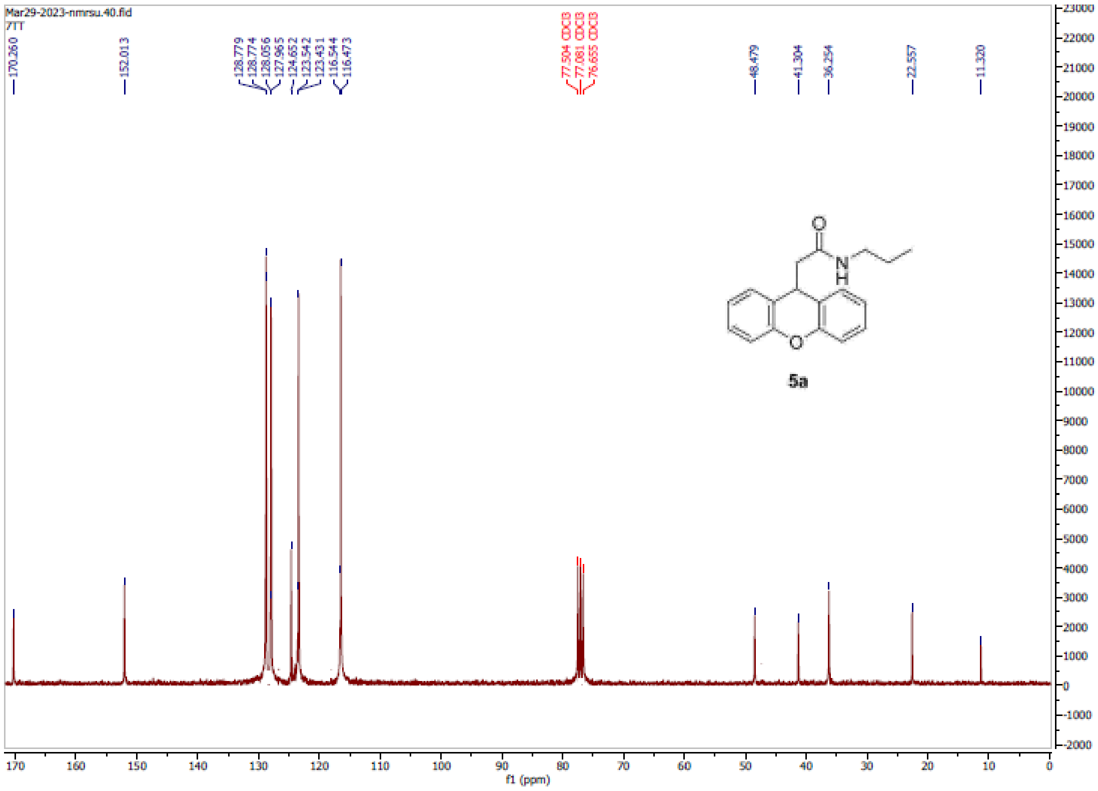

Figure S2. ^{13}C NMR of **5a**.

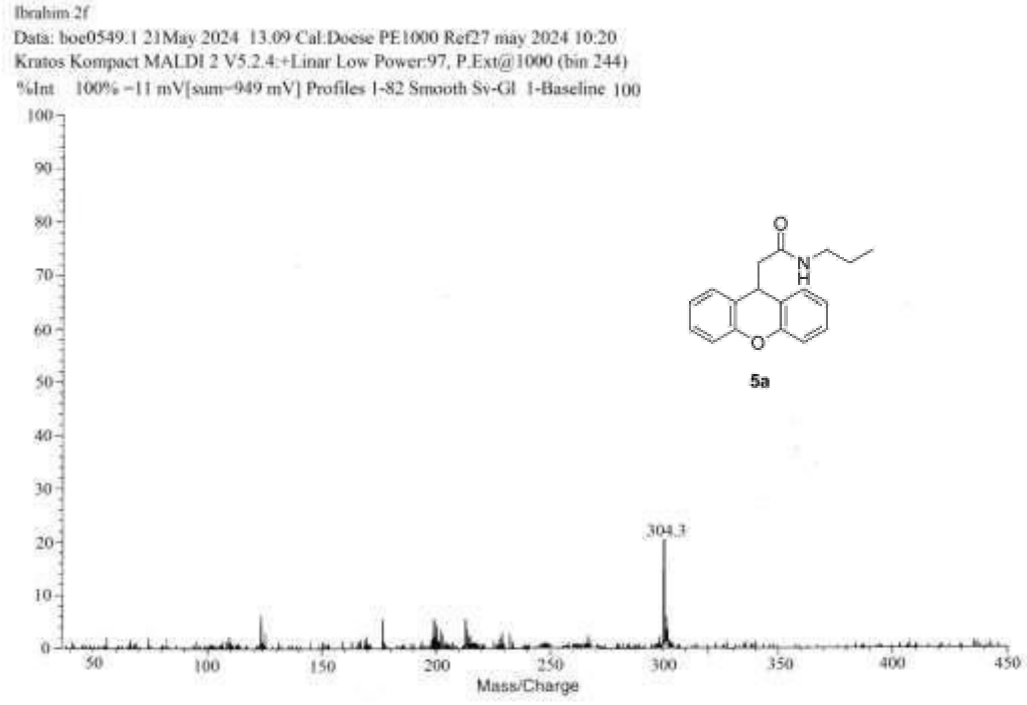

Figure S3. MALDI of compound **5a**.

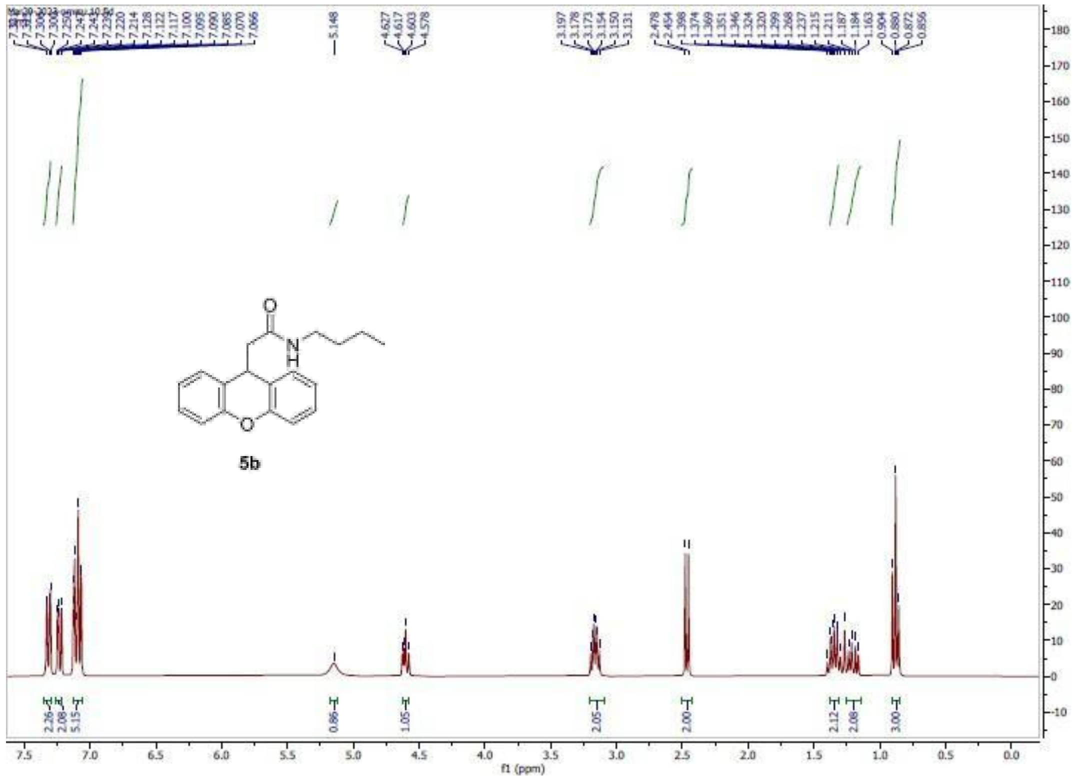

Figure S4. ^{1}H NMR of **5b**.

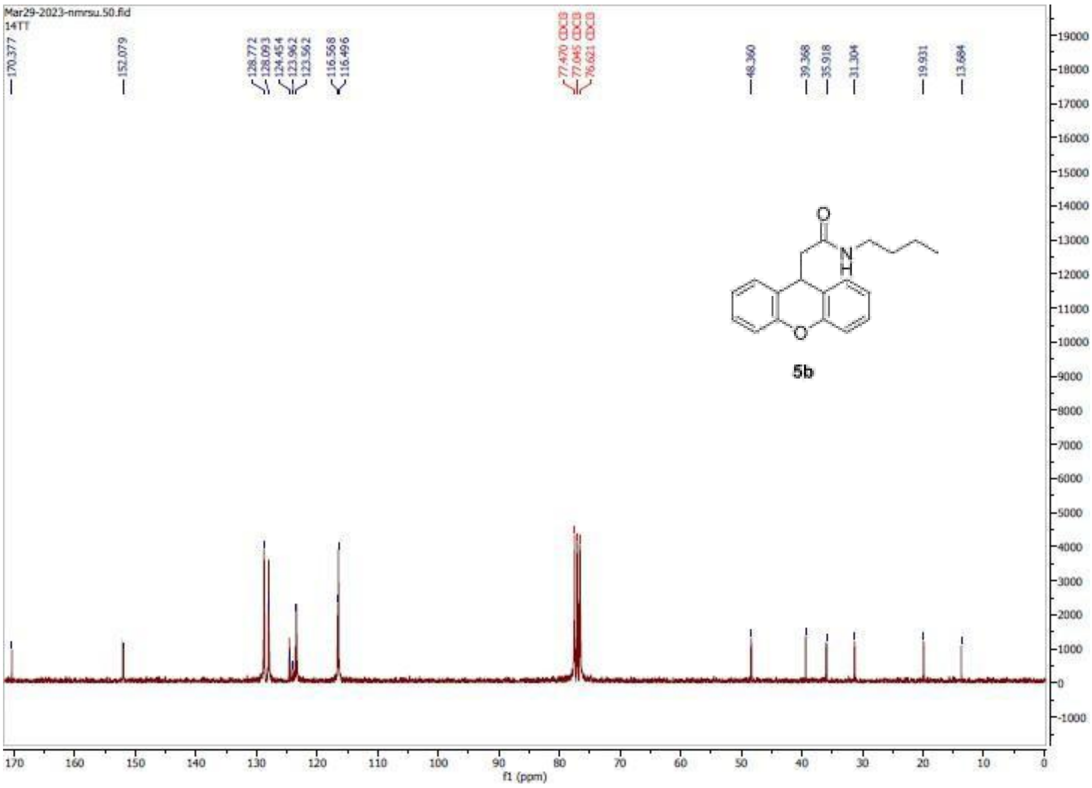

Figure S5. ^{13}C NMR of **5b**.

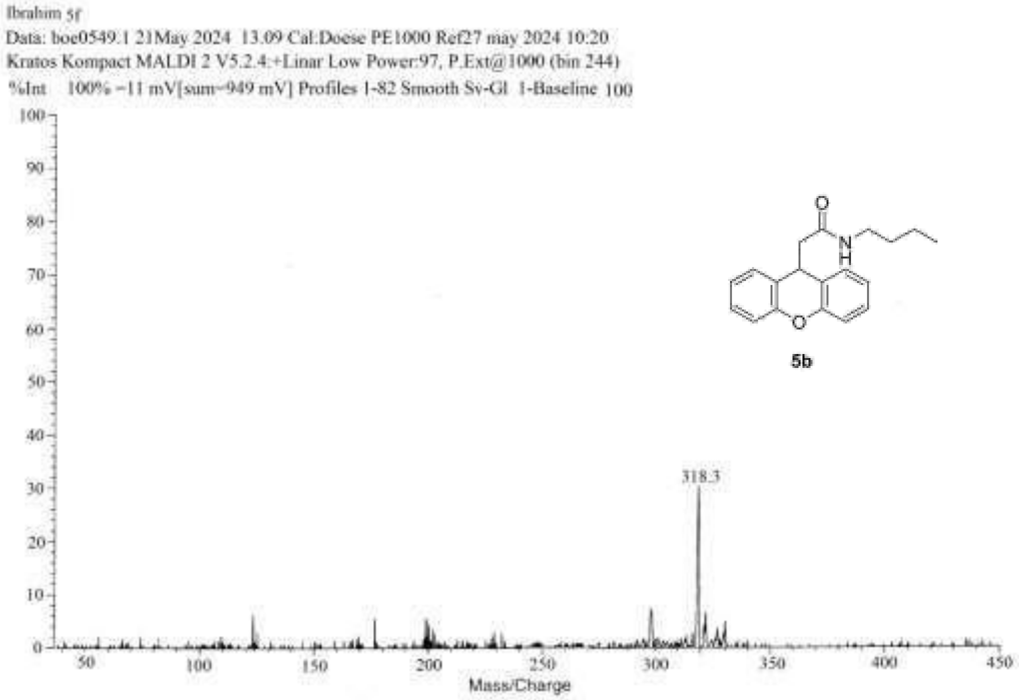

Figure S6. MALDI of compound **5b**.

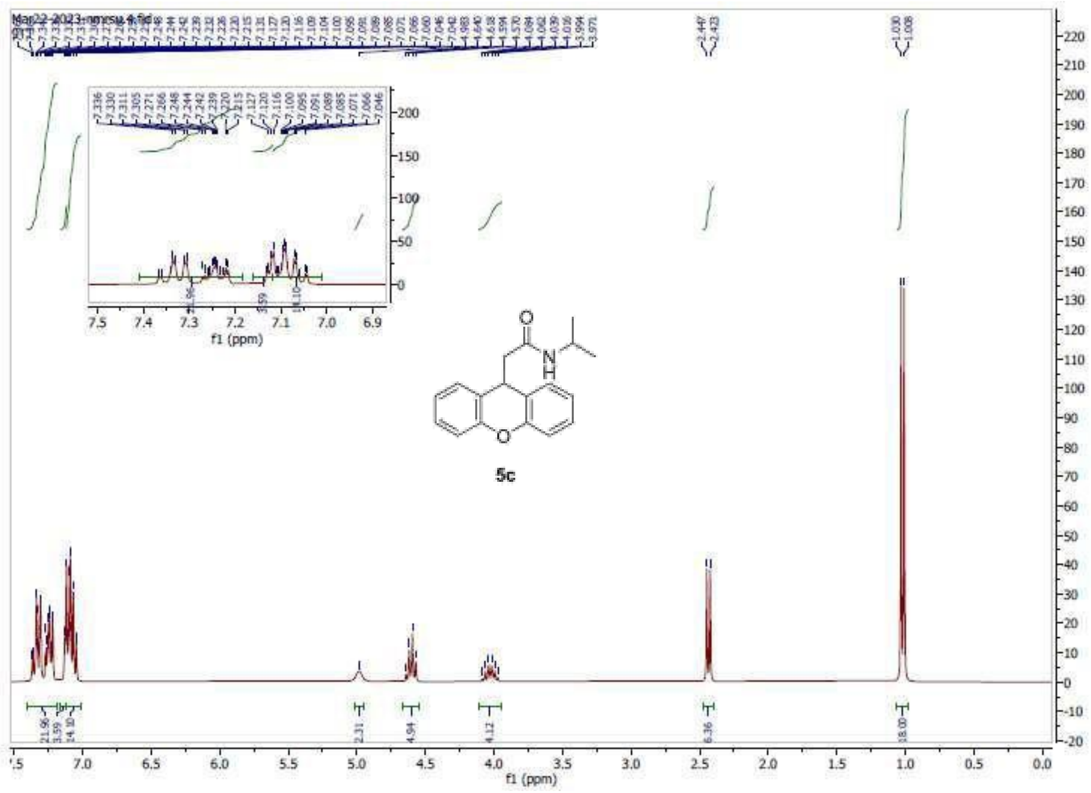

Figure S7. ^{1}H NMR of **5c**.

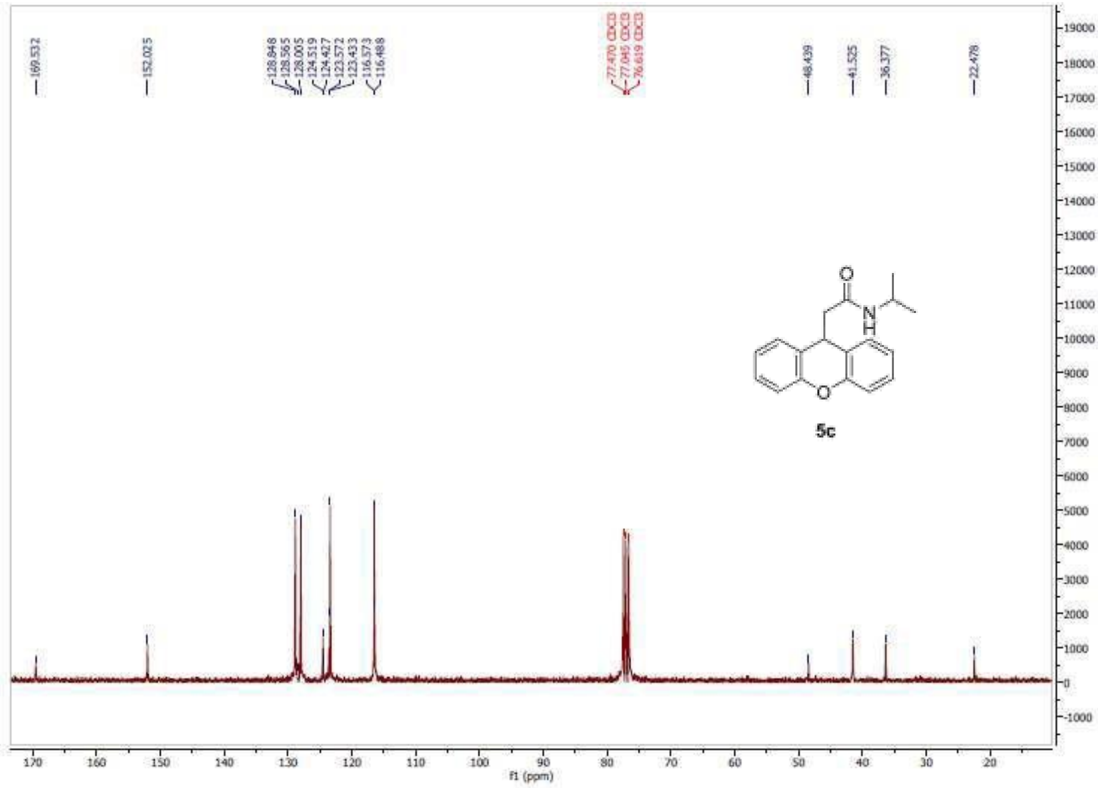

Figure S8. ^{13}C NMR of **5c**.

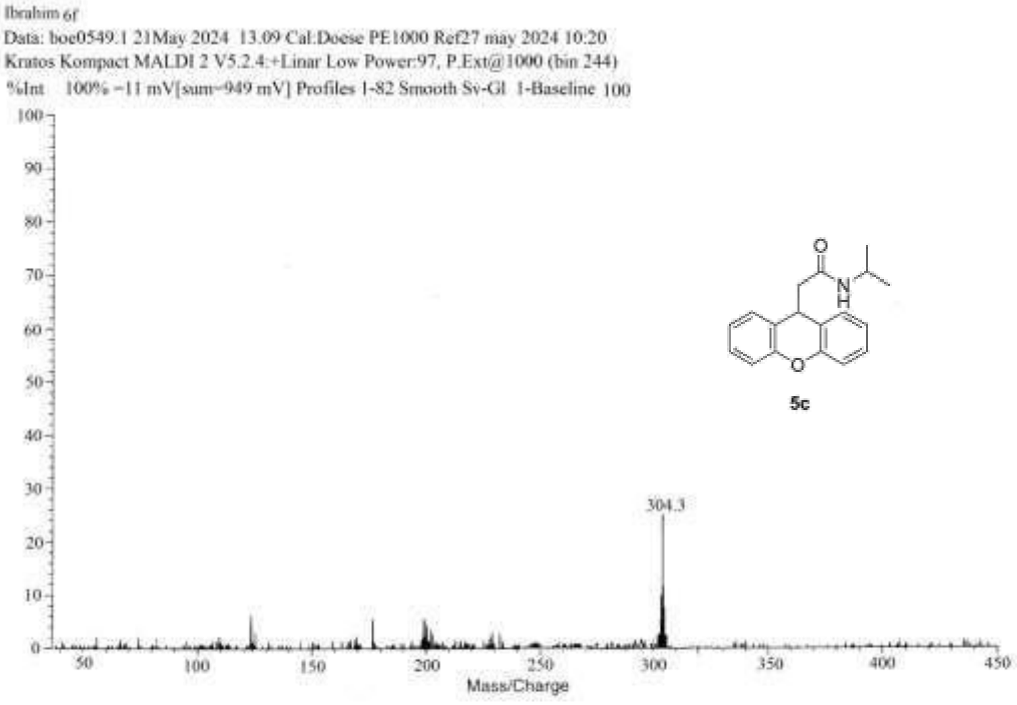

Figure S9. MALDI of compound **5c**.

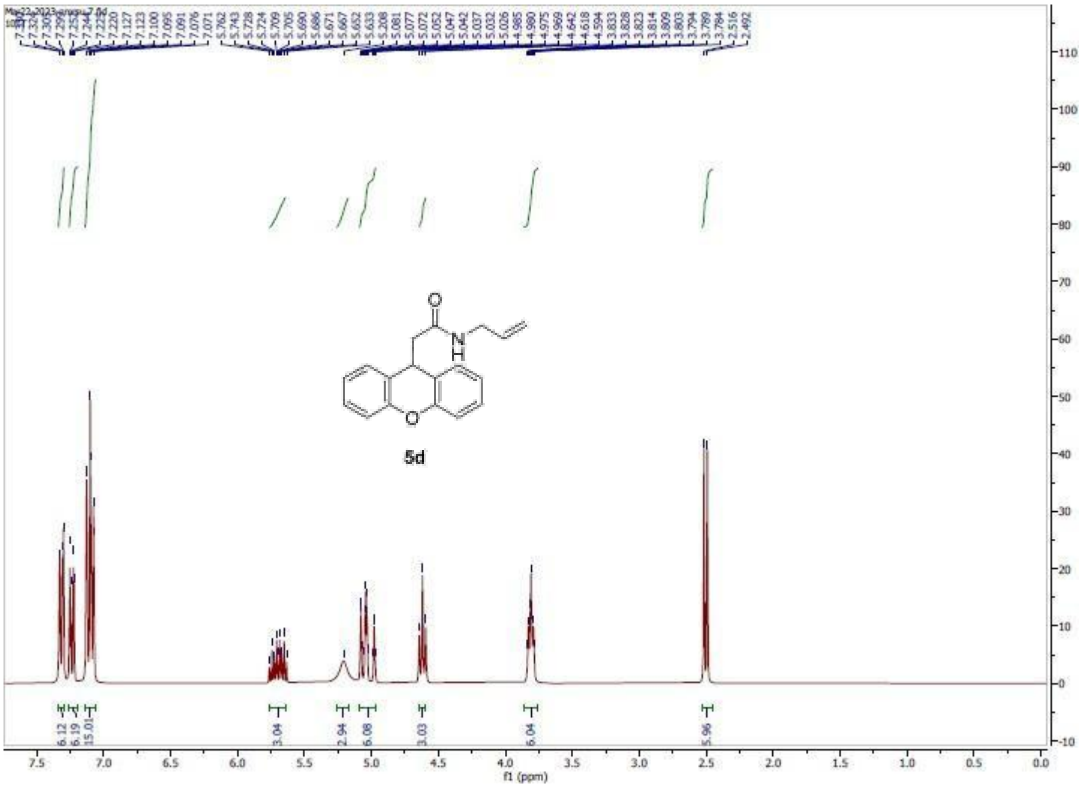

Figure S10. ^{1}H NMR of **5d**.

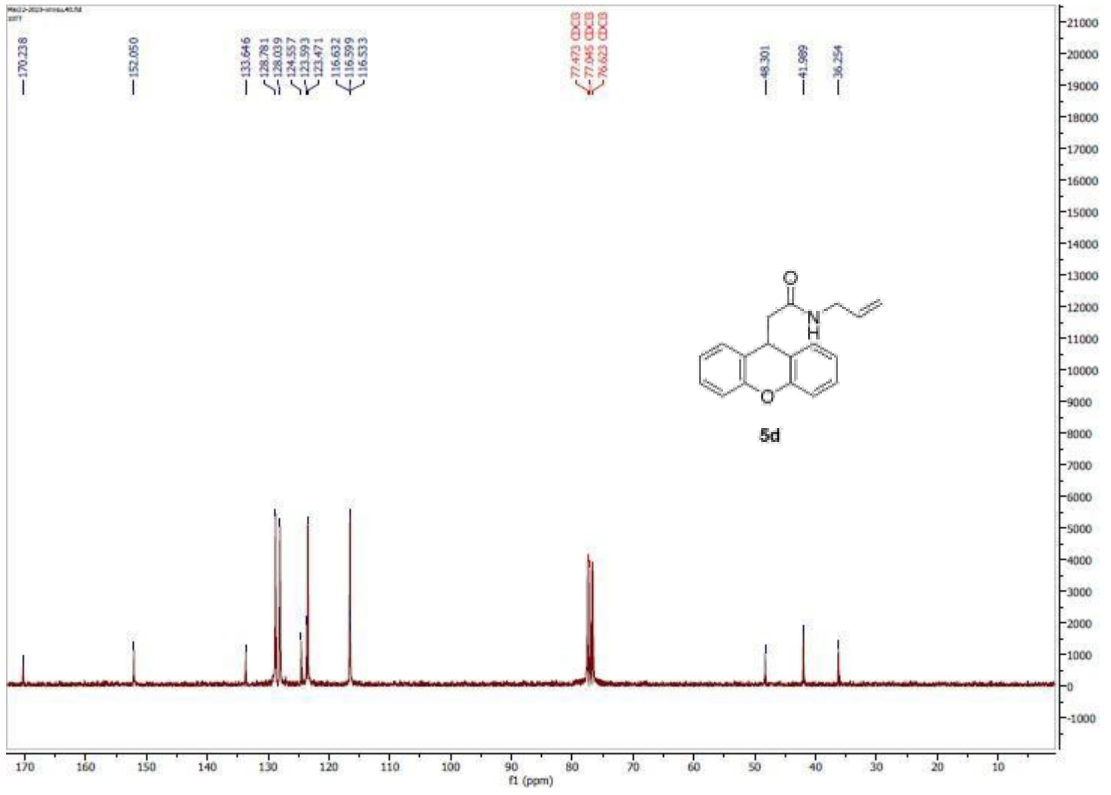

Figure S11. ^{13}C NMR of **5d**.

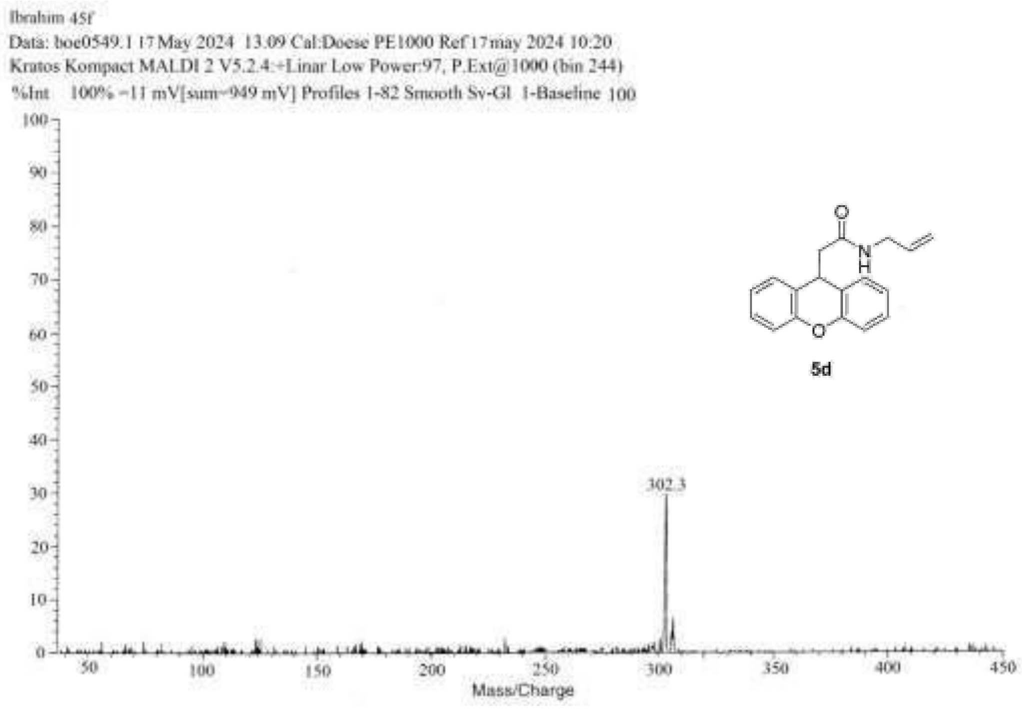

Figure S12. MALDI of compound **5d**.

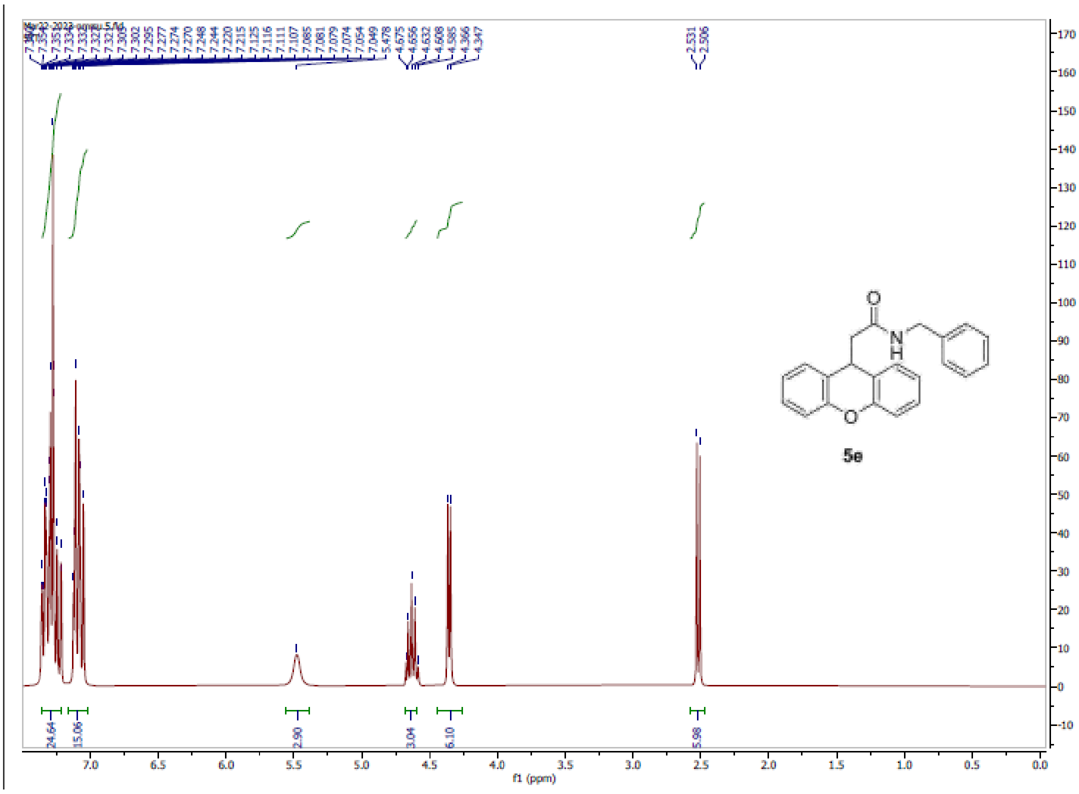

Figure S13. ^{1}H NMR of **5e**.

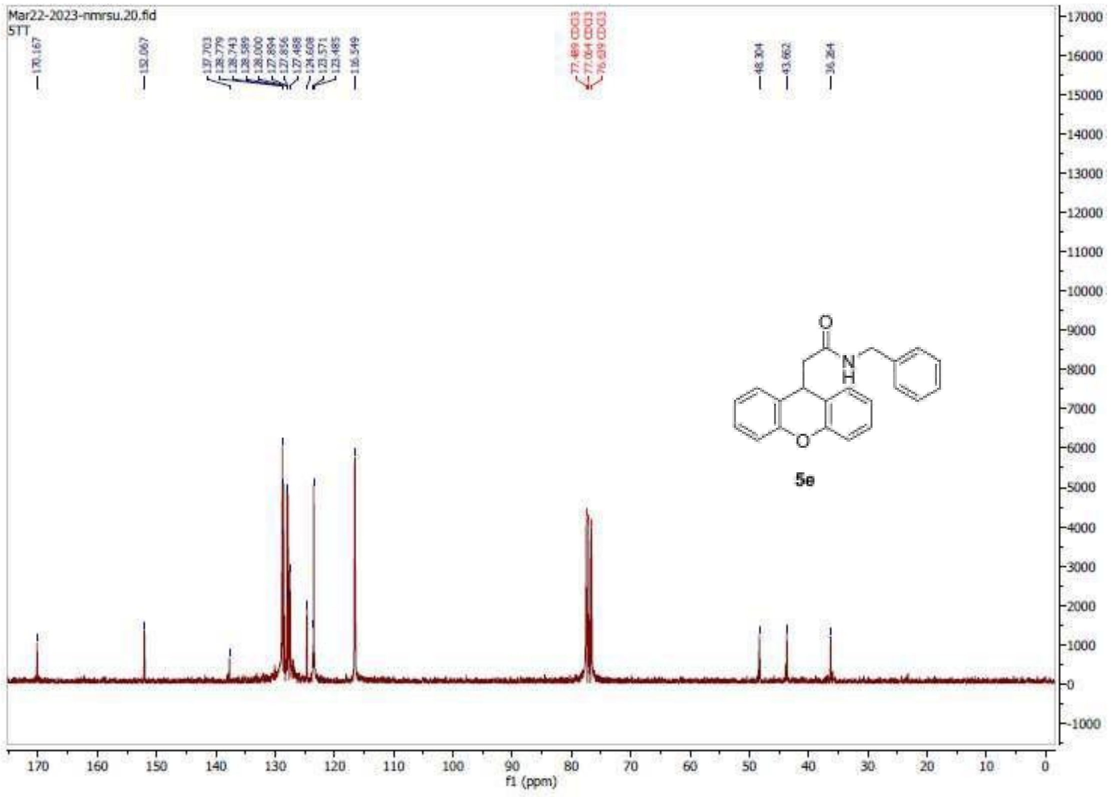

Figure S14. ^{13}C NMR of **5e**.

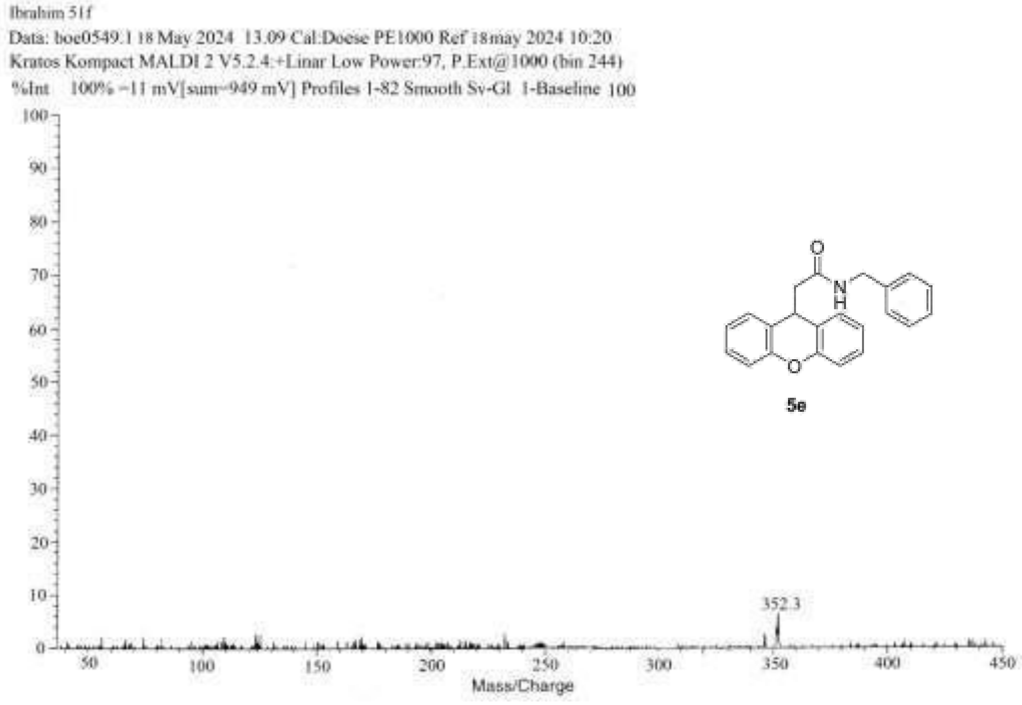

Figure S15. MALDI of compound **5e**.

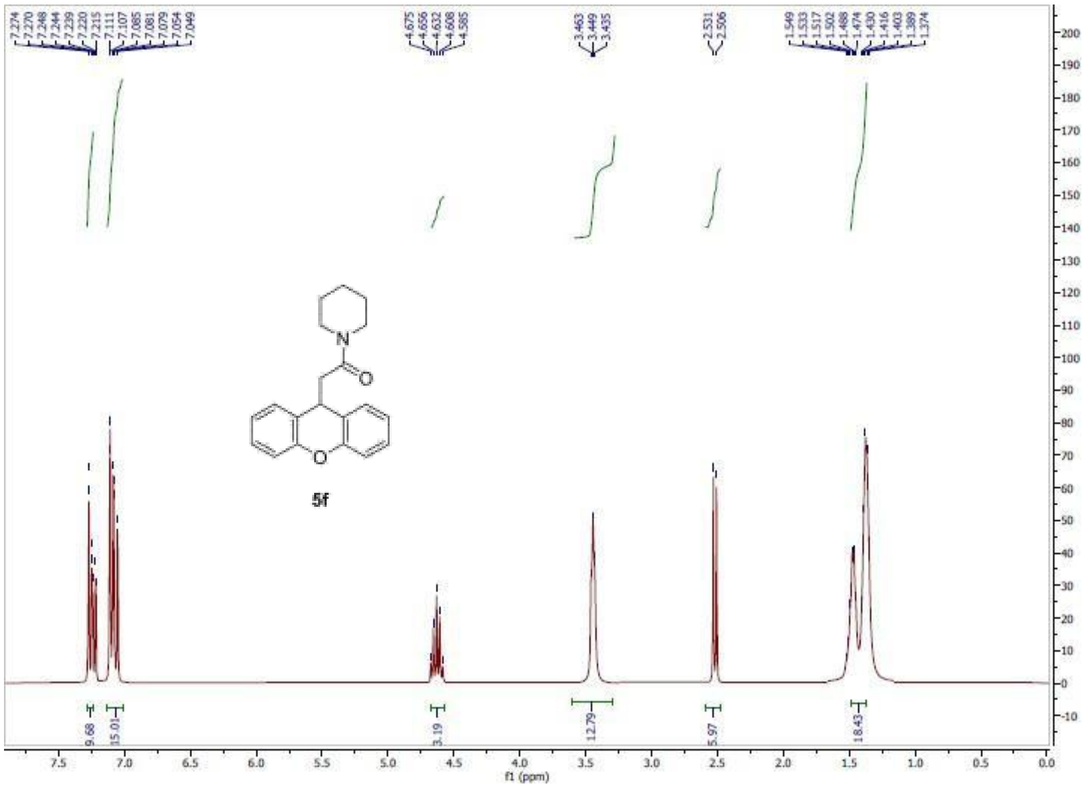

Figure S16. ^{1}H NMR of **5f**.

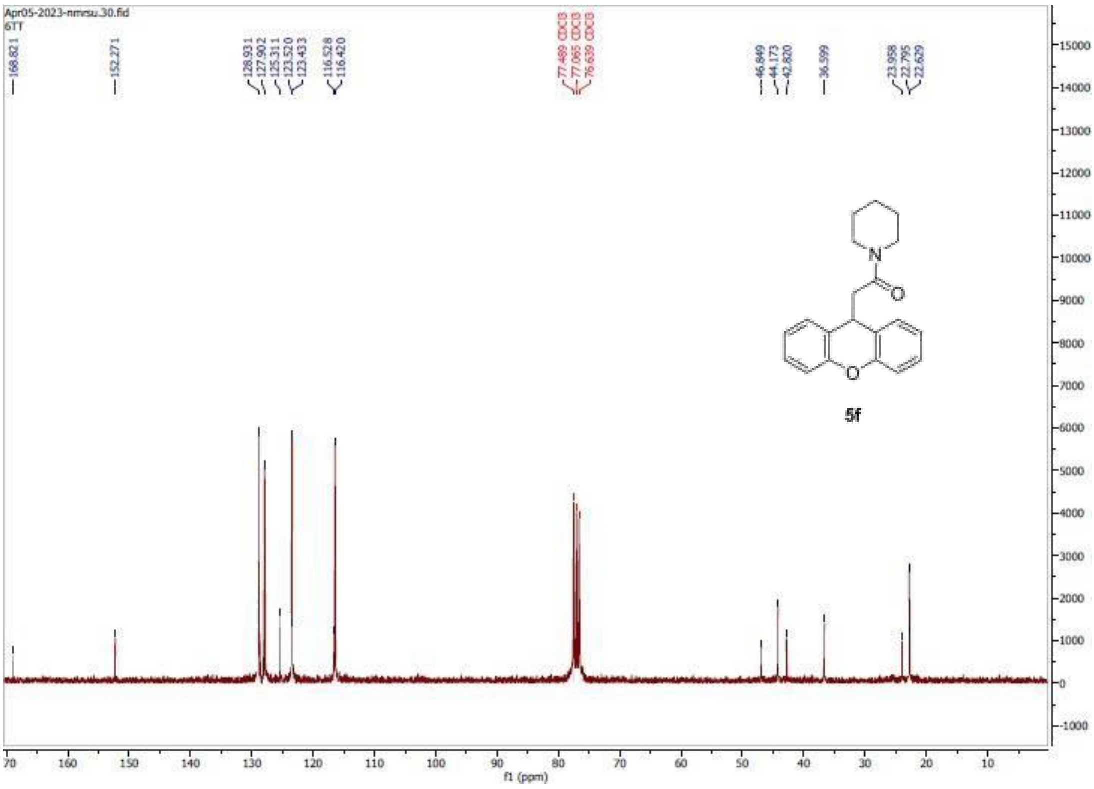

Figure S17. ^{13}C NMR of **5f**.

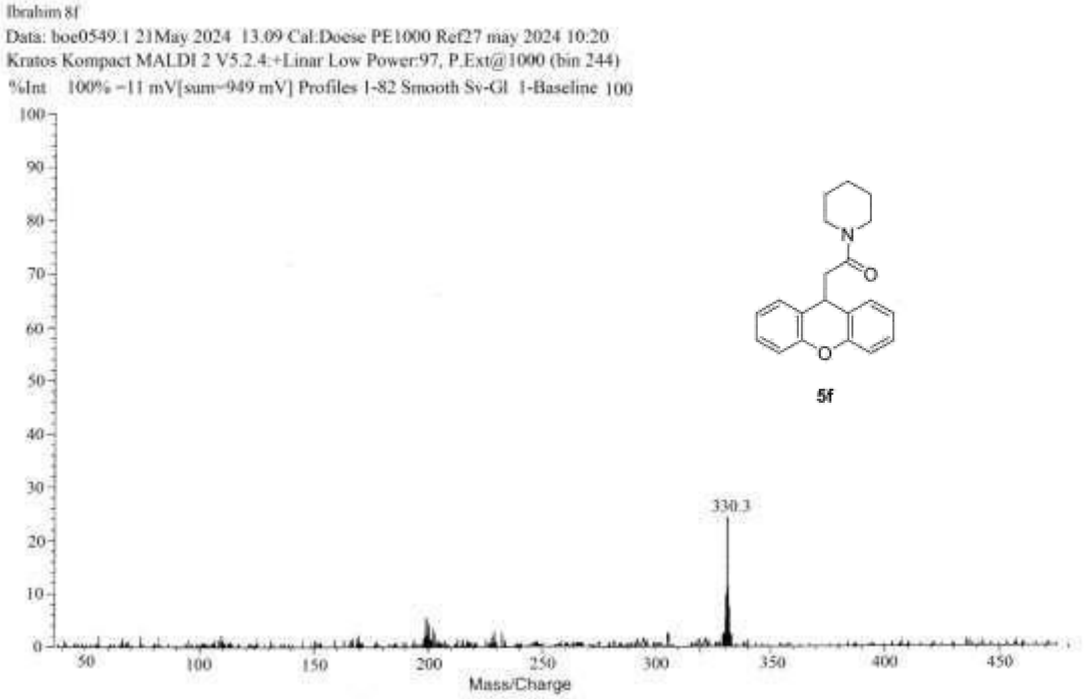

Figure S18. MALDI of compound **5f**.

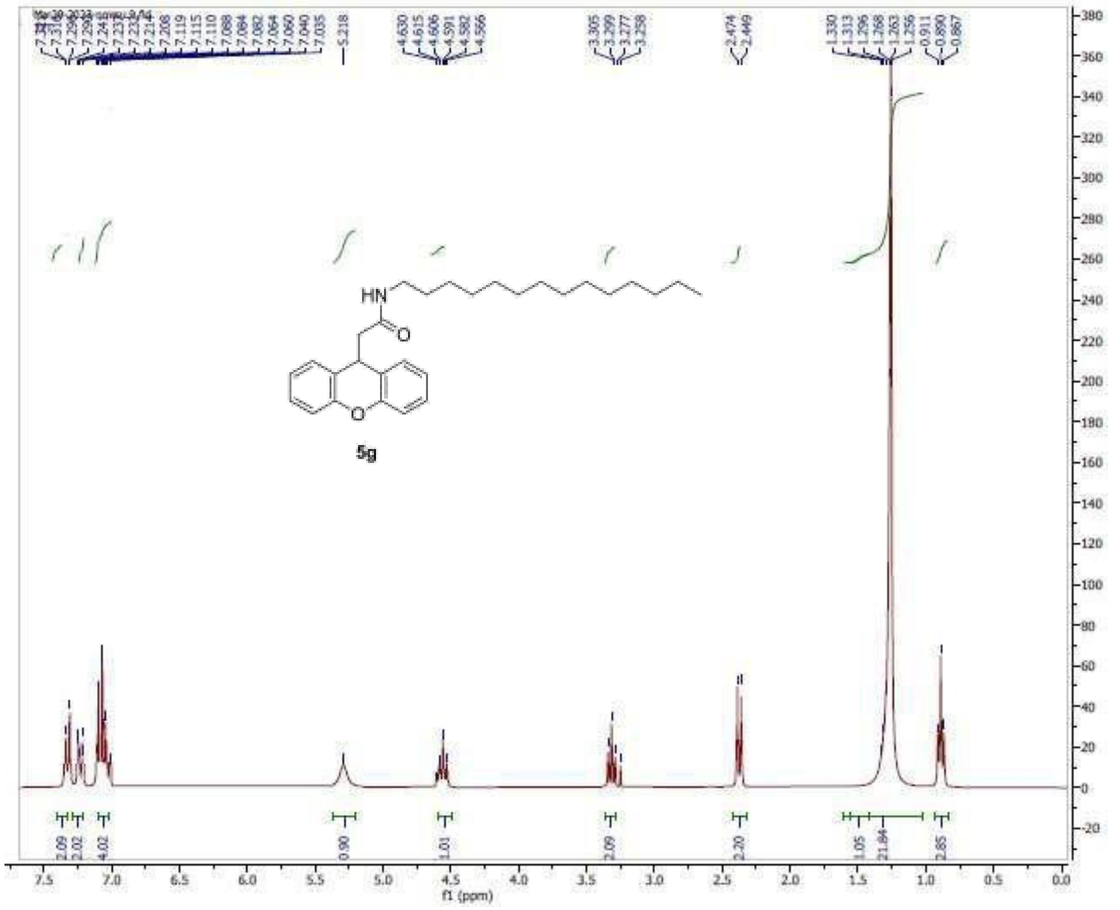

Figure S19. ^{1}H NMR of **5g**.

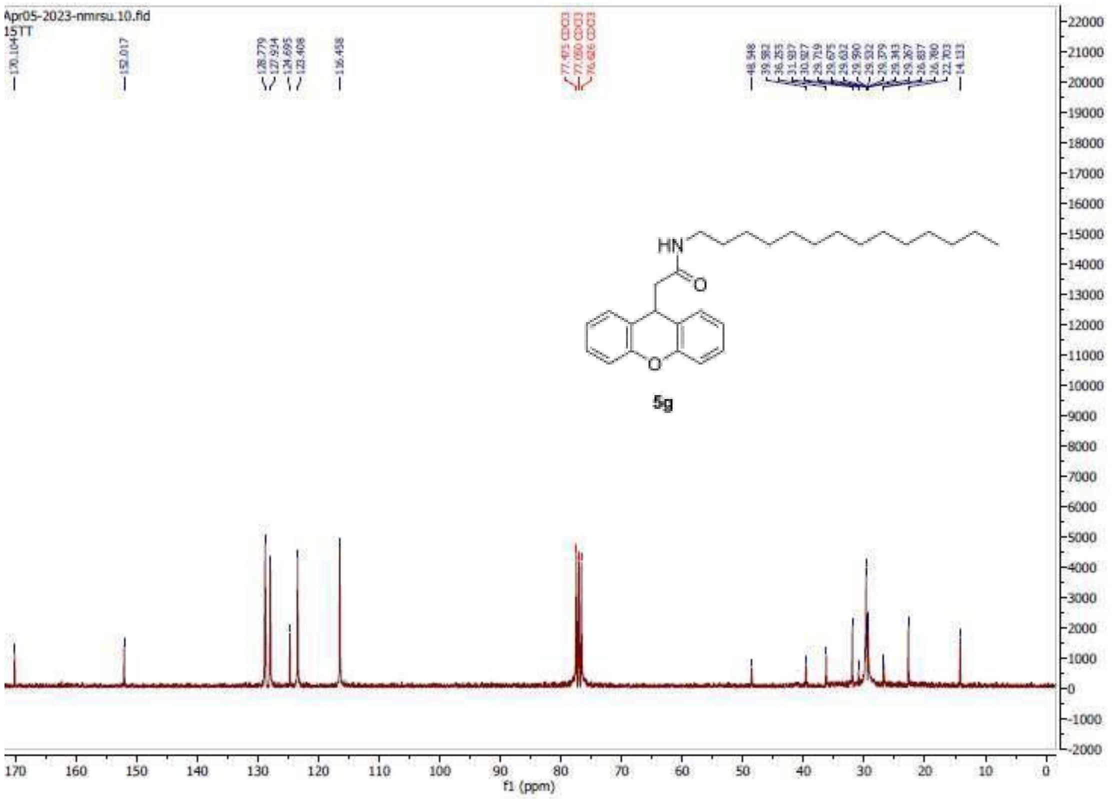

Figure S20. ^{13}C NMR of **5g**.

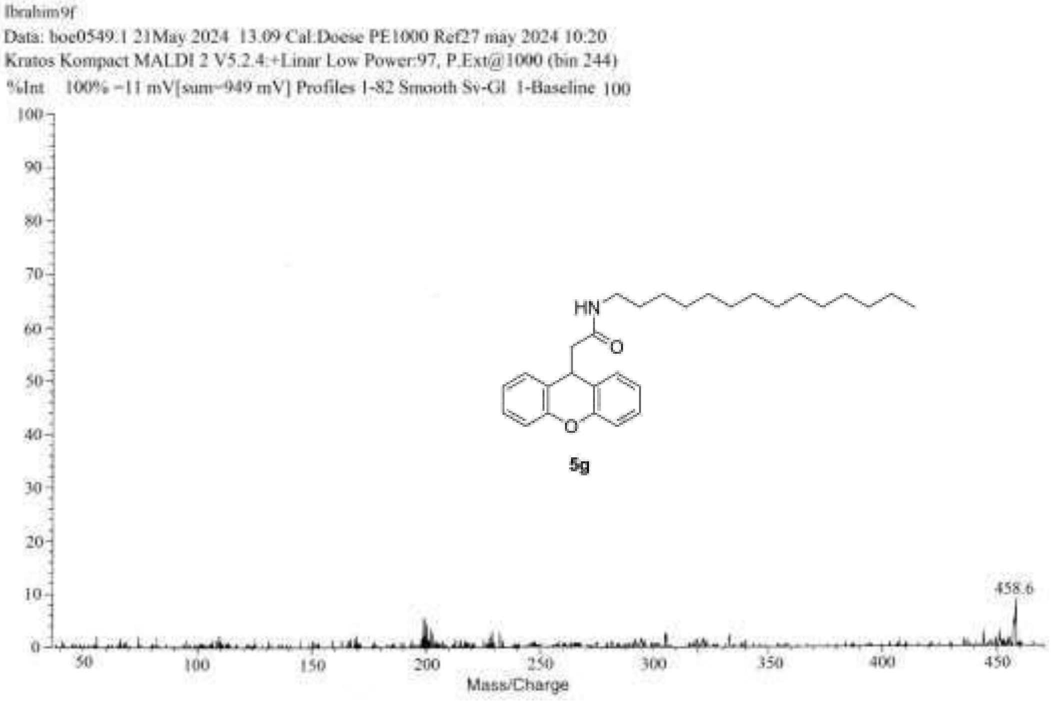

Figure S21. MALDI of compound **5g**.

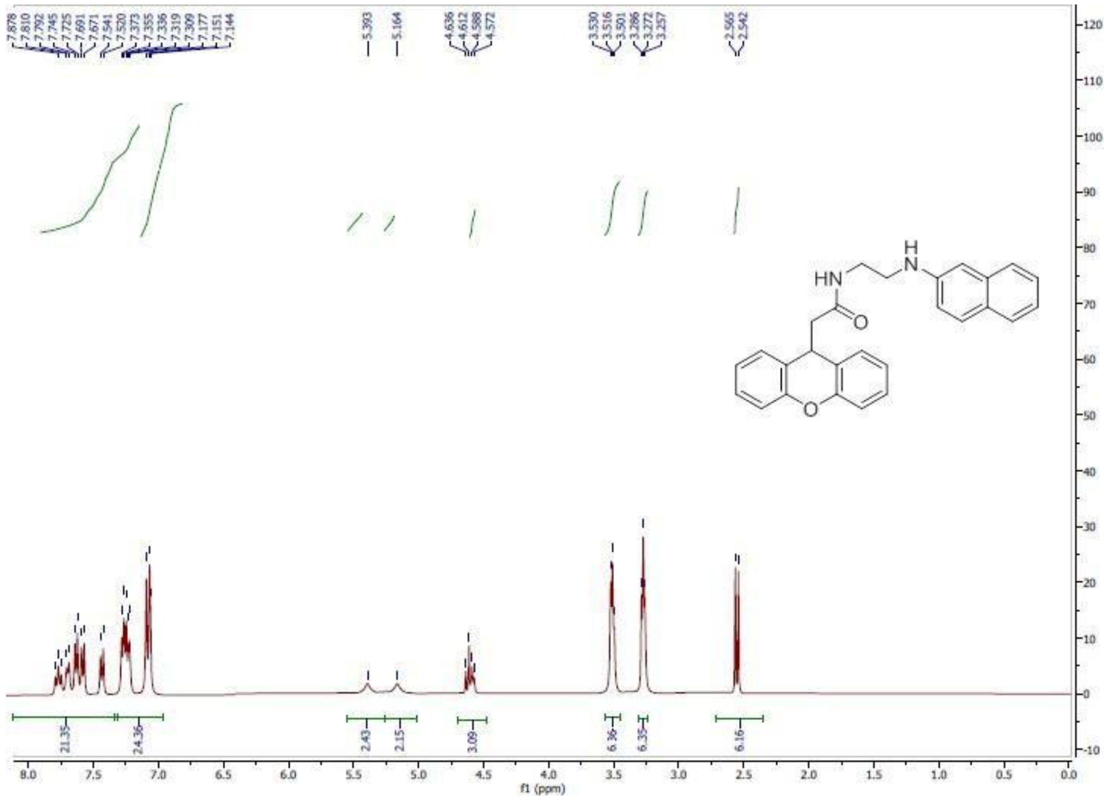

Figure S22. ^{1}H NMR of **5h**.

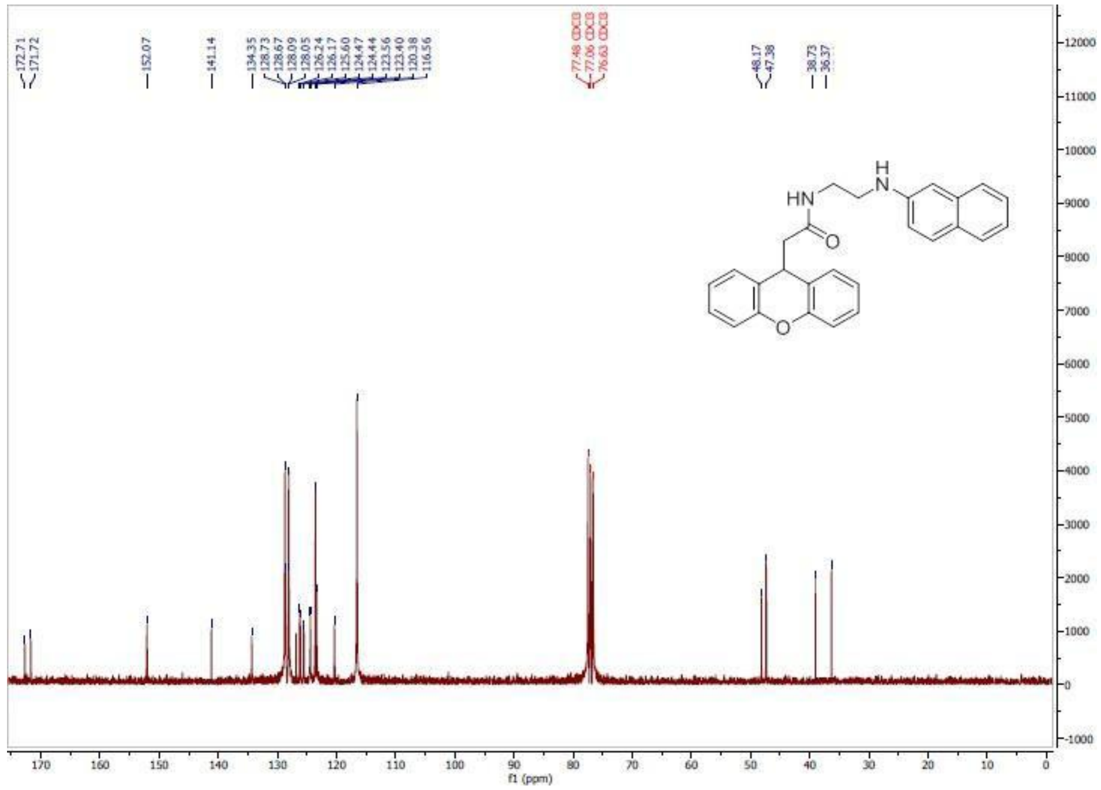

Figure S23. ^{13}C NMR of **5h**.

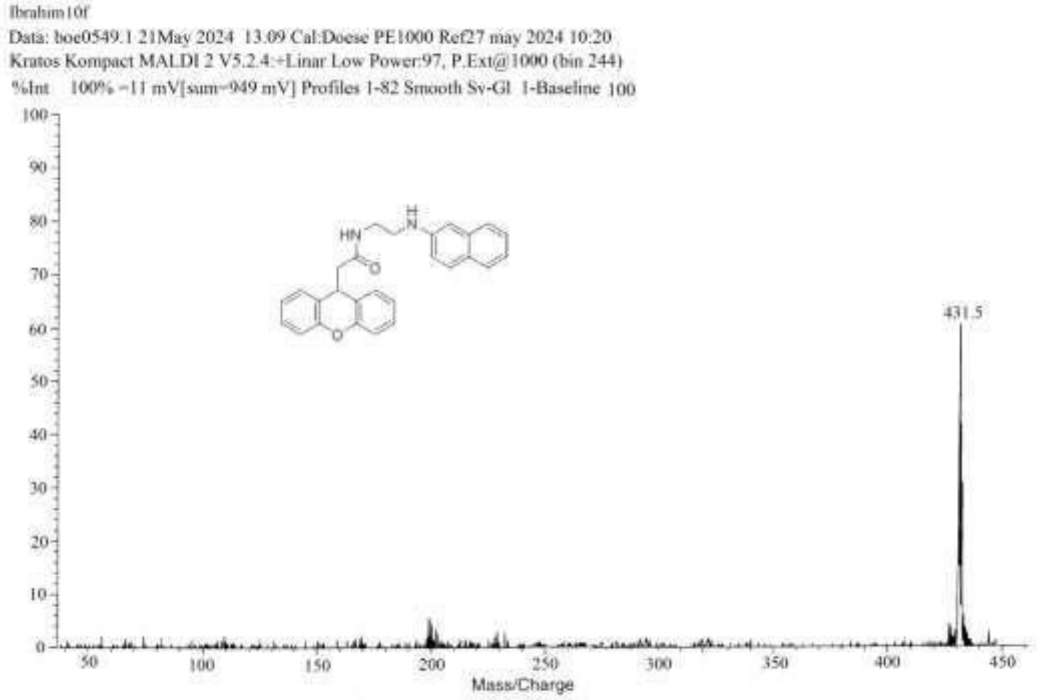

Figure S24. MALDI of compound **5h**.

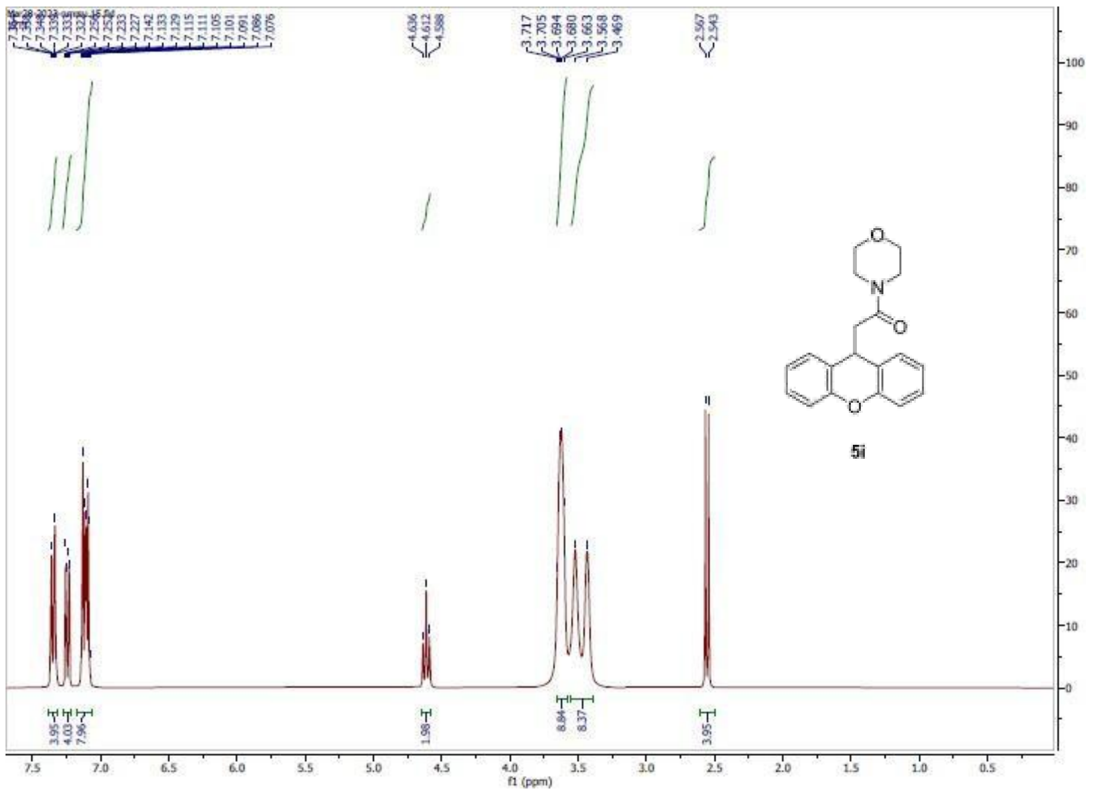

Figure S25. ^{1}H NMR of **5i**.

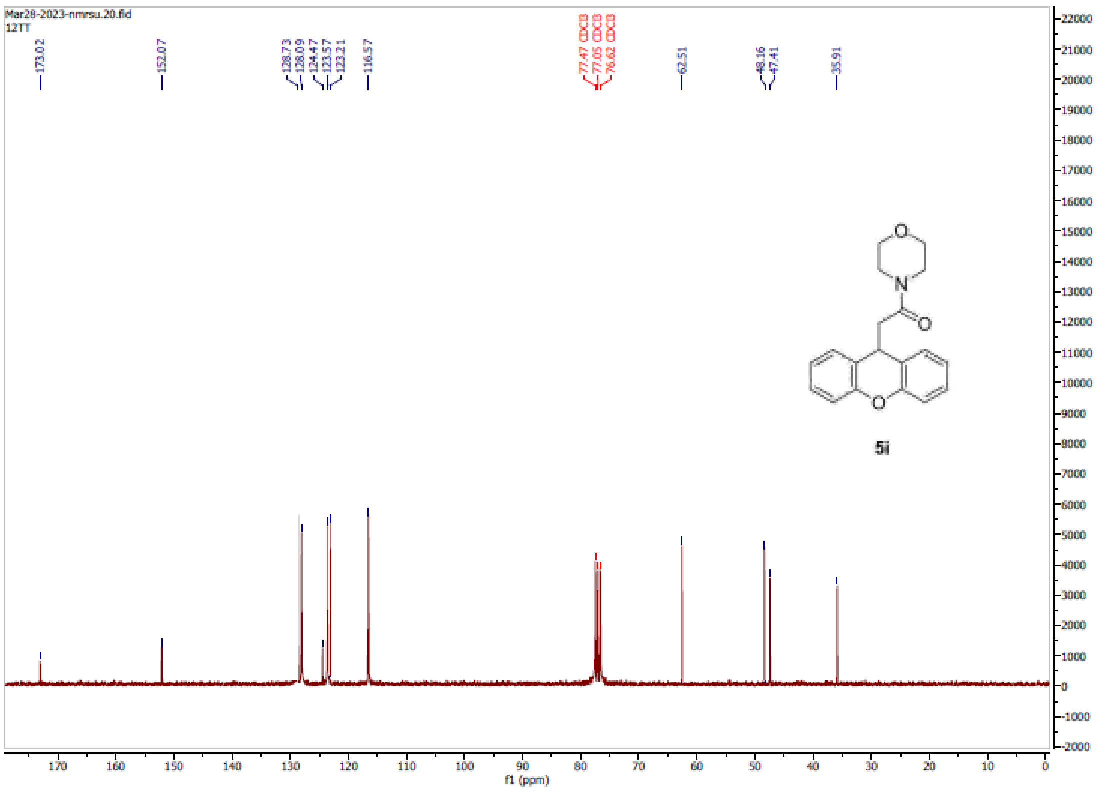

Figure S26. ^{13}C NMR of **5i**.

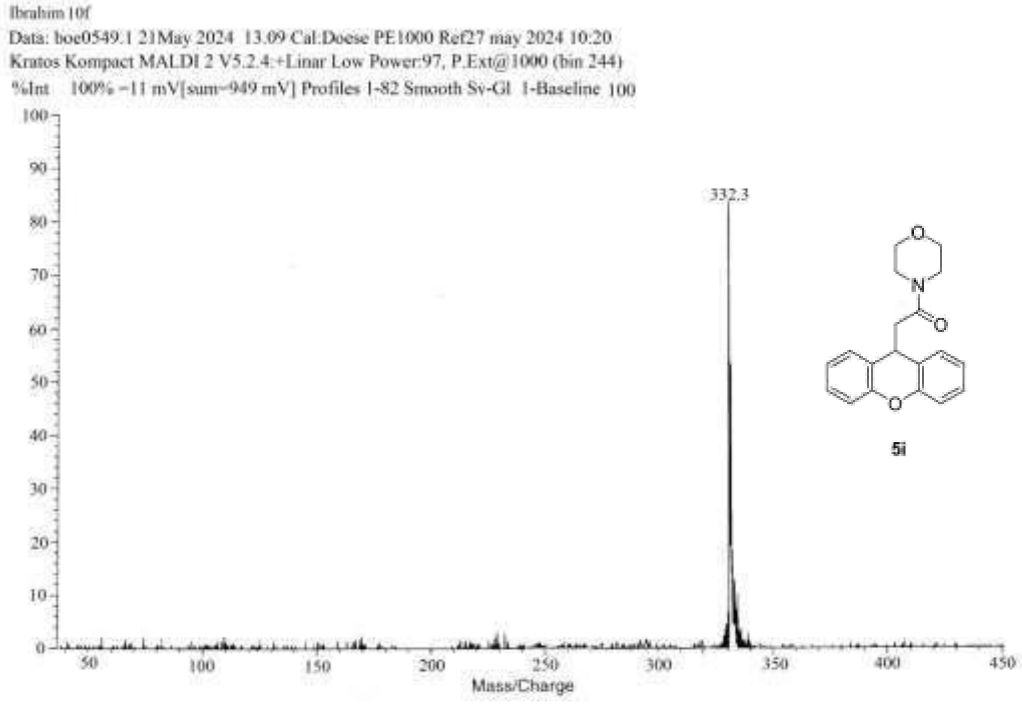

Figure S27. MALDI of compound **5i**.

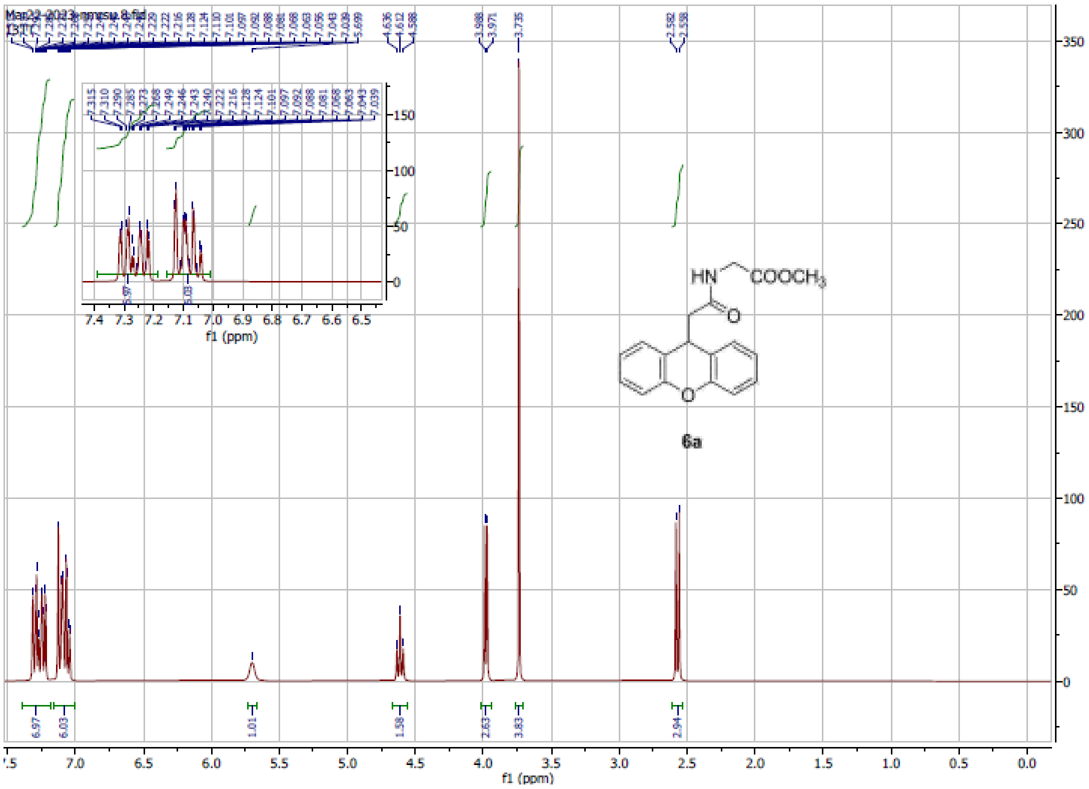

Figure S28. ^{1}H NMR of **6a**.

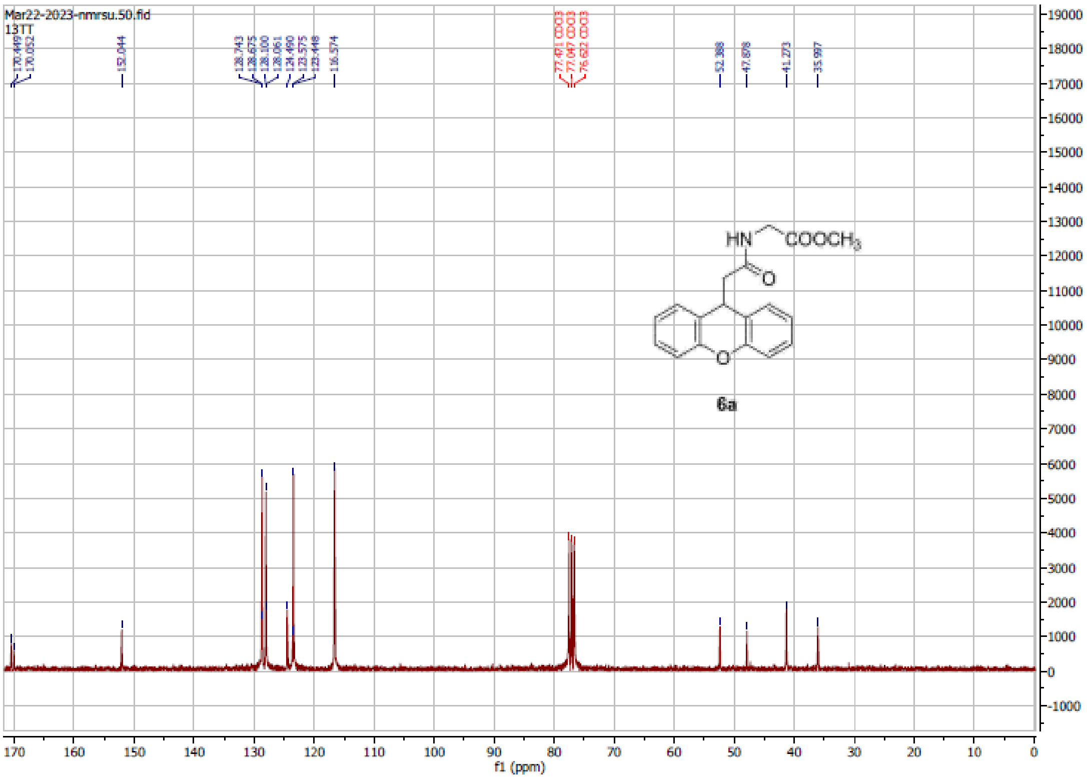

Figure S29. ^{13}C NMR of **6a**.

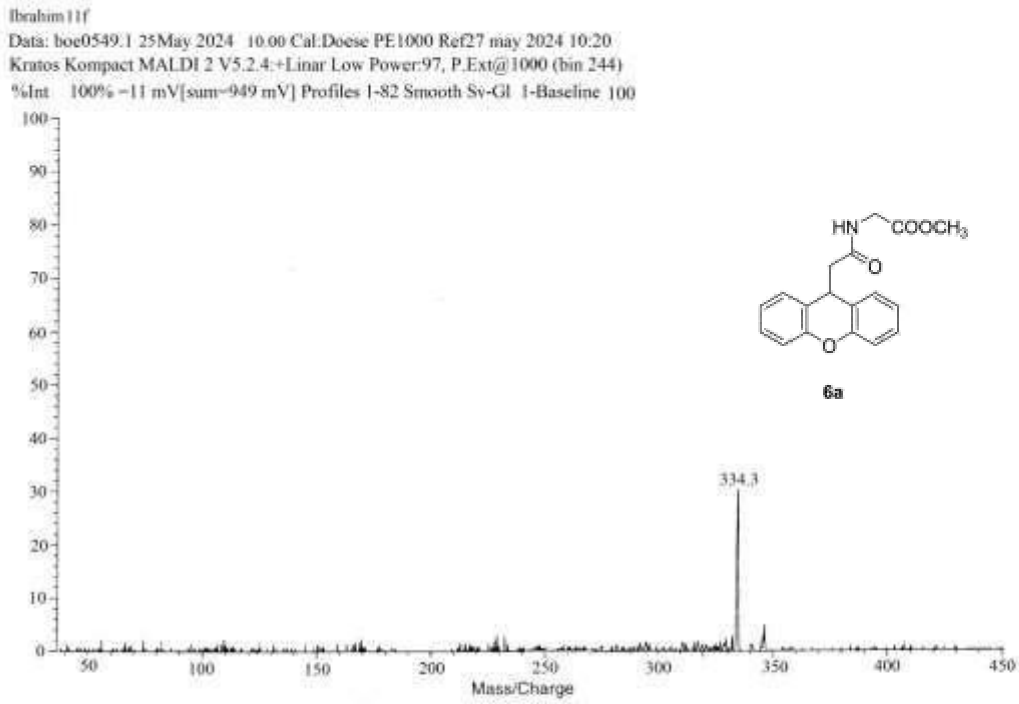

Figure S30. MALDI of compound **6a**.

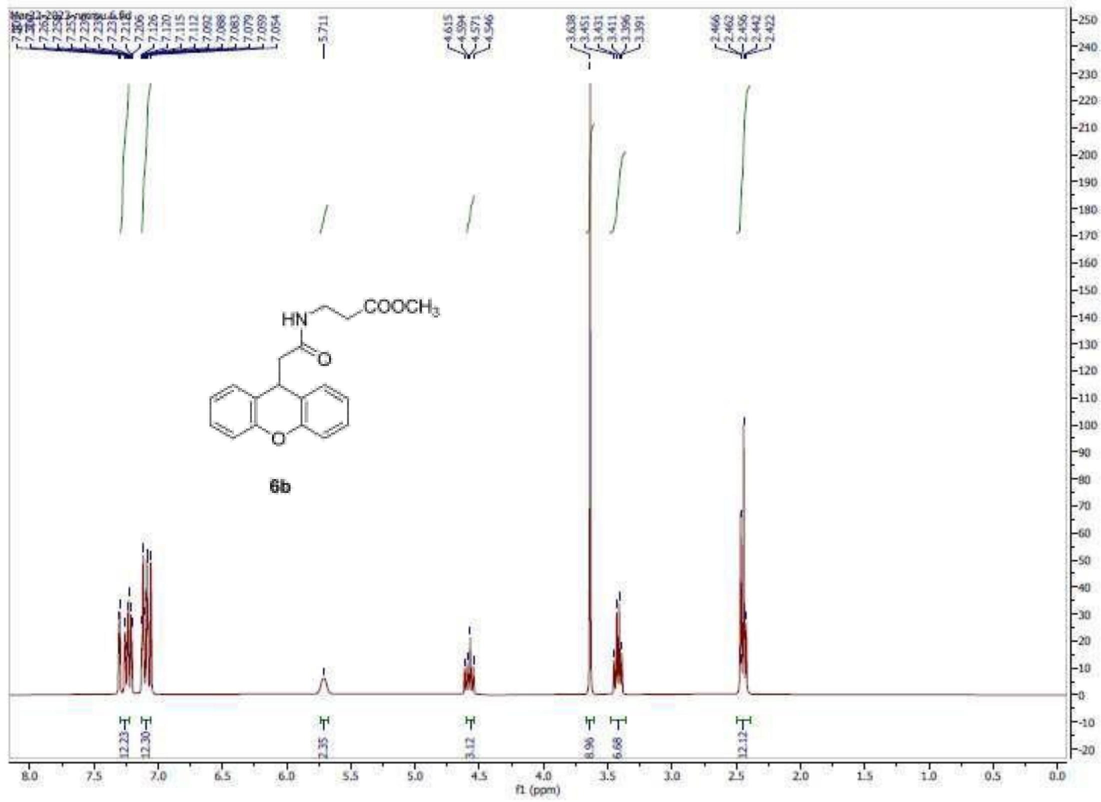

Figure S31. ^{1}H NMR of **6b**.

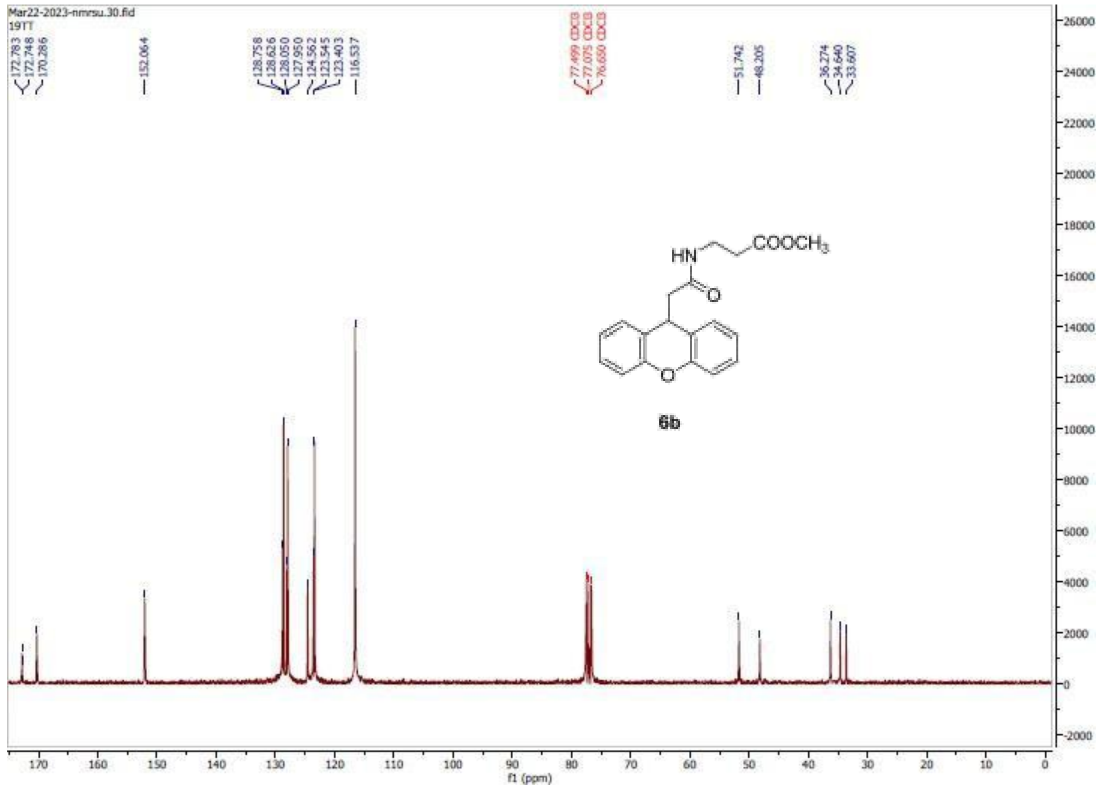

Figure S32. ^{13}C NMR of **6b**.

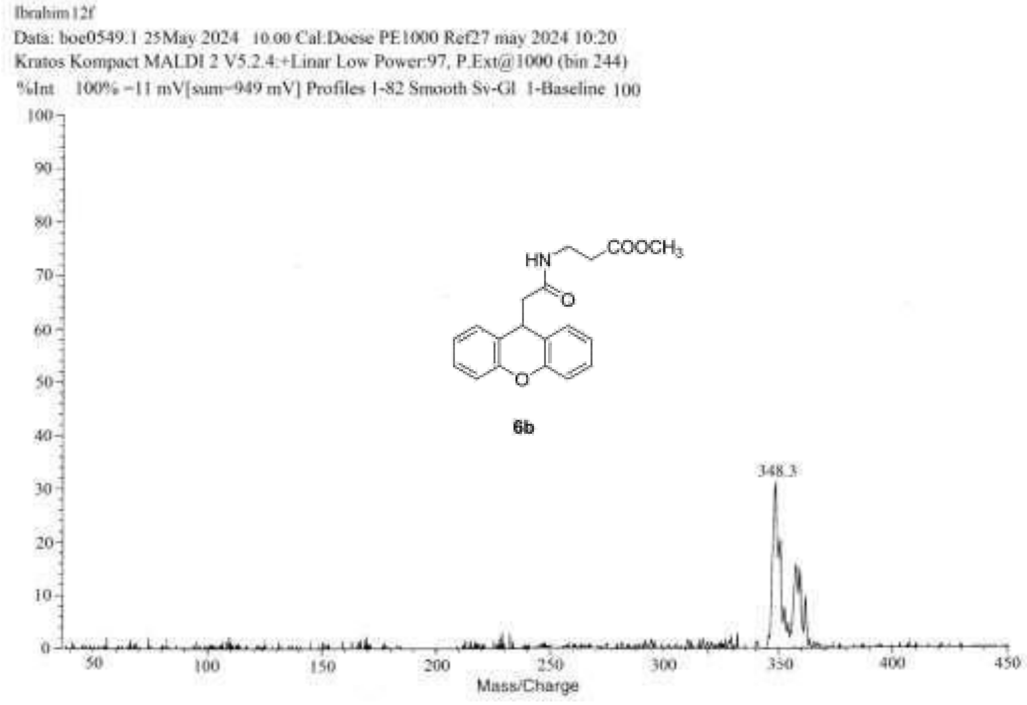

Figure S33. MALDI of compound **6b**.

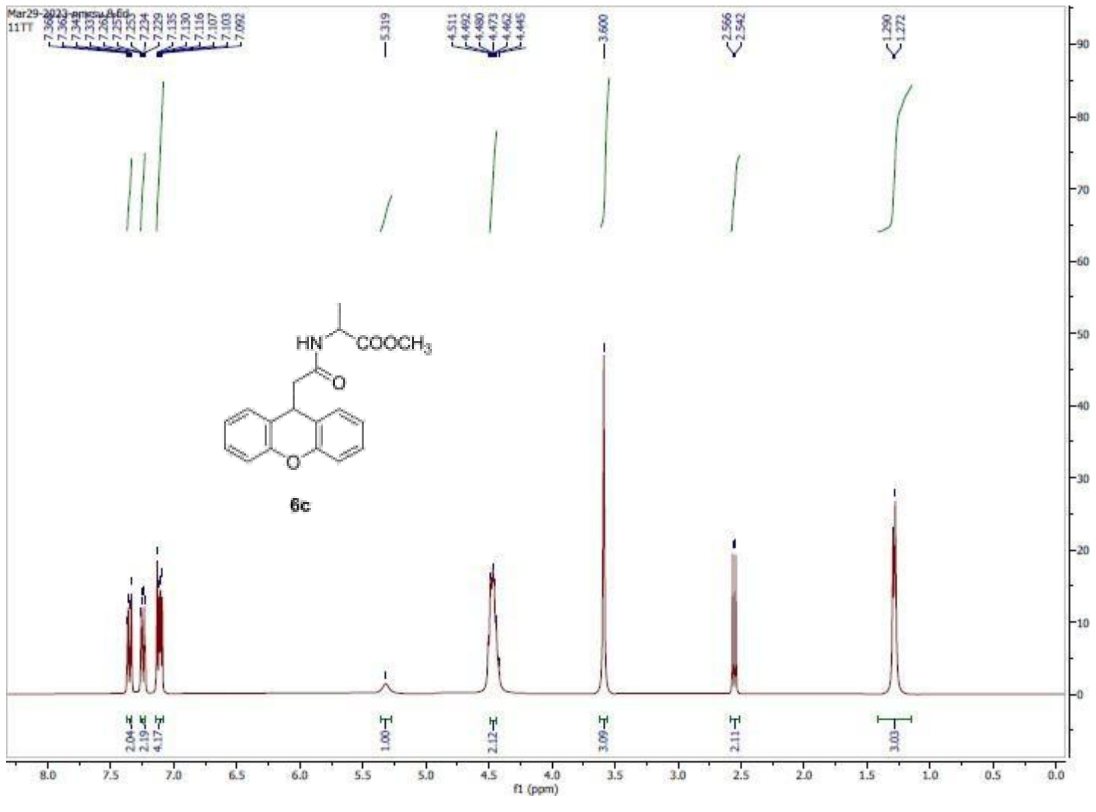

Figure S34. ^{1}H NMR of **6c**.

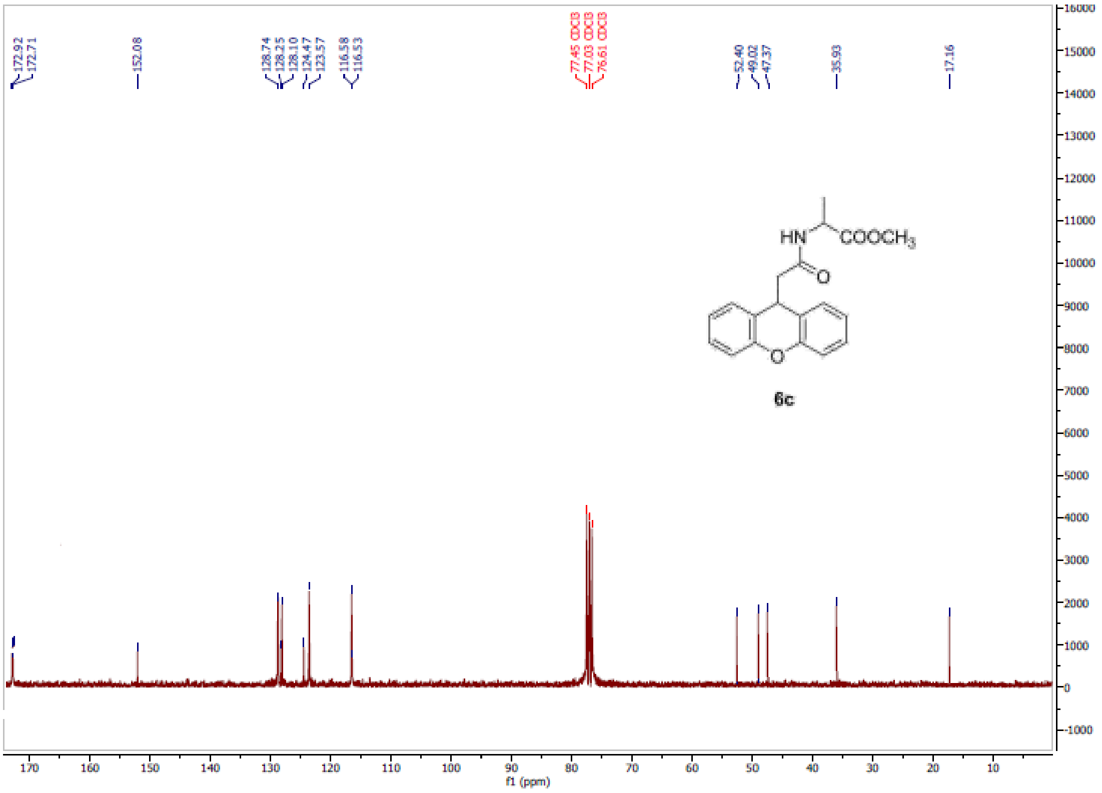

Figure S35. ^{13}C NMR of **6c**.

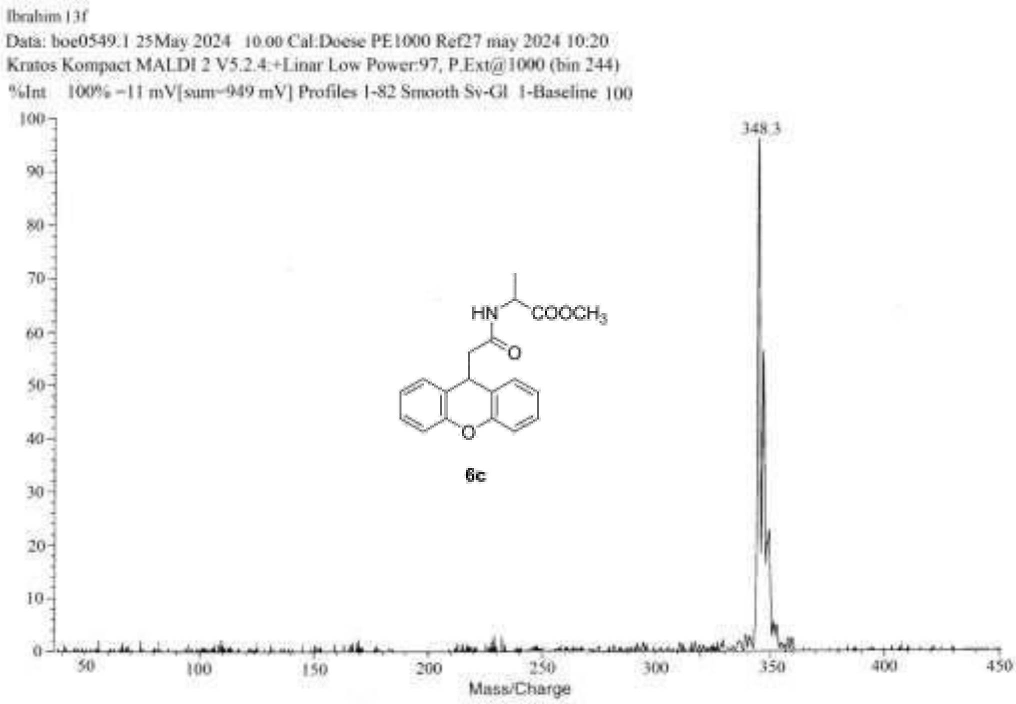

Figure S36. MALDI of compound **6c**.

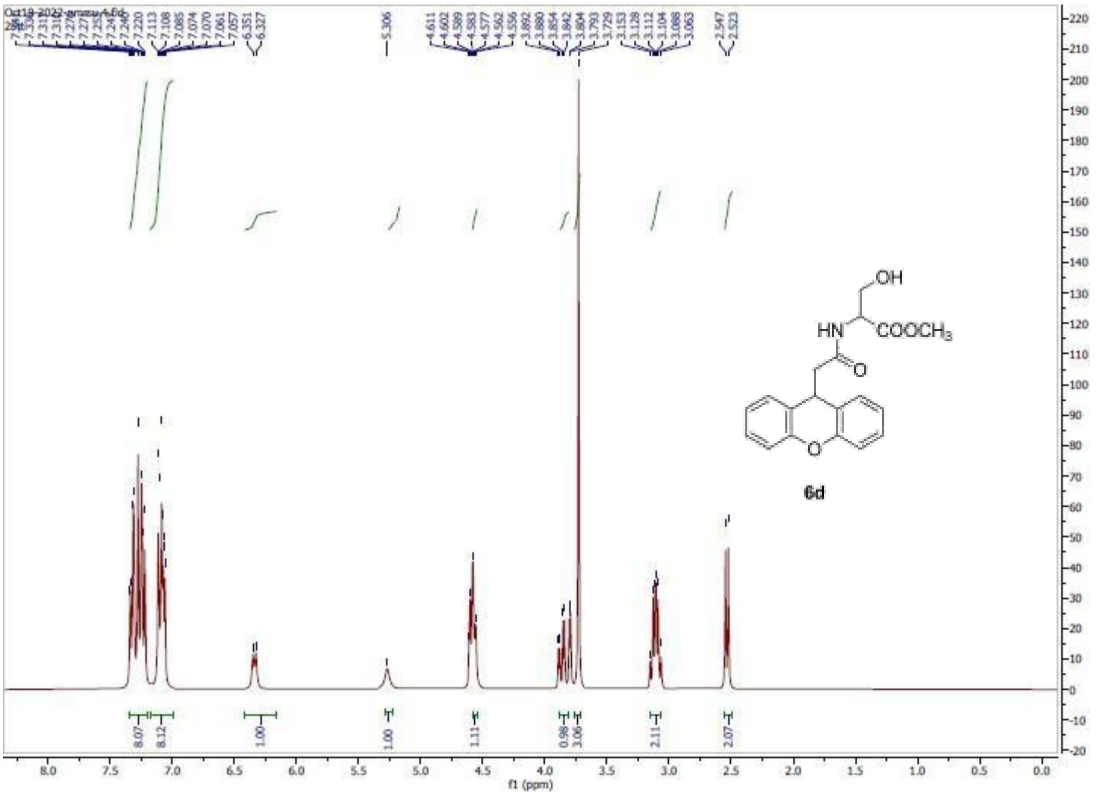

Figure S37. ^{1}H NMR of **6d**.

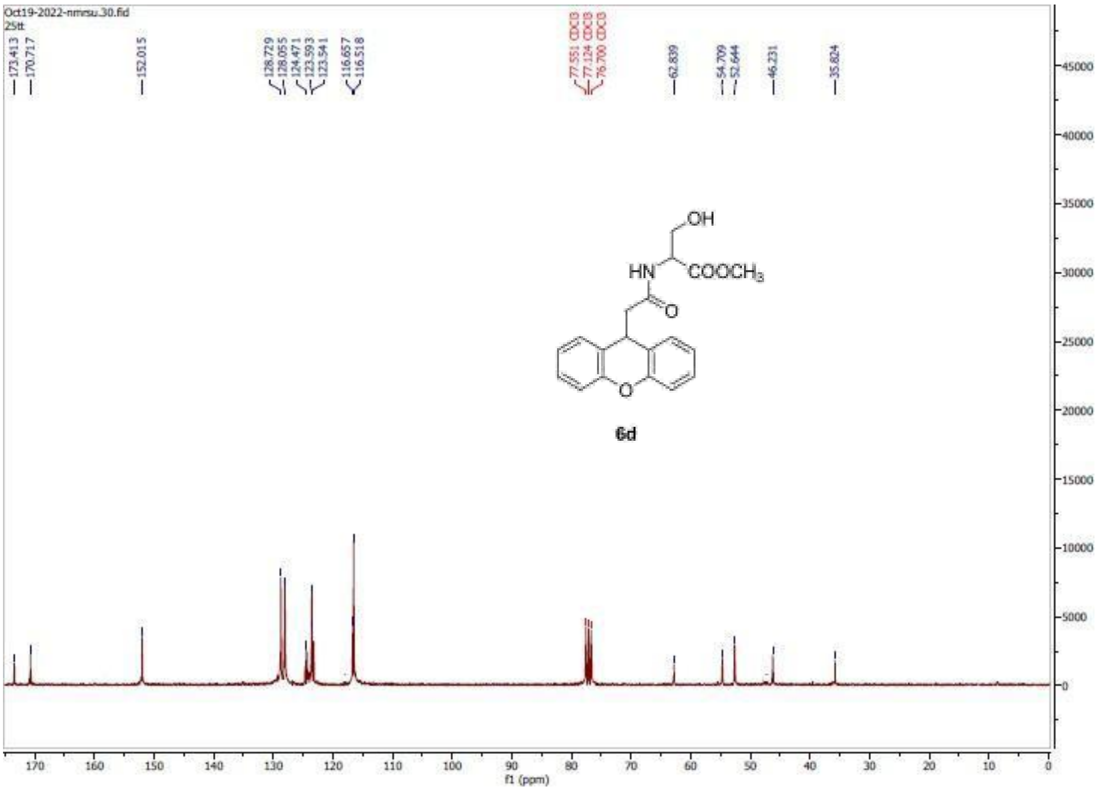

Figure S38. ^{13}C NMR of **6d**.

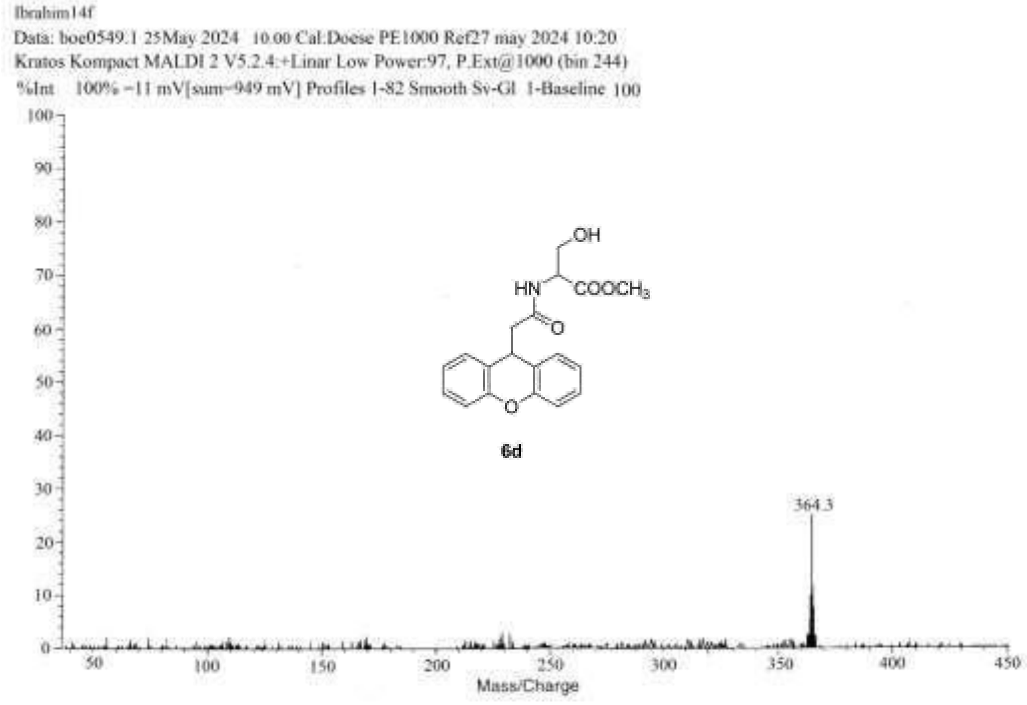

Figure S39. MALDI of compound **6d**.

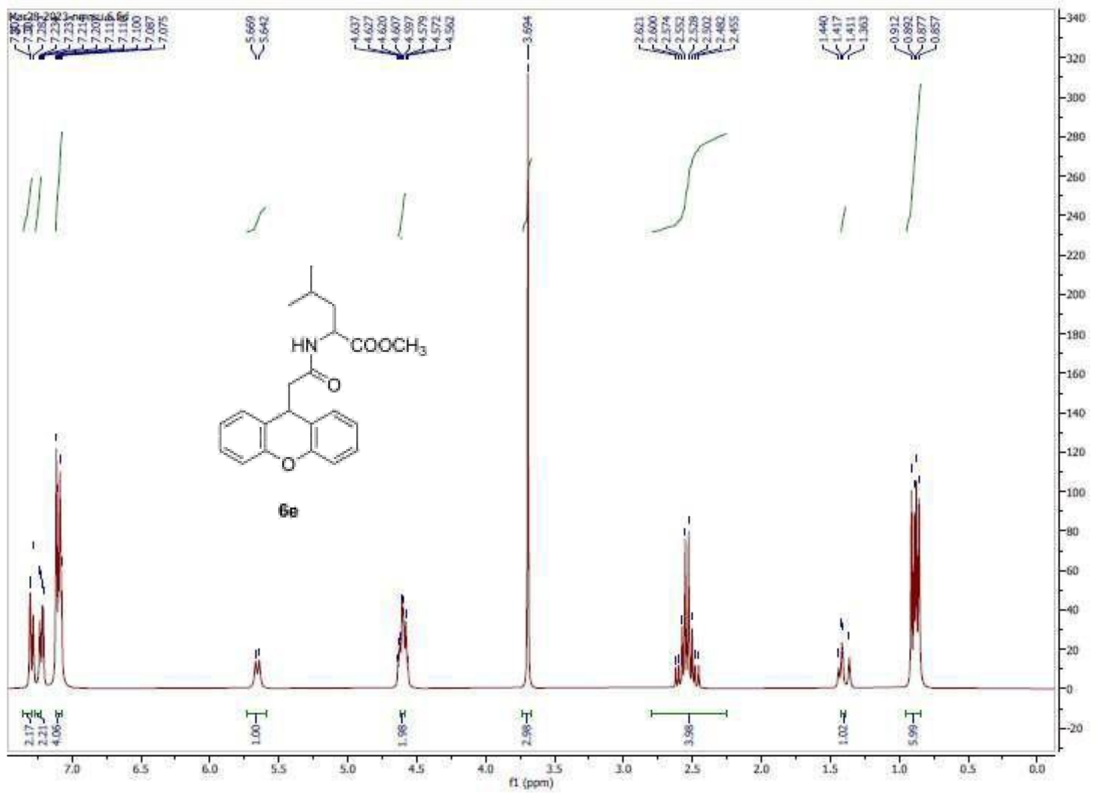

Figure S40. ^{1}H NMR of **6e**.

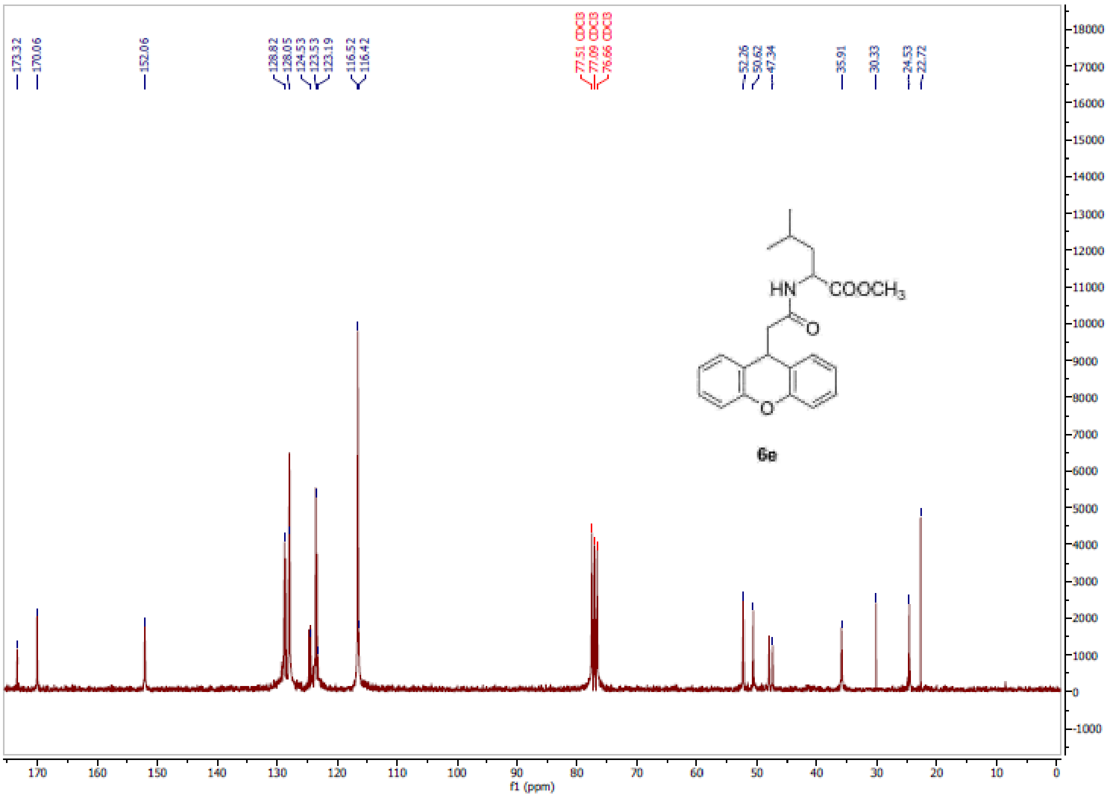

Figure S41. ^{13}C NMR of **6e**.

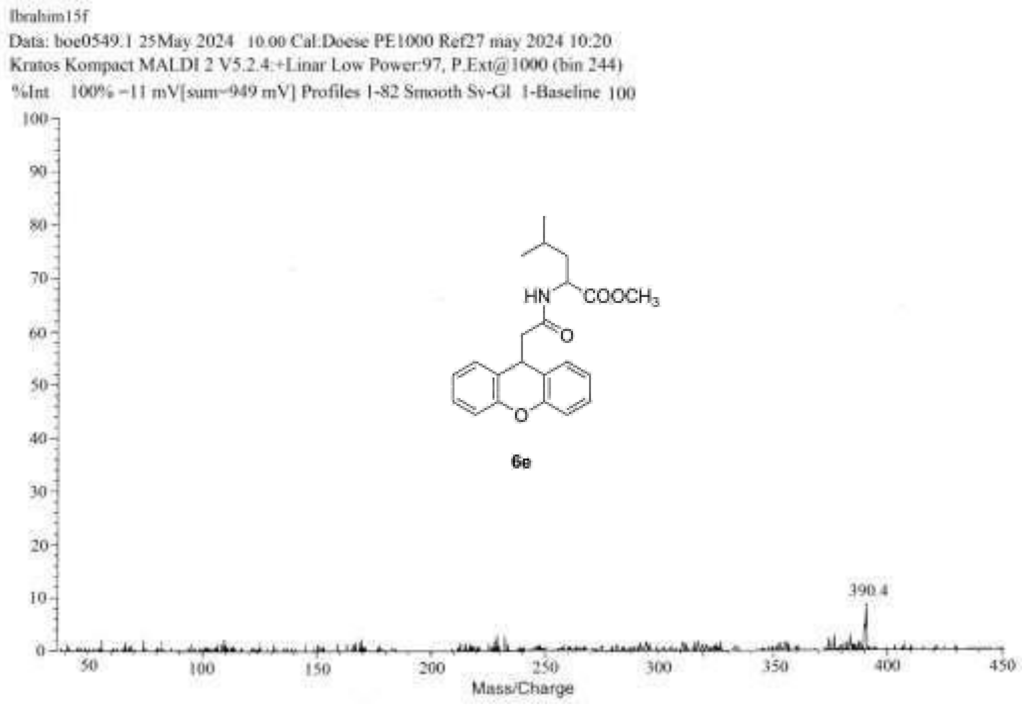

Figure S42. MALDI of compound **6e**.

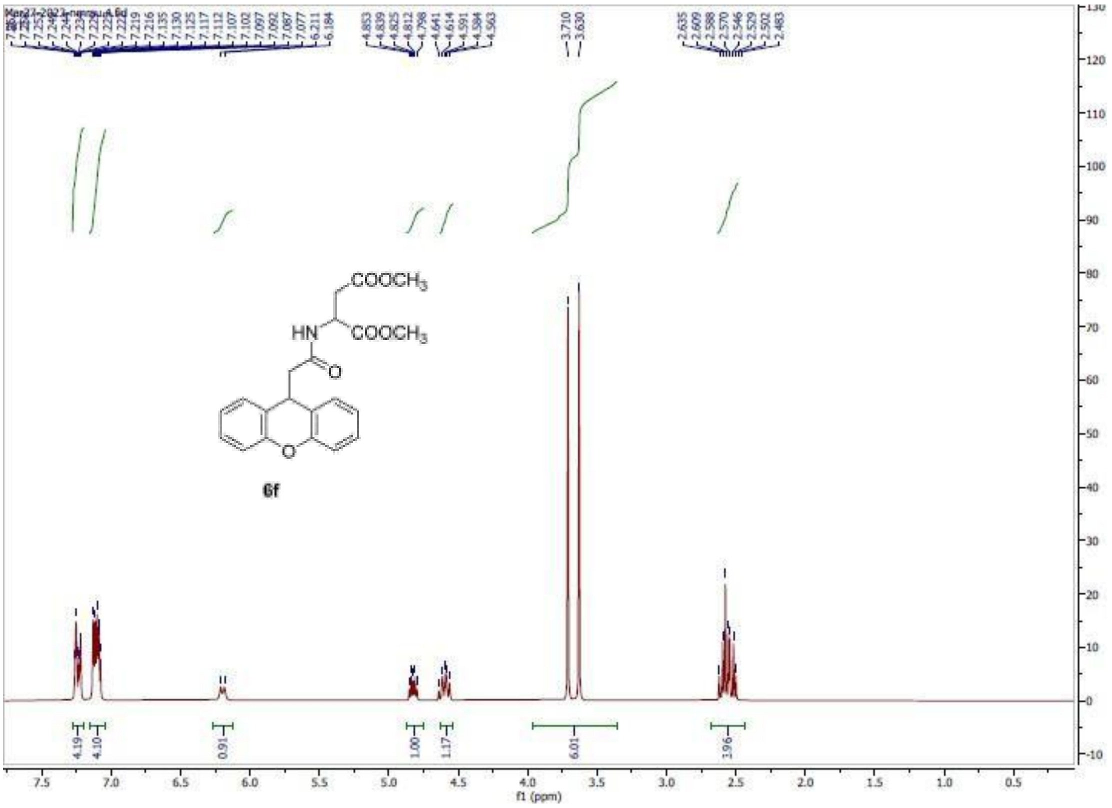

Figure S43. ^{1}H NMR of **6f**.

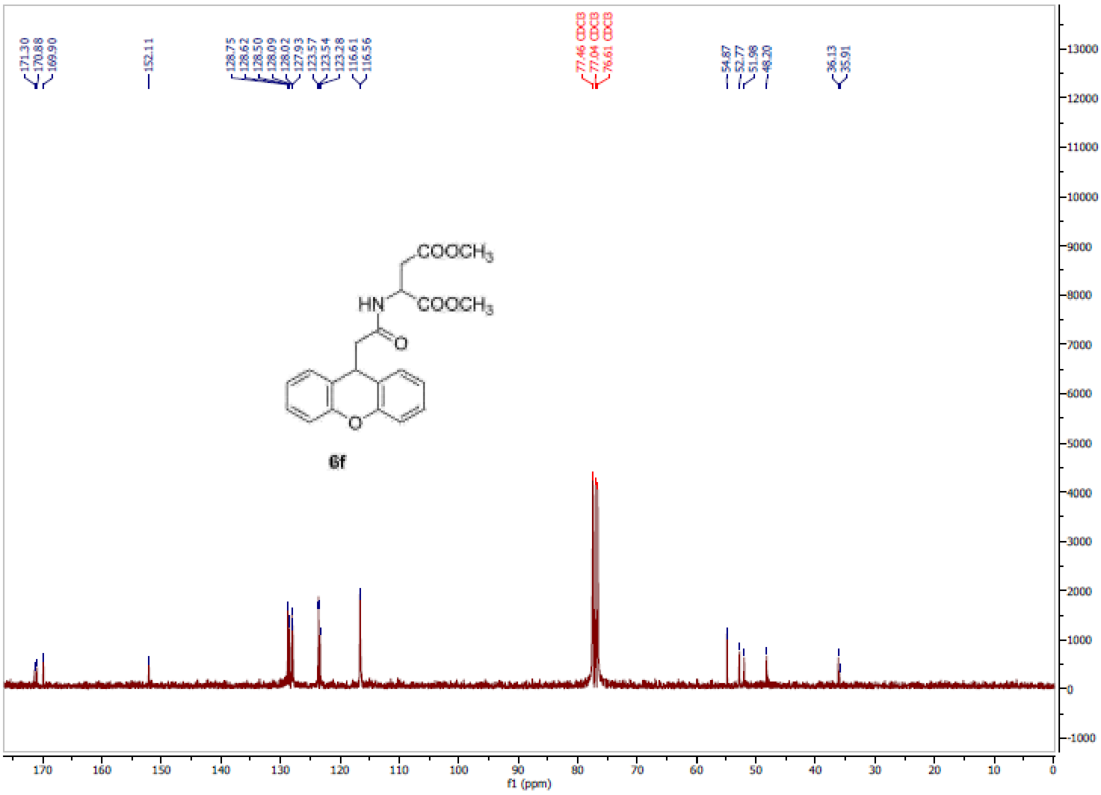

Figure S44. ^{13}C NMR of **6f**.

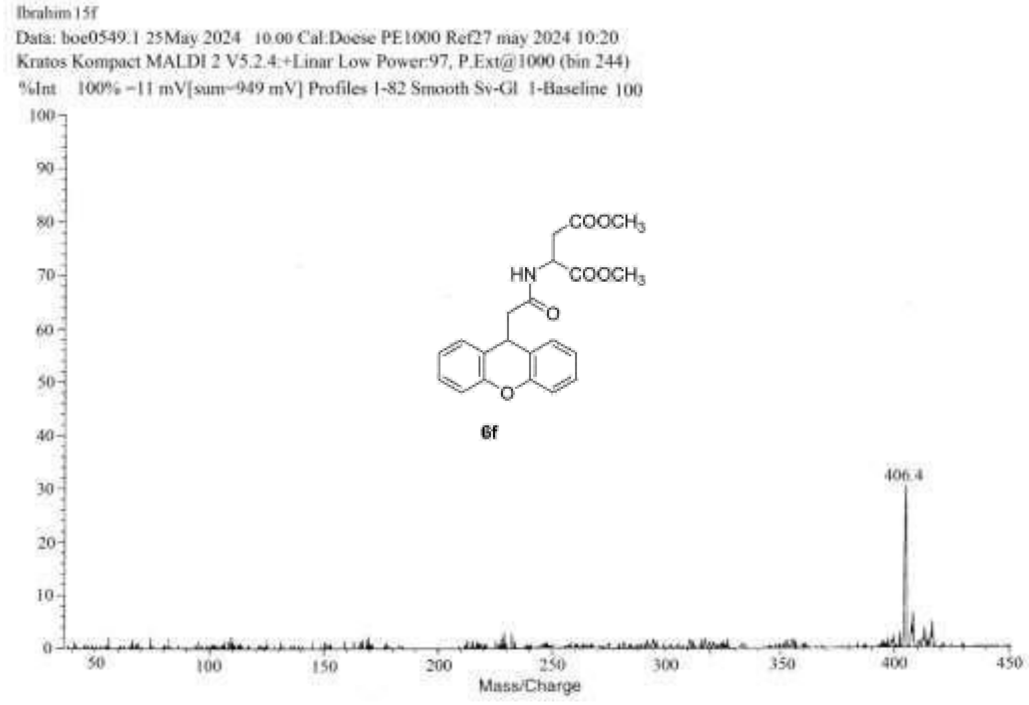

Figure S45. MALDI of compound **6f**.

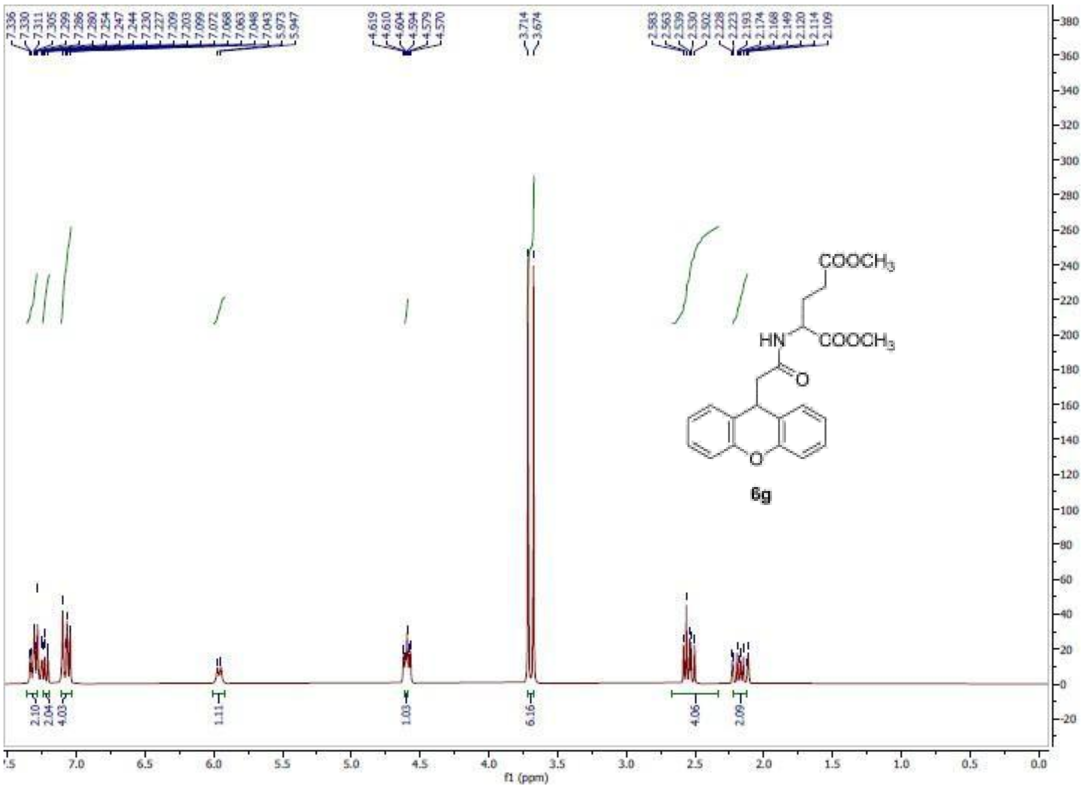

Figure S46. ^{1}H NMR of **6g**.

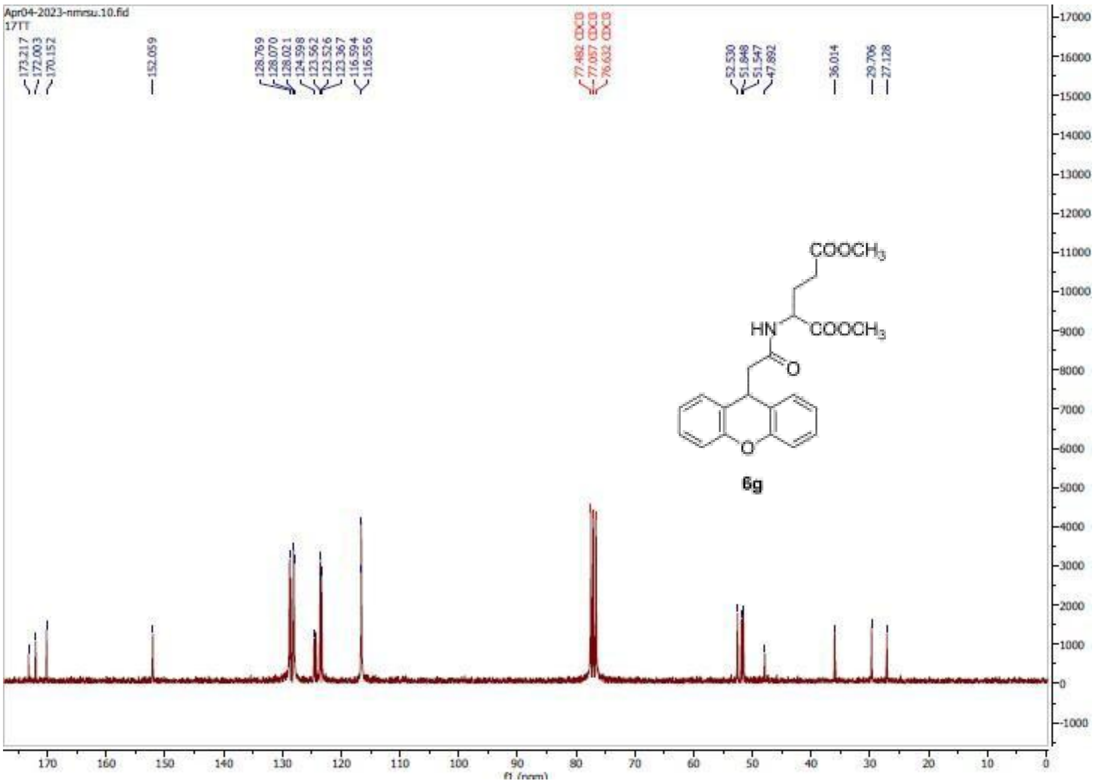

Figure S47. ^{13}C NMR of **6g**.

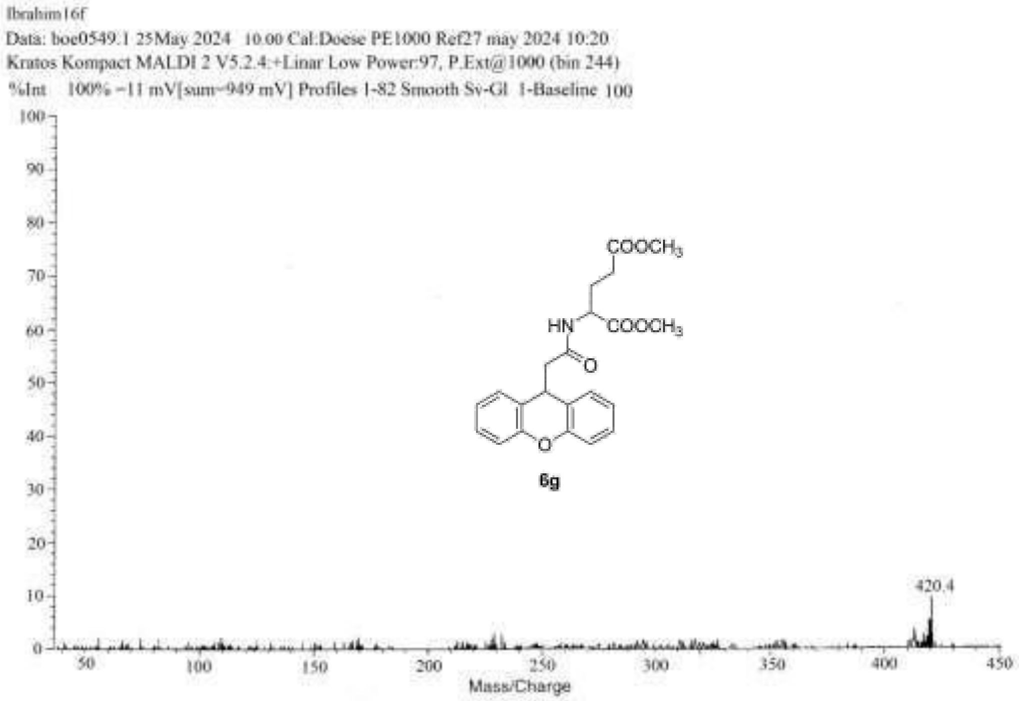

Figure S48. MALDI of compound **6g**.

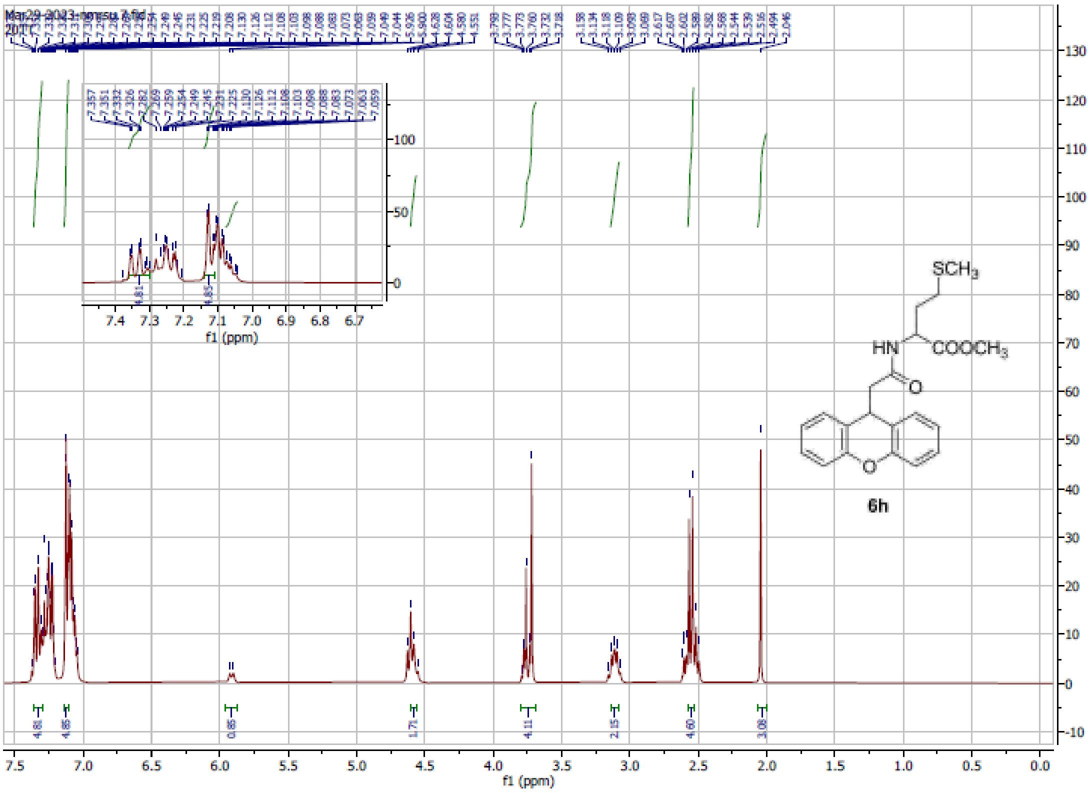

Figure S49. ^{1}H NMR of **6h**.

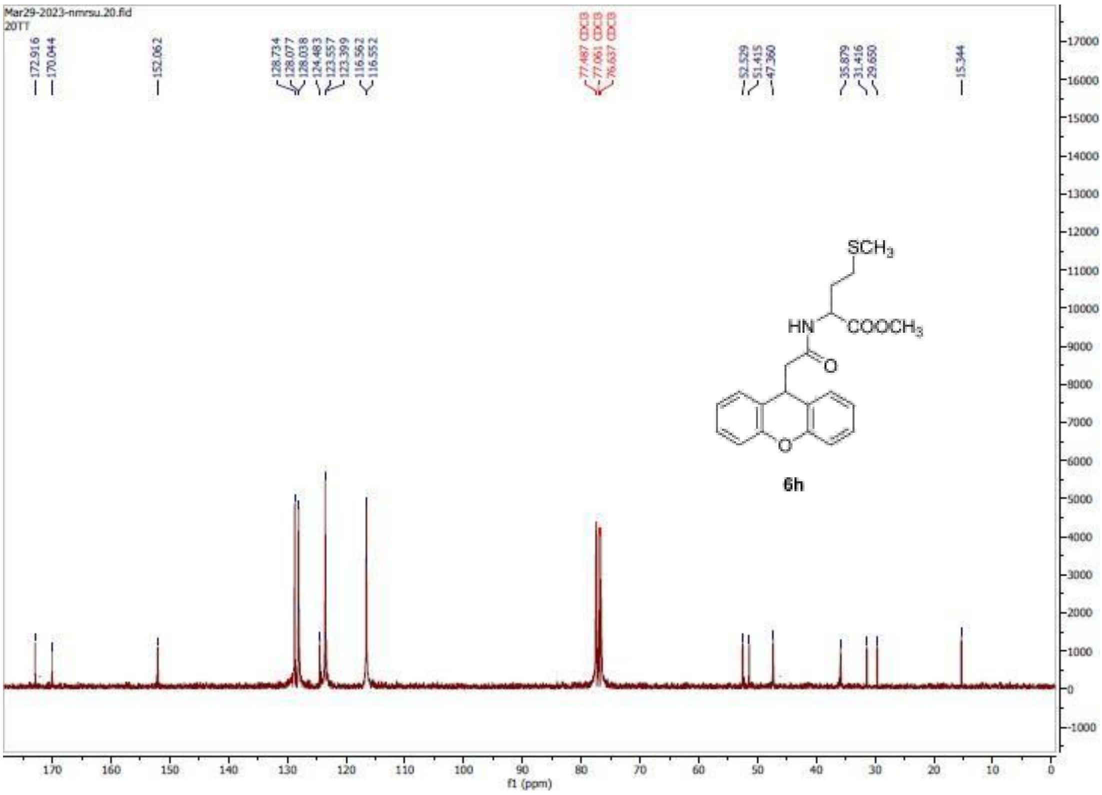

Figure S50. ^{13}C NMR of **6h**.

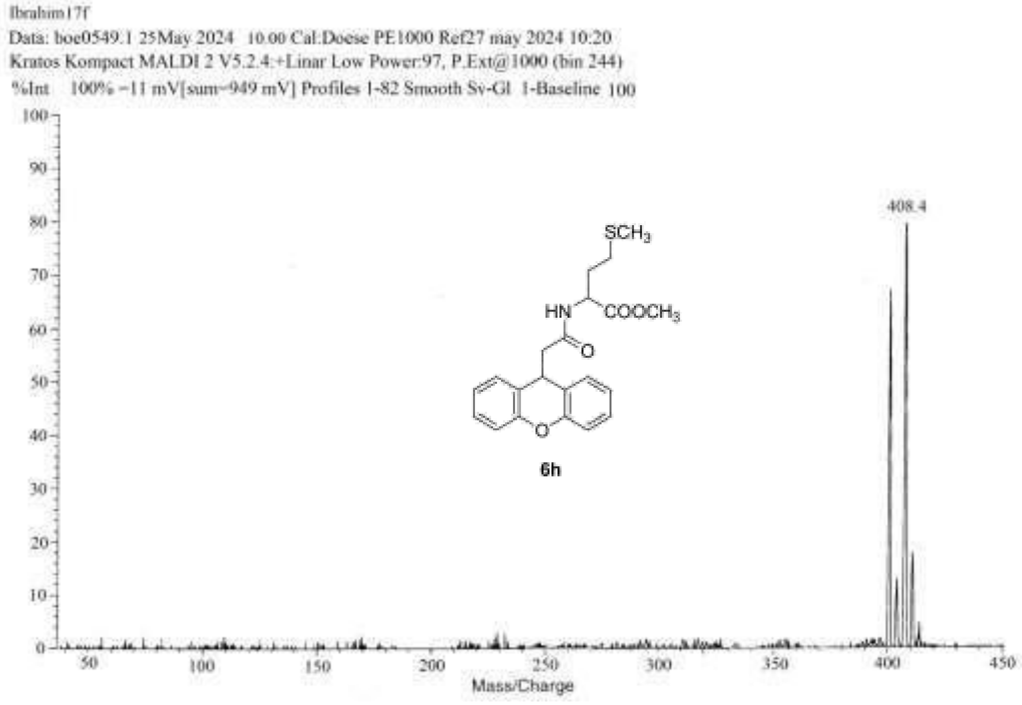

Figure S51. MALDI of compound **6h**.

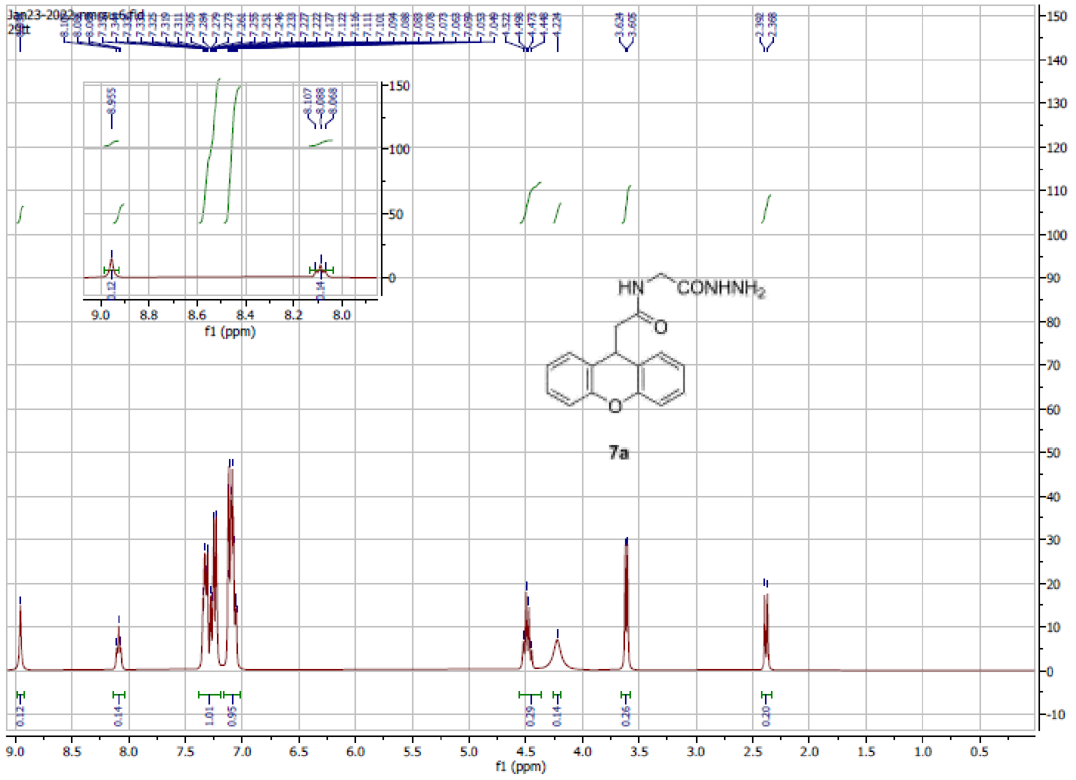

Figure S52. ^{1}H NMR of **7a**.

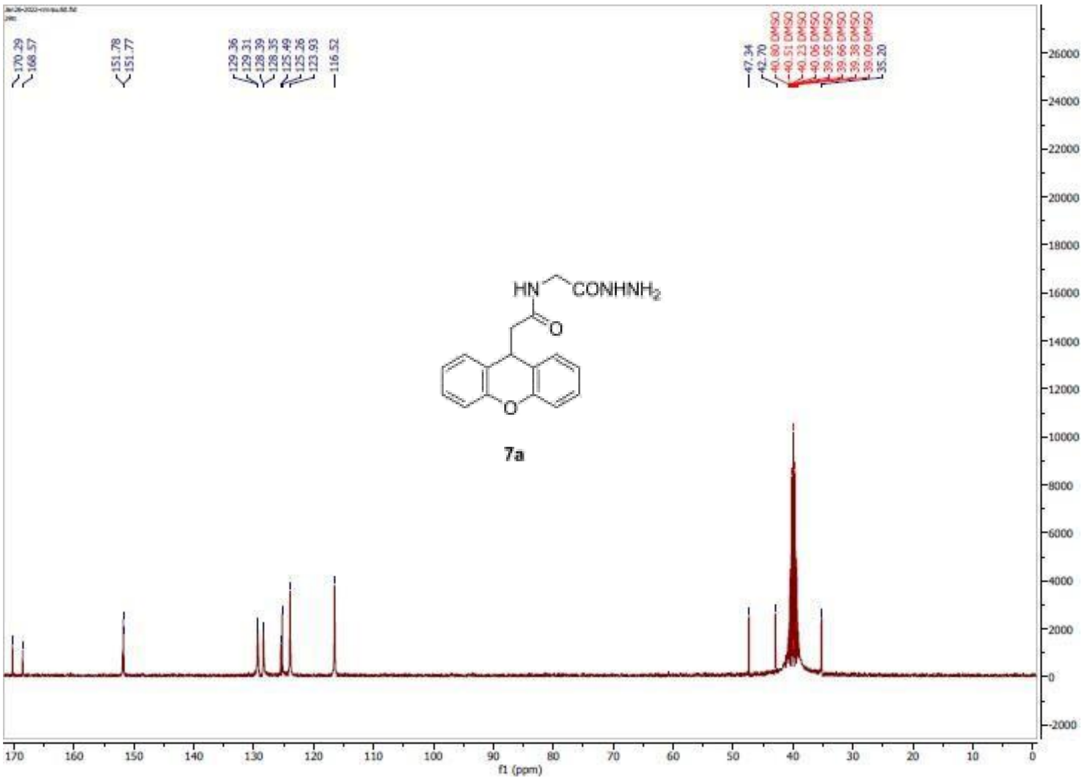

Figure S53. ^{13}C NMR of **7a**.

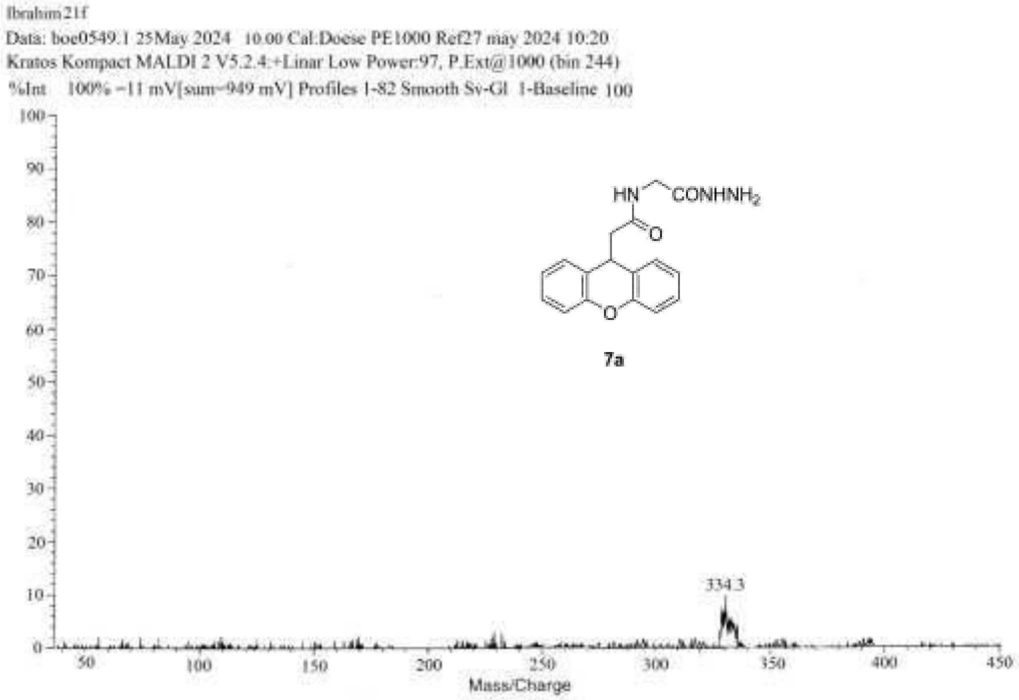

Figure S54. MALDI of compound **7a**.

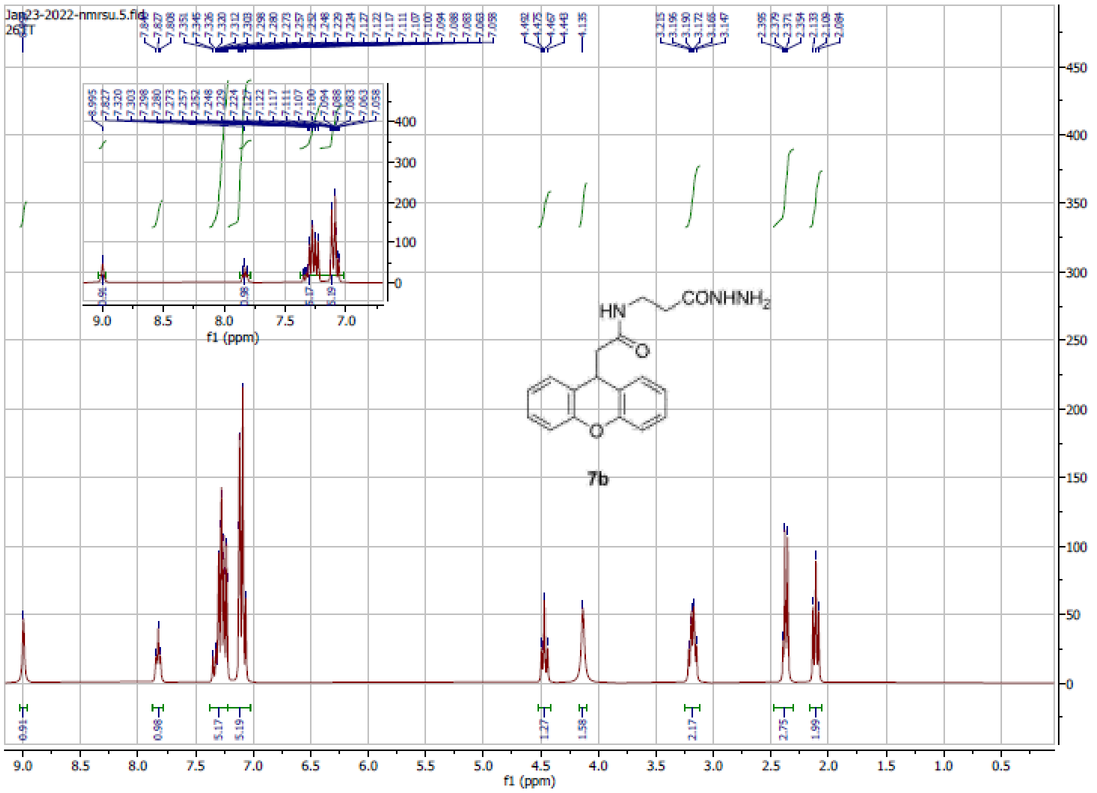

Figure S55. ^{1}H NMR of **7b**.

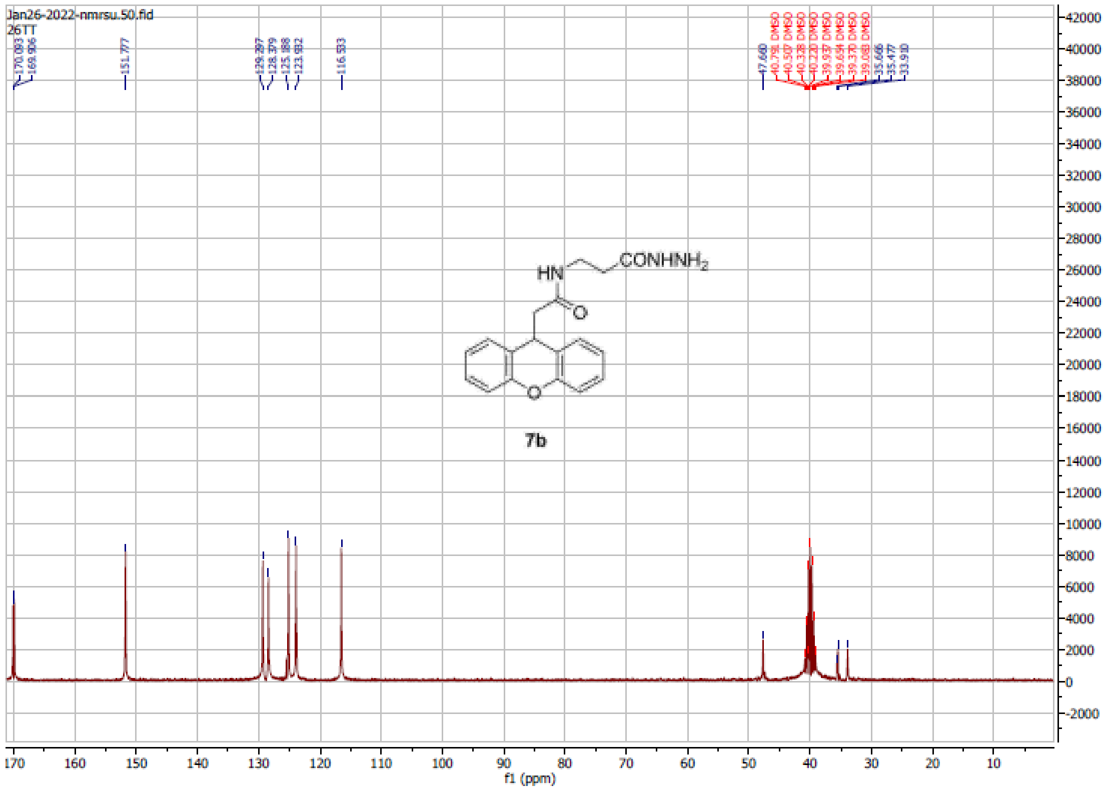

Figure S56. ^{13}C NMR of **7b**.

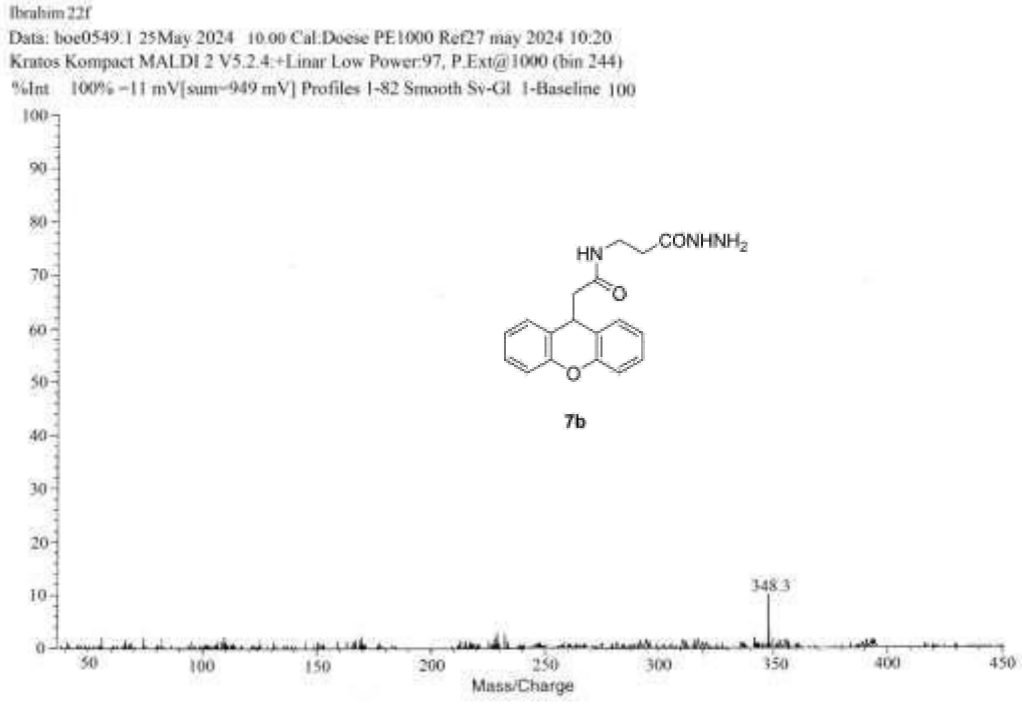

Figure S57. MALDI of compound **7b**.

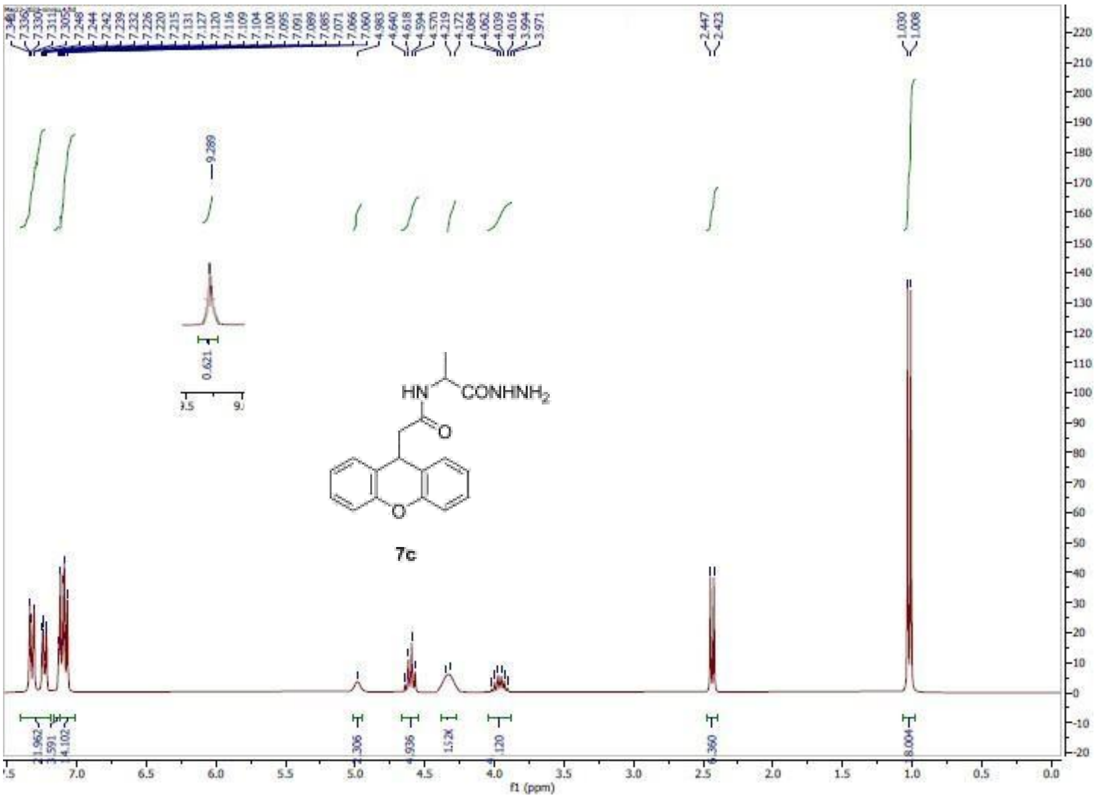

Figure S58. ^{1}H NMR of **7c**.

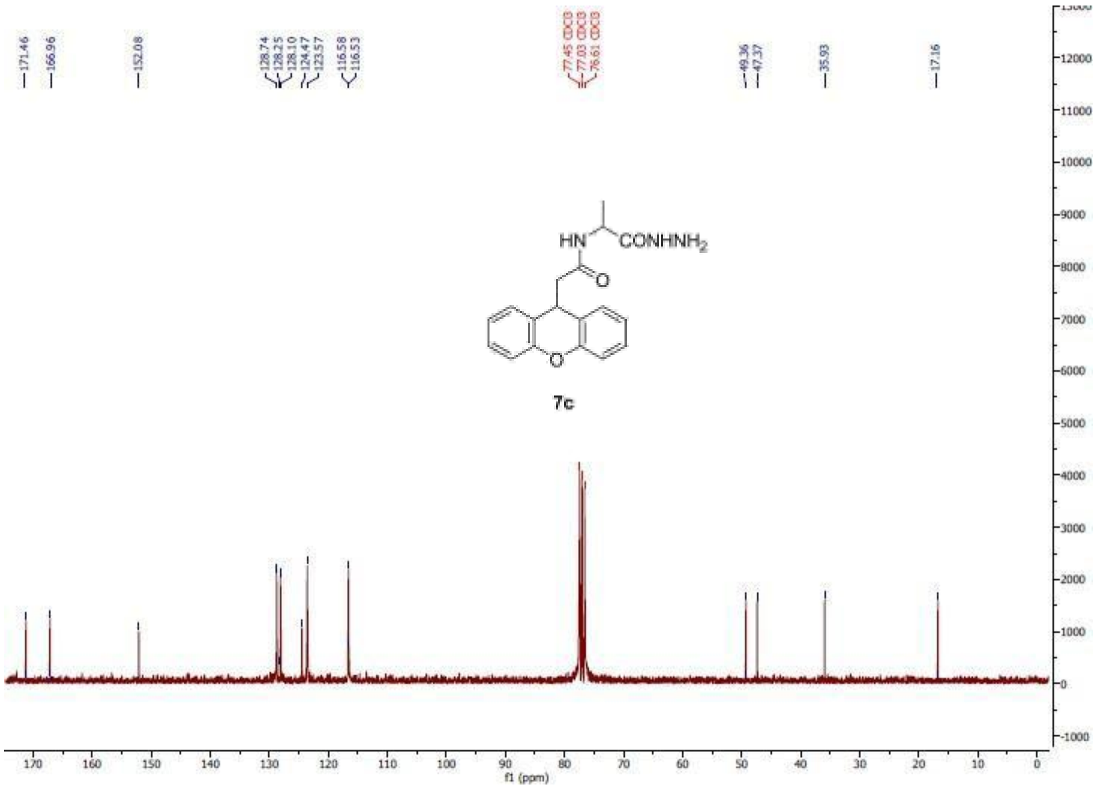

Figure S59. ^{13}C NMR of **7c**.

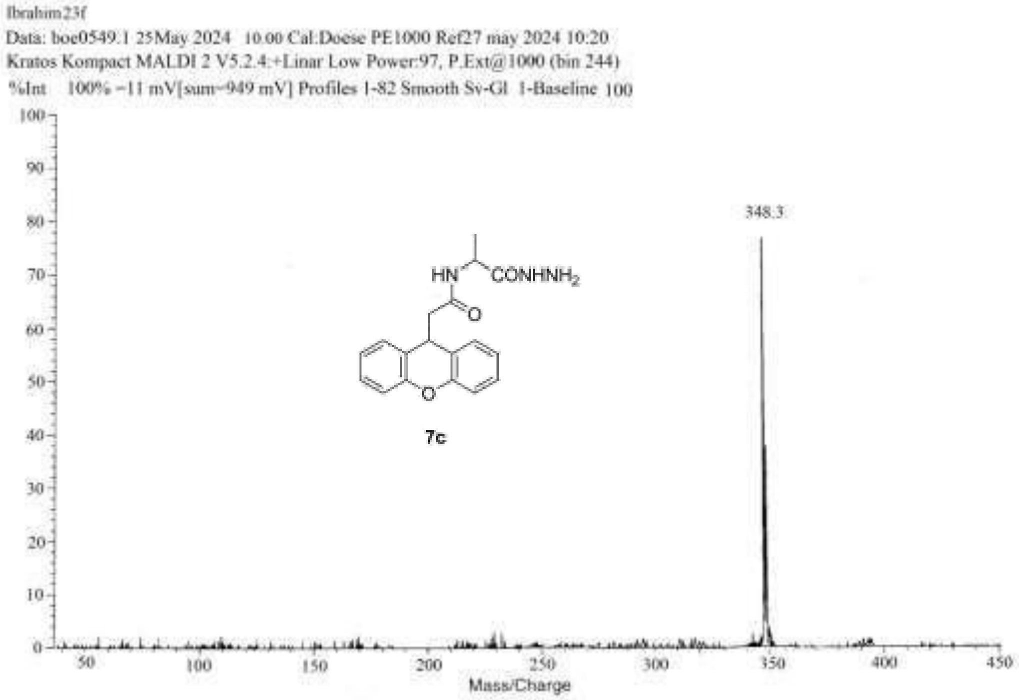

Figure S60. MALDI of compound **7c**.

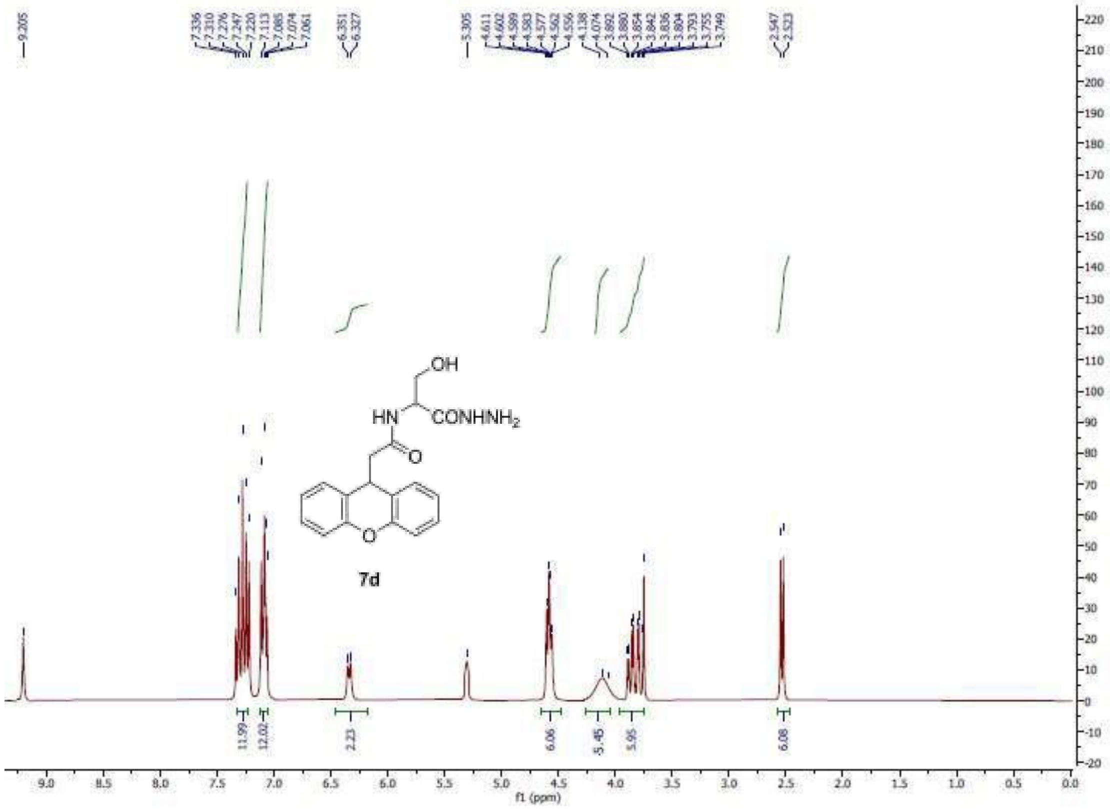

Figure S61. ^{1}H NMR of **7d**.

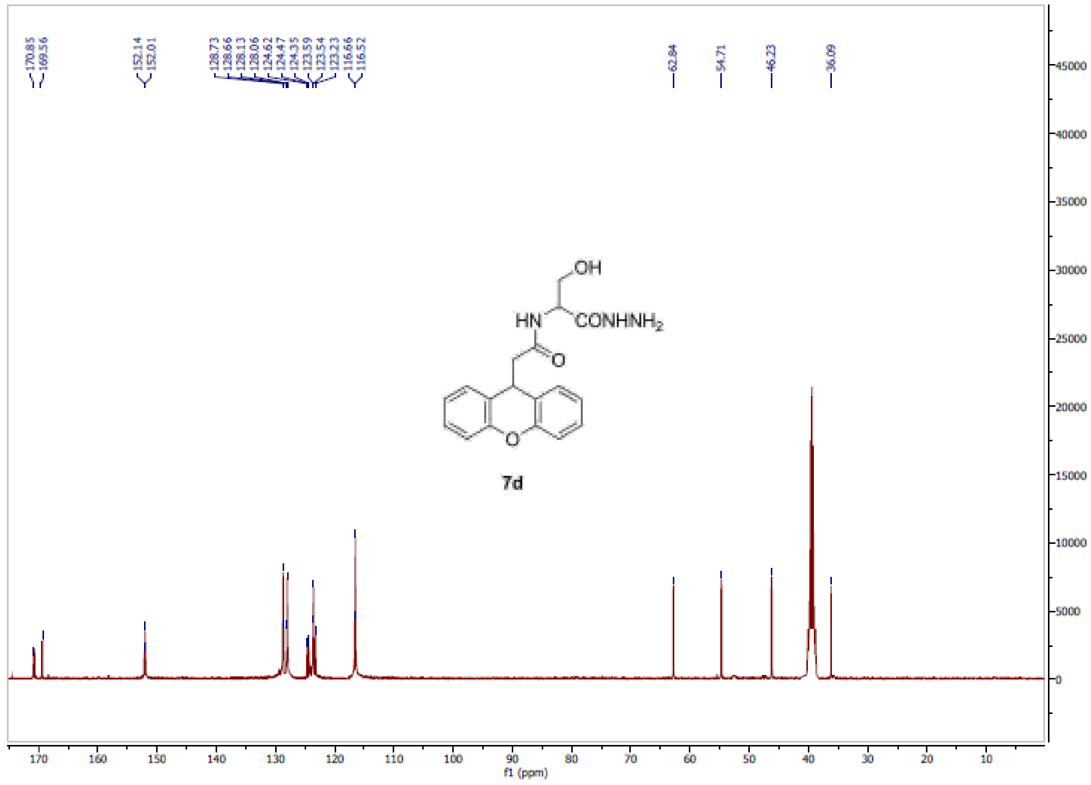

Figure S62. ^{13}C NMR of **7d**.

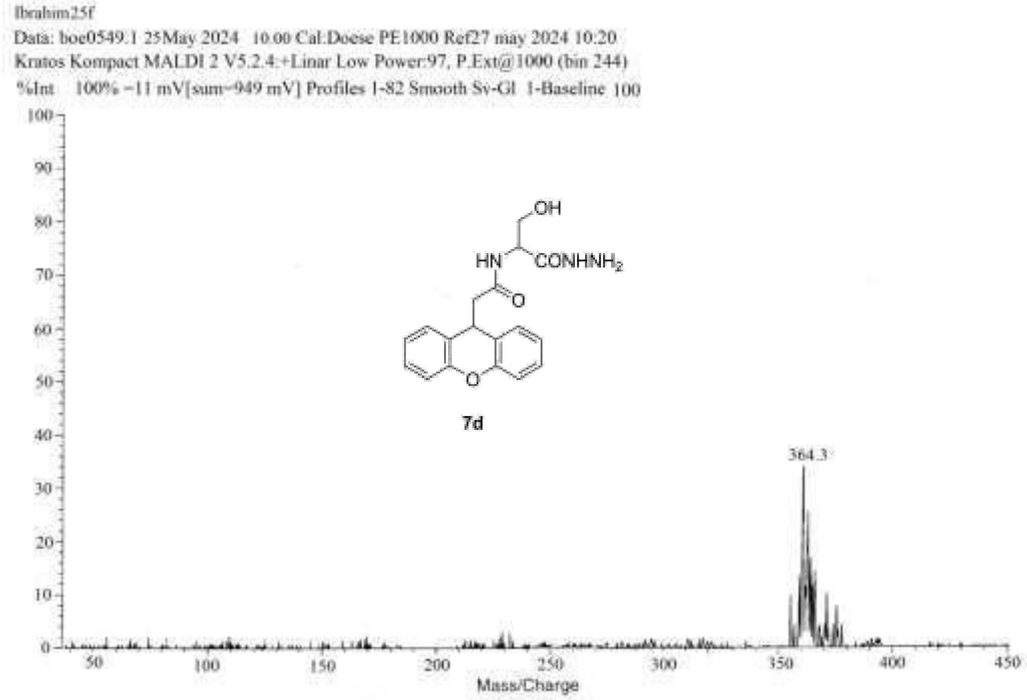

Figure S63. MALDI of compound **7d**.

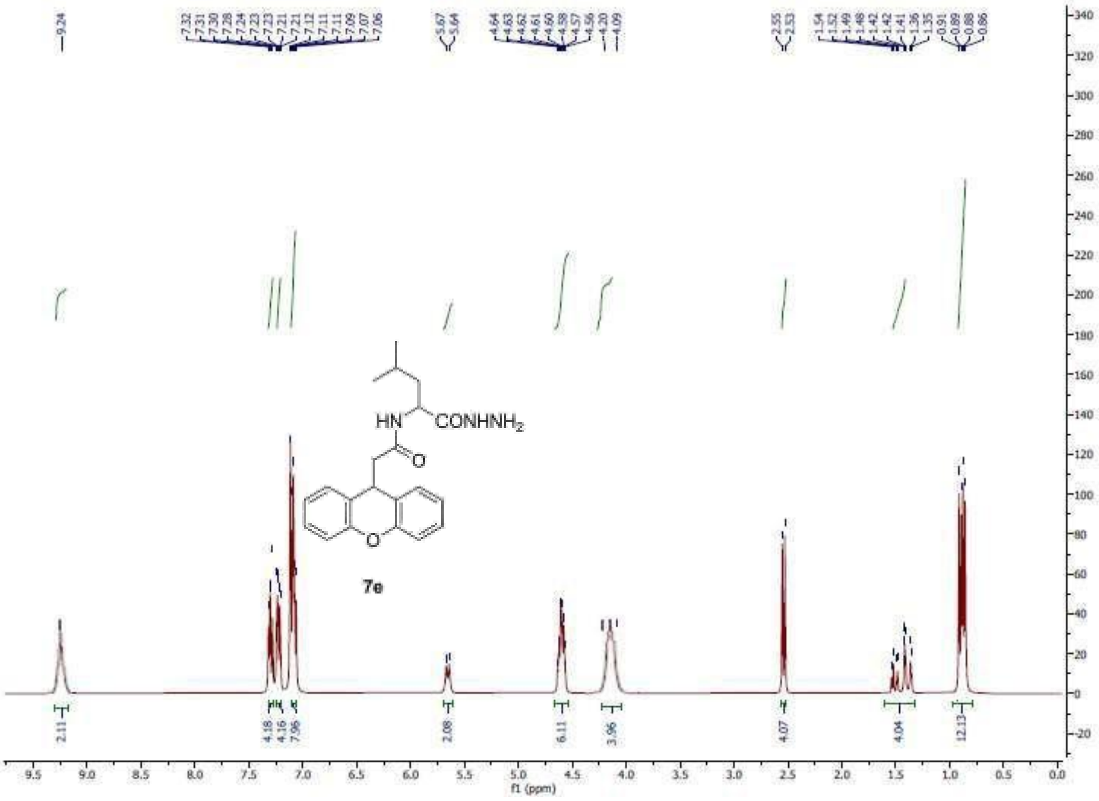

Figure S64. ^{1}H NMR of **7e**.

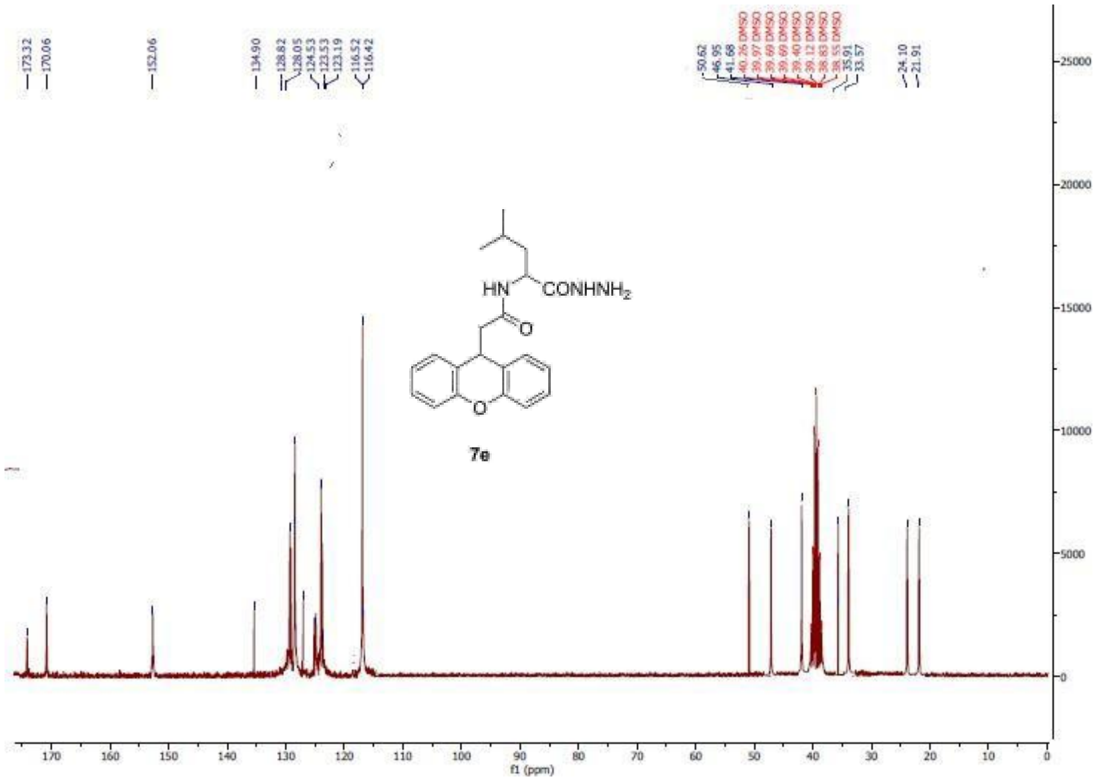

Figure S65. ^{13}C NMR of **7e**.

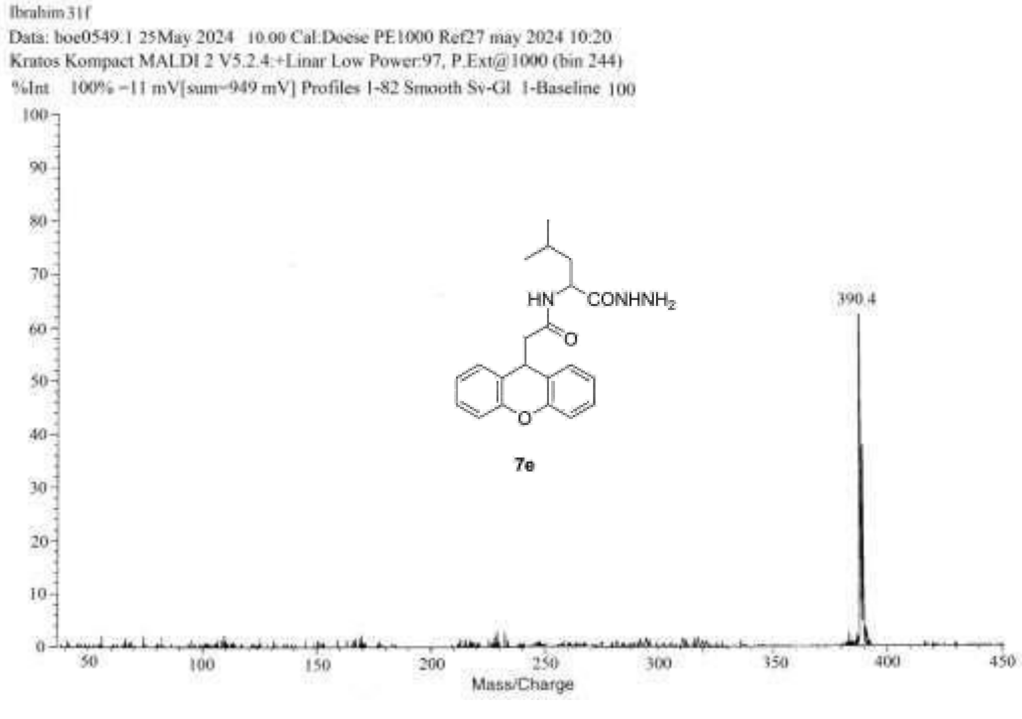

Figure S66. MALDI of compound **7e**.

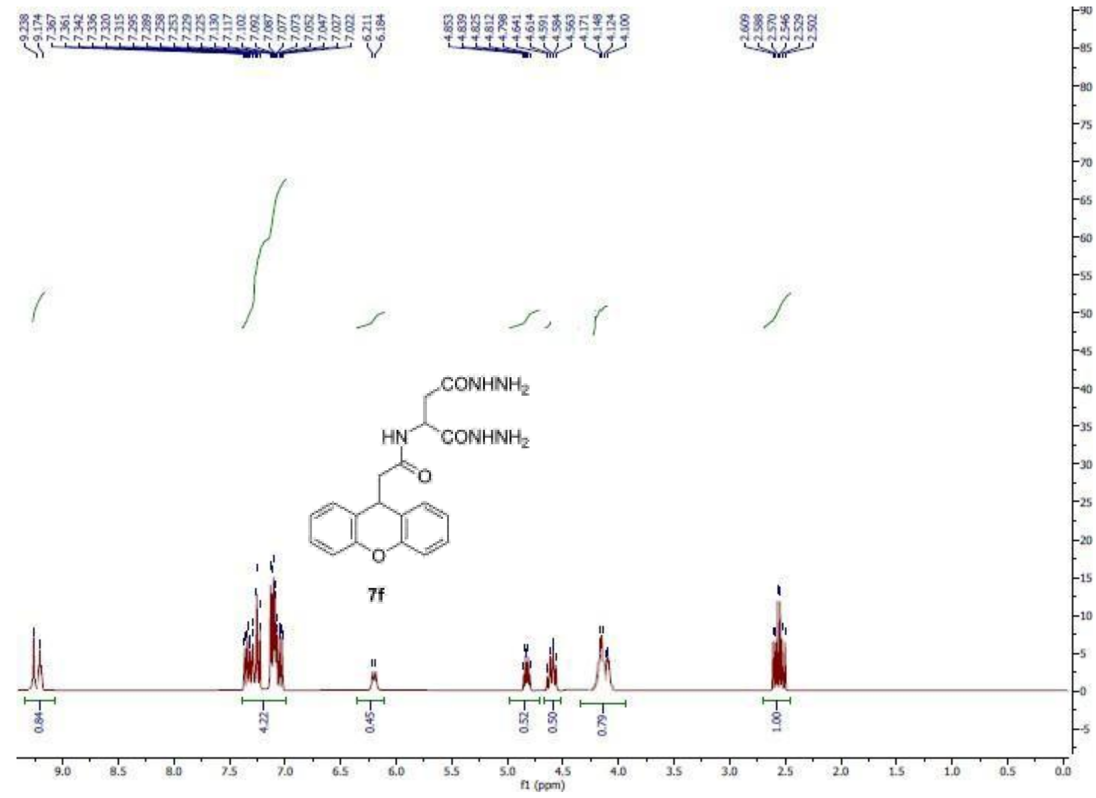

Figure S67. ^{1}H NMR of **7f**.

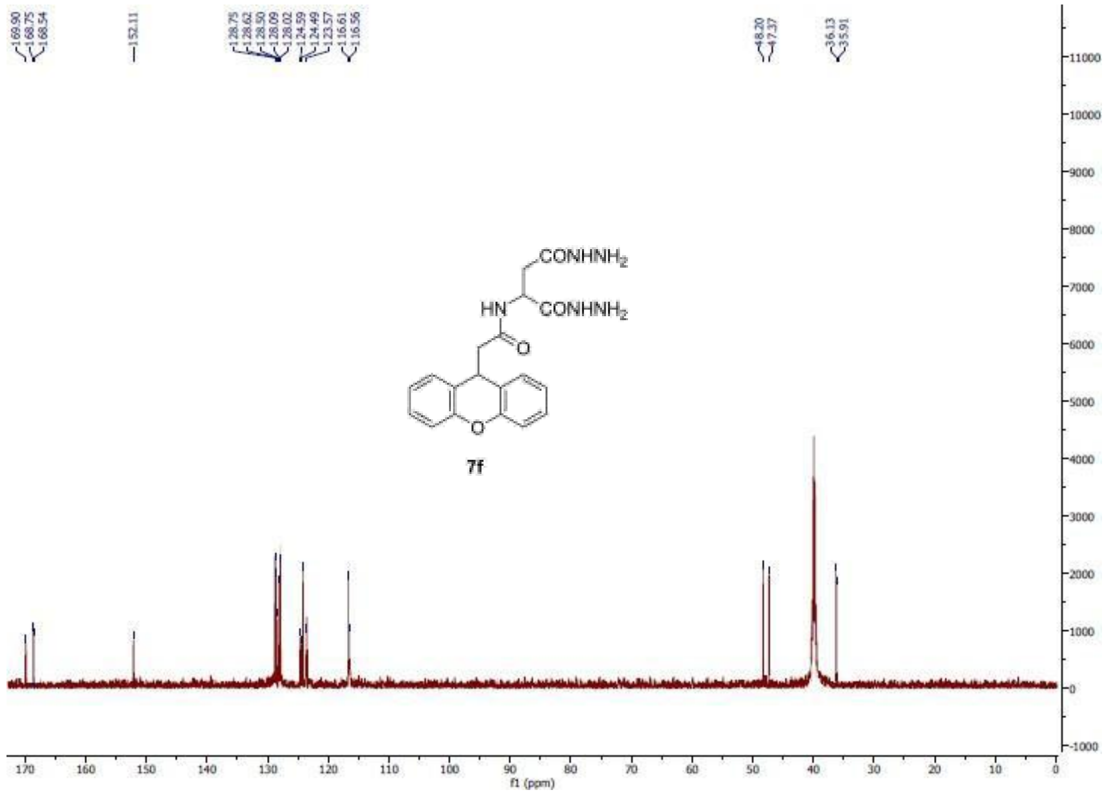

Figure S68. ^{13}C NMR of **7f**.

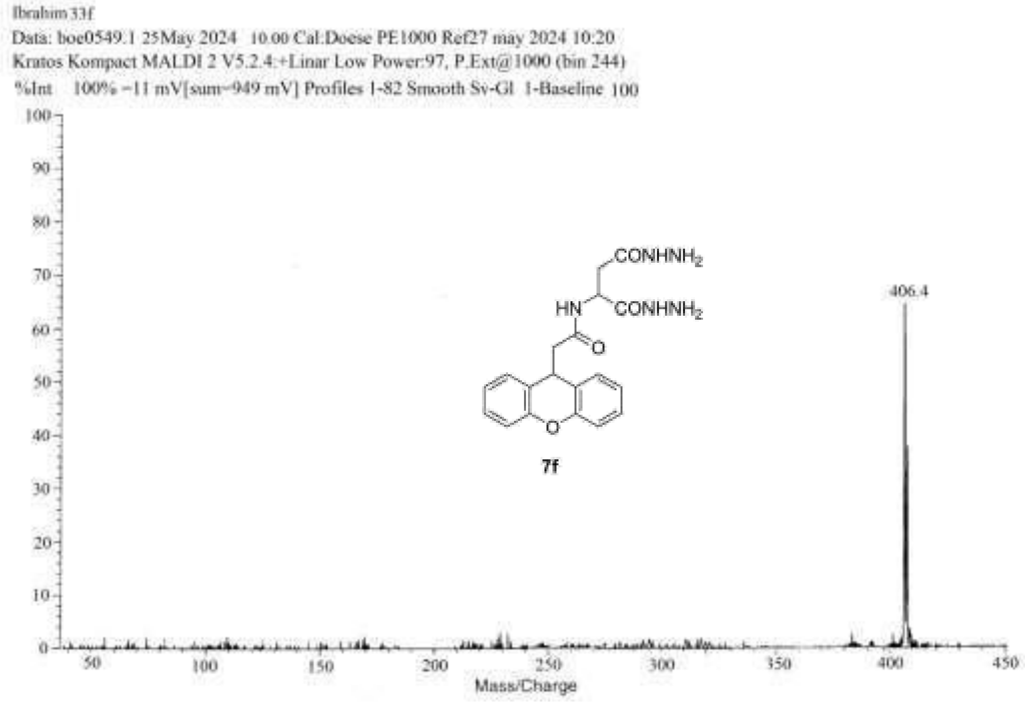

Figure S69. MALDI of compound **7f**.

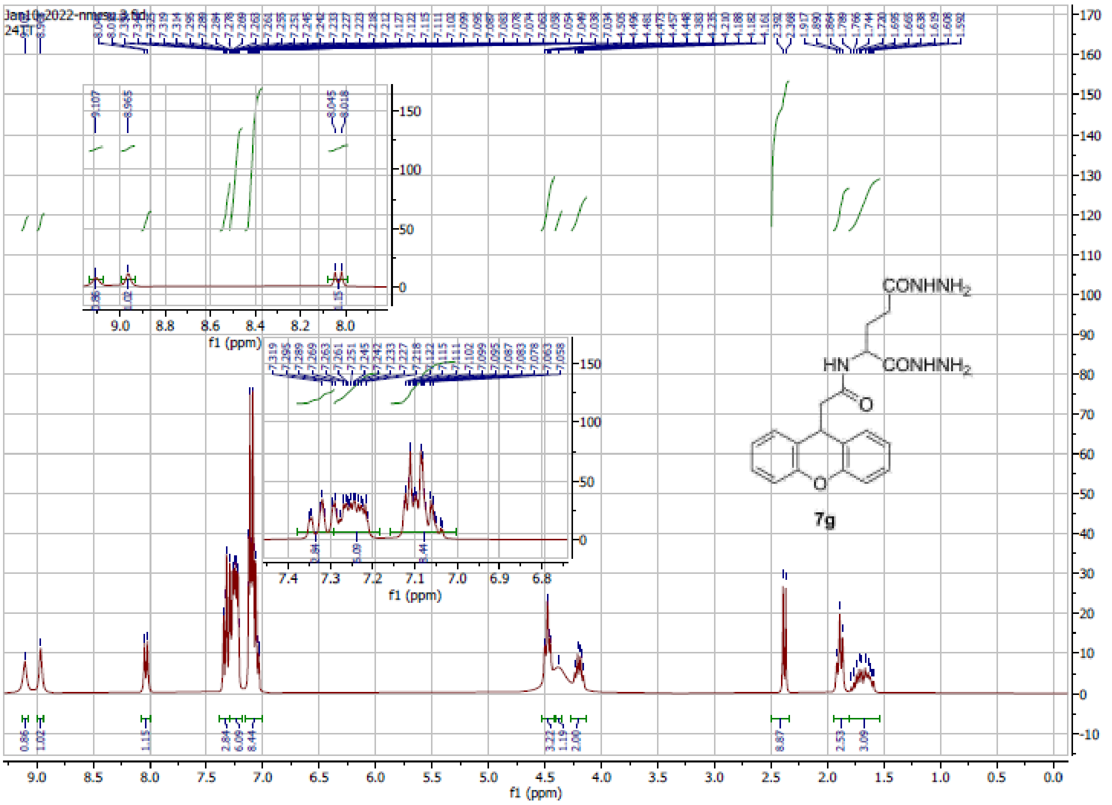

Figure S70. ^{1}H NMR of **7g**.

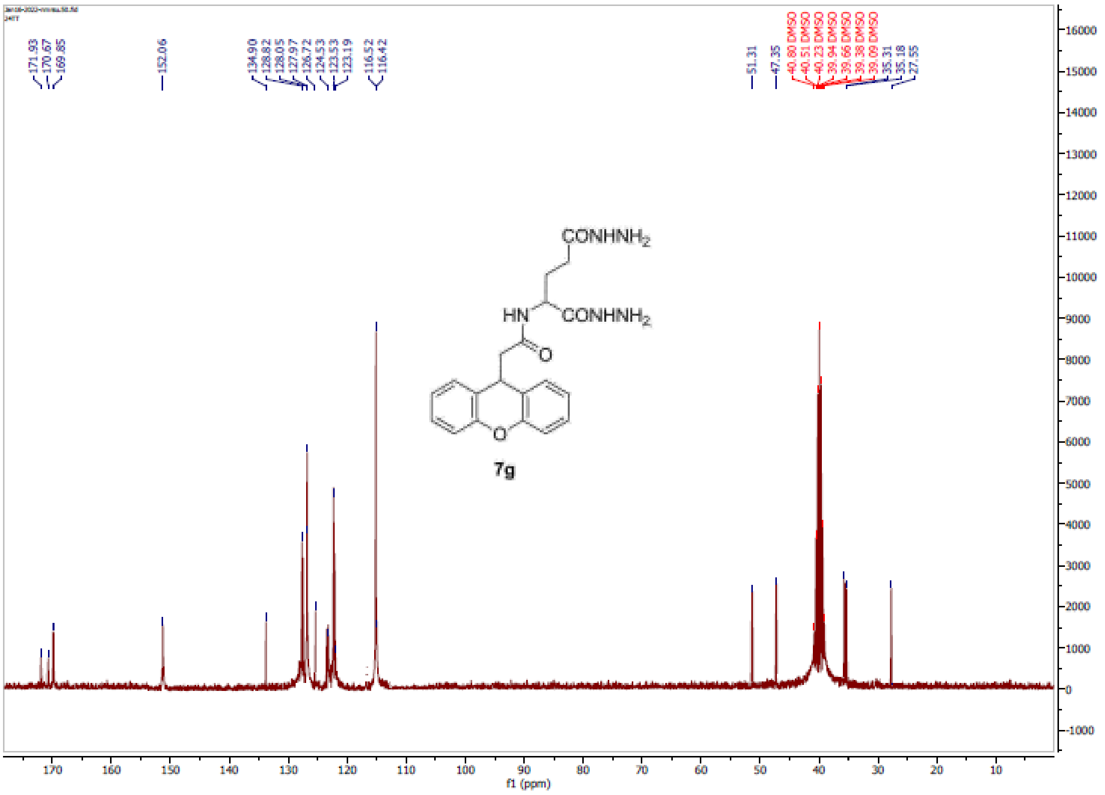

Figure S71. ^{13}C NMR of **7g**.

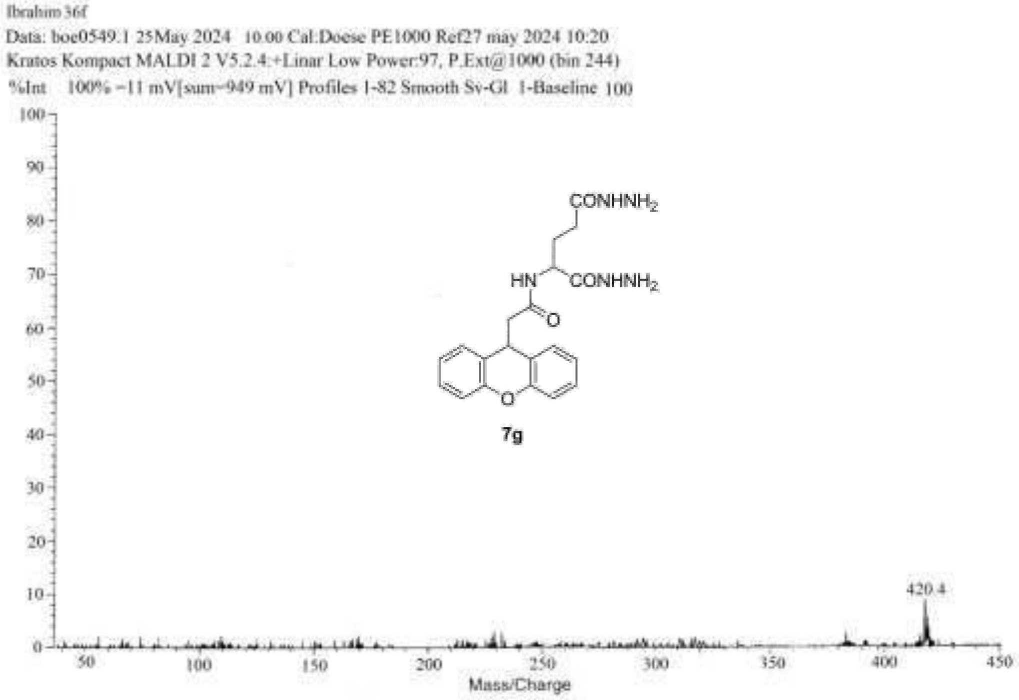

Figure S72. MALDI of compound **7g**.

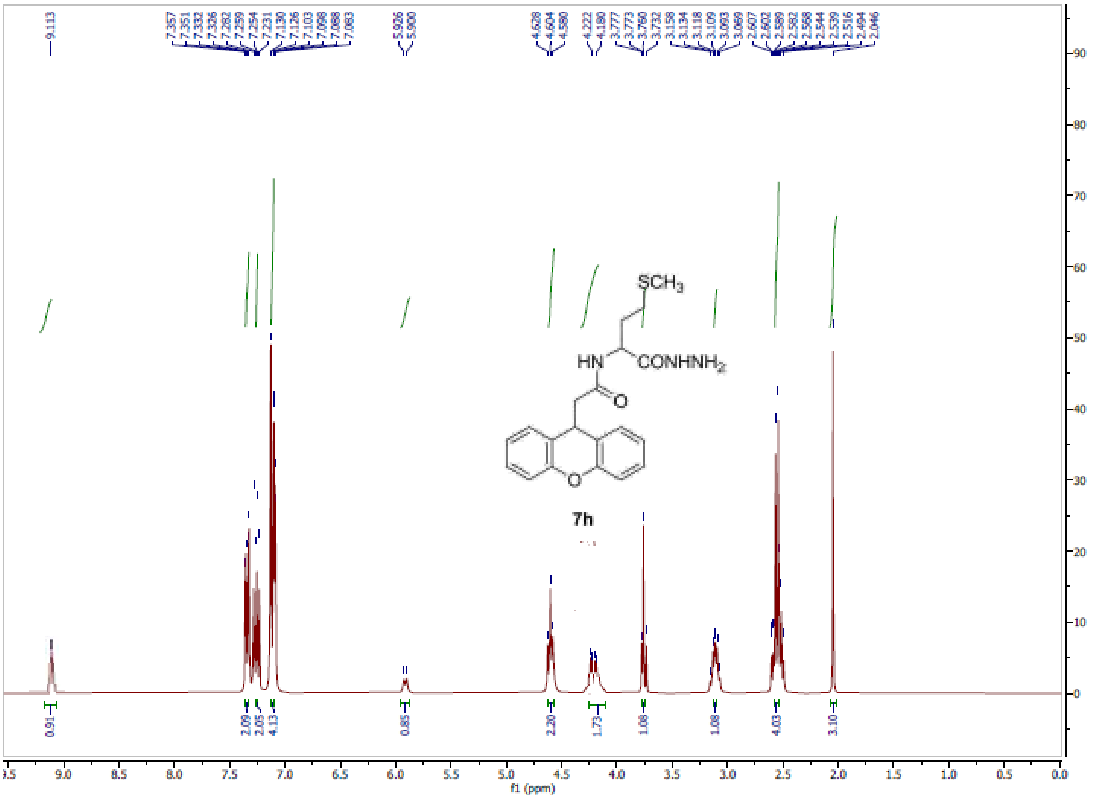

Figure S73. ^{1}H NMR of **7h**.

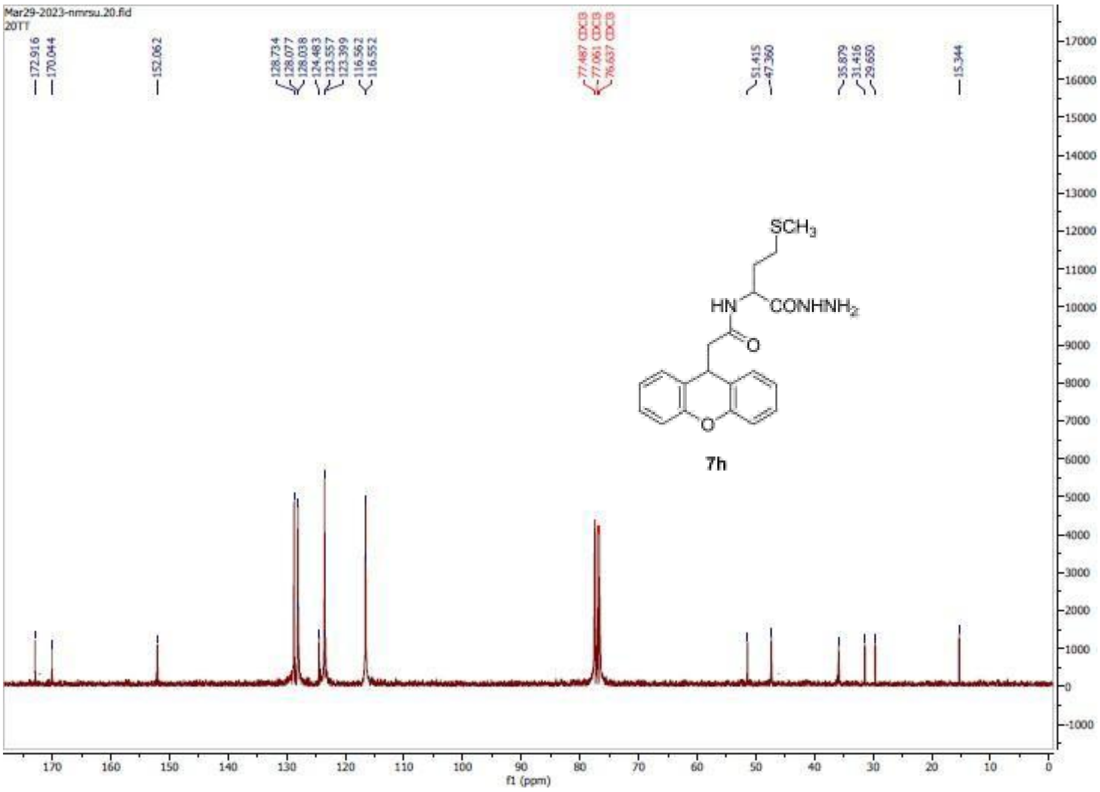

Figure S74. ^{13}C NMR of **7h**.

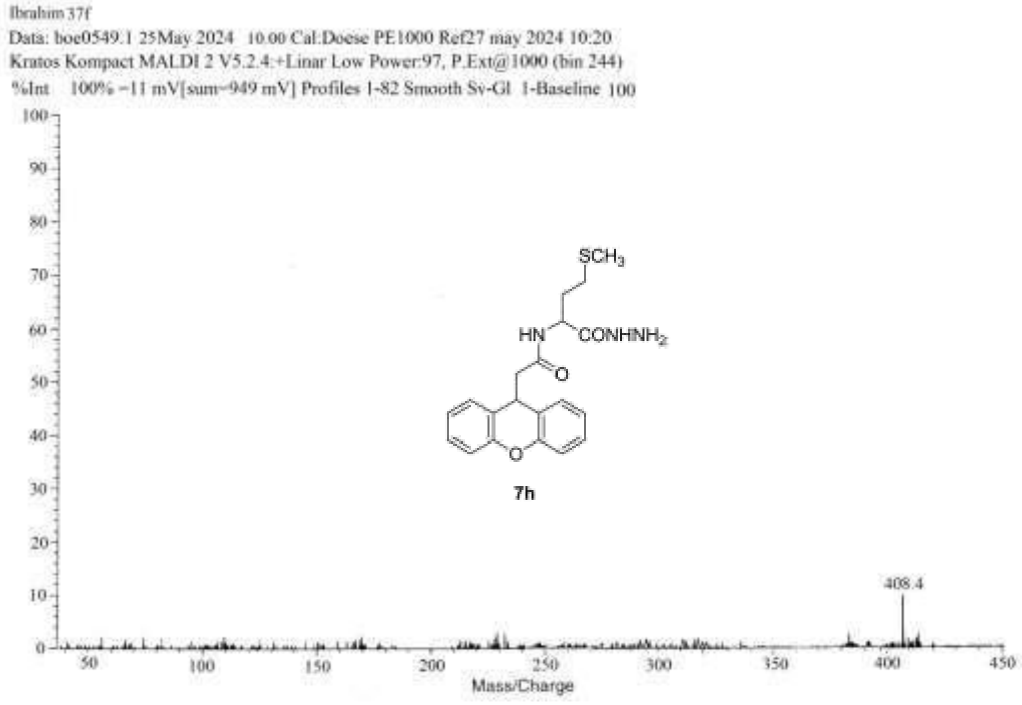

Figure S75. MALDI of compound **7h**.
